# Supplementary material for: Transcriptome Adaptation of the Ovine Mammary Gland to Dietary Supplementation of Extruded Linseed
Source: Animals (Basel). 2021 Sep 16;11(9):2707. doi: 10.3390/ani11092707 (PMC8465498; doi:10.3390/ani11092707)

Supplementary File - for Online Publication Only

Transcriptome adaptation of the ovine mammary gland to dietary supplementation of extruded linseed.

G. Conte, T. Giordani, A. Vangelisti, A Serra, M. Pauselli, A. Cavallini, and M. Mele

Supplementary Figures and Tables

Table S1. Ingredients (g/kg as fed) of the experimental diets used.

Ingredients	Experimental concentrates	
	C[1]	L[2]
Wheat bran	245.0	273.0
Corn	100.0	31.0
Horse bean	100.0	31.0
Sunflower meal	50.0	10.0
Corn gluten	30.0	10.0
Dehydrated alfalfa	200.0	273.0
Barley	200.0	97.0
Molasses	50.0	50.0
Extruded linseed	-	200.0
Calcium carbonate	10.0	10.0
Sodium bicarbonate	5.0	5.0
Di-calcium phosphate	5.0	5.0
Sodium chloride	5.0	5.0

1 C = control diet; 2 L = linseed diet

Table S2. Chemical composition (g/kg of DMI) of the experimental diets used in this trial.

	**Alfalfa hay**	**Rolled barley**	**Experimental concentrates**	
			**C[2]**	**L[3]**
Chemical composition (g / kg of DM[1])				
CP	139.3	118.6	183.8	184.6
EE	20.5	34.4	25.0	92.5
NDF	583.9	269.3	284.0	320.0
ADF	427.0	102.5	142.2	172.4
ADL	92.5	24.4	31.2	42.1
Ash	79.4	26.8	82.9	93.4
ME (Mcal/kg DM)	1.71	3.02	2.62	2.71
FA (% of total FAME)				
C16:0	32.2	26.7	11.9	8.0
C16:1	1.7	0.7	0.1	0.2
C18:0	6.1	4.3	4.8	2.9
C18:1 c9	5.4	14.5	20.8	37.6
C18:2 n6	24.0	32.2	53.8	16.8
C18:3 n3	30.6	21.5	8.6	34.5

Table S3. List of differential expressed genes.

NCBI genes id	Log Fold Change	FDR corrected *P*value	Gene regulation
XM_027967101.1	14.05616687	3.28E-10	Over expressed
XM_027957158.1	13.67390467	0.13799039	Over expressed
XM_004004705.4	11.95577692	3.50E-13	Over expressed
XM_027965085.1	11.46736611	0.017406573	Over expressed
XM_027978824.1	11.4354444	0.009350713	Over expressed
XM_027966087.1	11.35426498	1.80E-05	Over expressed
XM_027979501.1	11.32282633	0.004119139	Over expressed
XM_012095243.1	10.82849696	0.008933872	Over expressed
XM_027978067.1	10.69450561	0.000257873	Over expressed
XM_027965192.1	10.63490619	3.51E-08	Over expressed
XM_027966613.1	10.58320356	2.56E-16	Over expressed
XM_012097000.3	10.56624121	1.73E-14	Over expressed
XM_012149537.3	10.52613001	0.000146986	Over expressed
XM_027979712.1	10.47812946	0.008805434	Over expressed
XM_027974155.1	10.46185335	1.20E-11	Over expressed
XM_027972106.1	10.31292868	0.146691955	Over expressed
XM_027980134.1	10.30886342	5.70E-22	Over expressed
XM_027959920.1	10.28703246	0.008785226	Over expressed
XM_012174558.2	10.17772845	0.146406744	Over expressed
XM_027969732.1	10.1657049	1.20E-08	Over expressed
XM_012173801.3	10.16375176	0.007413789	Over expressed
XM_004015216.4	10.15381106	0.022182876	Over expressed
XM_012174640.3	10.10581855	0.009047483	Over expressed
XM_027963795.1	10.09688752	1.04E-05	Over expressed
XM_027980188.1	10.08989798	0.008712863	Over expressed
XM_027972829.1	10.05168433	0.010209275	Over expressed
XM_015093298.2	9.950470464	0.129429789	Over expressed
XM_027956569.1	9.950202188	0.002627609	Over expressed
XM_012185973.3	9.740319855	0.138546134	Over expressed
XM_015104213.2	9.735869109	0.139119458	Over expressed
XM_012098288.2	9.73328275	0.023239185	Over expressed
XM_012183770.2	9.706270178	0.003904494	Over expressed
XM_027971826.1	9.702608697	0.008622725	Over expressed
XM_027963614.1	9.622091744	0.005604369	Over expressed
XM_012102877.3	9.437729075	0.050774793	Over expressed
XM_027961048.1	9.413650216	0.148077401	Over expressed
XM_027967045.1	9.405387627	0.007215849	Over expressed
XM_012186074.3	9.370320467	0.007398033	Over expressed
XM_012105738.3	9.328924973	0.139801983	Over expressed
XM_027970021.1	9.305554181	0.033731472	Over expressed
XM_027965670.1	9.264462874	0.10105908	Over expressed

XM_015099223.2	9.218145869	0.02022809	Over expressed
XM_015096959.2	9.202470179	6.95E-06	Over expressed
XM_027967689.1	9.196267482	2.48E-06	Over expressed
XM_027959171.1	9.192589259	0.007163966	Over expressed
XM_004022954.4	9.158777196	0.009155017	Over expressed
XM_027968878.1	9.084524624	4.33E-11	Over expressed
XM_027971137.1	9.019185077	0.12727726	Over expressed
XM_027974499.1	8.997430857	0.146069833	Over expressed
XM_027974058.1	8.99613291	1.62E-24	Over expressed
XM_015099862.2	8.965082065	0.128485516	Over expressed
XM_027965031.1	8.947551024	0.147177564	Over expressed
XM_012179334.3	8.943887249	2.22E-13	Over expressed
XM_015095779.2	8.908423954	0.003935723	Over expressed
XM_027974942.1	8.906889546	6.95E-06	Over expressed
XR_001435201.2	8.800991149	0.011331293	Over expressed
XM_027964204.1	8.755091798	0.029824081	Over expressed
XM_004004560.3	8.731324517	8.55E-06	Over expressed
XM_027978691.1	8.722323104	0.005802086	Over expressed
XM_027970155.1	8.707532363	0.003330493	Over expressed
XM_027957585.1	8.689229982	0.011116972	Over expressed
XM_027965300.1	8.669154586	0.018226466	Over expressed
XR_001041614.3	8.661999774	0.000111703	Over expressed
XM_027962107.1	8.627424125	0.004019574	Over expressed
XM_012098874.3	8.61825285	0.128794307	Over expressed
XM_027968179.1	8.613484788	0.140216823	Over expressed
XM_027968784.1	8.611652415	0.006635075	Over expressed
XM_027966208.1	8.577781713	0.143047857	Over expressed
XM_027975324.1	8.454369294	0.130933705	Over expressed
XM_027967409.1	8.422260602	0.006164355	Over expressed
XM_027958906.1	8.364310923	0.018447967	Over expressed
XM_027956878.1	8.329888987	0.013022257	Over expressed
XM_027959957.1	8.317604408	0.144023509	Over expressed
XM_027961403.1	8.256155632	0.128962639	Over expressed
XM_012107696.2	8.208222335	0.140198803	Over expressed
XM_027973197.1	8.173239375	0.030572208	Over expressed
XM_012107672.2	8.152652198	0.144023509	Over expressed
XM_027973514.1	8.128216679	0.013372331	Over expressed
XM_012175689.3	8.110851014	1.56E-14	Over expressed
XM_027962432.1	8.050082692	0.008792602	Over expressed
XM_027961266.1	8.022753864	1.43E-23	Over expressed
XM_027970502.1	7.959372127	0.001507388	Over expressed
XM_027976799.1	7.956330998	0.01828691	Over expressed
XM_027975334.1	7.943116601	3.27E-05	Over expressed
XM_012181601.2	7.908168109	1.43E-23	Over expressed

XM_012099819.3	7.854196552	0.127903557	Over expressed
XM_027967320.1	7.82646508	0.005741206	Over expressed
XM_027965384.1	7.792380779	0.020099076	Over expressed
XM_027976260.1	7.642225396	0.040066284	Over expressed
XM_027973376.1	7.640529849	0.009903269	Over expressed
XM_027961773.1	7.603291905	0.129296313	Over expressed
XM_027959550.1	7.595845018	2.03E-07	Over expressed
XM_027964282.1	7.593642293	0.017658529	Over expressed
XM_027958140.1	7.57921332	0.083570707	Over expressed
XM_004011297.4	7.576674062	0.14598292	Over expressed
XM_027978798.1	7.528014733	0.021421875	Over expressed
XM_027959543.1	7.519025675	0.138756575	Over expressed
XM_027977104.1	7.513454172	2.46E-07	Over expressed
XM_012181362.3	7.445311359	0.139019656	Over expressed
XM_027956499.1	7.39649105	0.001141103	Over expressed
XM_004002634.4	7.370377573	0.000146787	Over expressed
XR_001023602.3	7.328368925	0.124851399	Over expressed
XM_004012668.4	7.282480196	0.001296987	Over expressed
XM_027961197.1	7.277492596	0.061008198	Over expressed
XM_015093423.2	7.203439145	0.147646218	Over expressed
XM_027978639.1	7.186964621	0.128334757	Over expressed
XM_027966610.1	7.096713856	0.001910145	Over expressed
XM_027970173.1	7.06628043	0.014706451	Over expressed
XM_027969820.1	7.04290962	0.147632137	Over expressed
XM_012120593.3	6.995424169	1.25E-11	Over expressed
XM_027966158.1	6.897505202	0.023598824	Over expressed
XM_027958988.1	6.88803505	0.00090434	Over expressed
XM_027978610.1	6.885381514	0.005349469	Over expressed
XM_027970480.1	6.869162994	0.01380001	Over expressed
XM_027974699.1	6.86322605	0.001160346	Over expressed
XM_012106597.3	6.832892928	1.19E-06	Over expressed
XM_012190383.3	6.822474241	0.123193872	Over expressed
XM_012095505.3	6.800424252	0.063321285	Over expressed
XM_027956922.1	6.723218677	0.00652663	Over expressed
XM_012169224.3	6.721995012	0.003624264	Over expressed
XR_003589130.1	6.705777749	0.016899885	Over expressed
XM_027970424.1	6.699726235	0.05608977	Over expressed
XM_027969405.1	6.685350113	0.142857232	Over expressed
XM_027978603.1	6.533814554	0.062748005	Over expressed
XM_012173665.3	6.451115405	0.044694607	Over expressed
XM_027978095.1	6.440257266	0.122511419	Over expressed
XM_012184557.3	6.362498761	0.123211423	Over expressed
XM_012190414.3	6.329016758	0.043432759	Over expressed
XM_012096925.3	6.29843407	0.070637281	Over expressed

XM_027961343.1	6.292746486	0.049811443	Over expressed
XR_003587966.1	6.211426399	0.049772275	Over expressed
XM_027956441.1	6.113936273	7.61E-11	Over expressed
XM_027968258.1	6.026262219	0.003282492	Over expressed
XM_004018362.4	6.012168031	0.020781231	Over expressed
NM_001282138.1	5.980377648	0.006130053	Over expressed
XM_027961424.1	5.920551842	0.01225965	Over expressed
XR_003589614.1	5.917453803	0.050363079	Over expressed
XM_027973558.1	5.874393067	0.035322497	Over expressed
XM_027975158.1	5.872938045	0.023509551	Over expressed
NM_001126366.1	5.856077776	0.000652139	Over expressed
XM_027967114.1	5.839411314	0.093221108	Over expressed
XM_012130264.3	5.753281327	6.18E-06	Over expressed
XM_015101286.2	5.733599322	0.000642333	Over expressed
XM_027959712.1	5.645987355	0.032858196	Over expressed
XM_027969547.1	5.636788416	2.35E-19	Over expressed
XR_003588037.1	5.625750957	0.046968184	Over expressed
XM_012178324.2	5.605979834	0.085925719	Over expressed
XM_027960692.1	5.573178919	0.02166044	Over expressed
XR_003587153.1	5.516751459	0.039406816	Over expressed
XM_027973357.1	5.462584664	0.134061092	Over expressed
XM_027969947.1	5.451310302	0.086907817	Over expressed
XM_004004082.4	5.355237442	0.141543272	Over expressed
XM_012096791.2	5.31742382	0.084035725	Over expressed
XM_015100284.2	5.298419977	0.034005553	Over expressed
XM_027968588.1	5.296593361	0.131808116	Over expressed
XM_027962547.1	5.269127952	0.129080677	Over expressed
XM_004015398.4	5.22974707	0.129167027	Over expressed
XR_003586219.1	5.220215638	0.06431498	Over expressed
XM_027957558.1	5.18859248	0.03817186	Over expressed
XM_012103032.3	5.179651718	0.024990657	Over expressed
XM_027979483.1	5.124414697	0.094282709	Over expressed
XM_012145933.3	5.081013143	0.064733848	Over expressed
XM_027978764.1	5.074088839	0.005249634	Over expressed
XM_015103390.2	5.060668972	0.083315722	Over expressed
XM_027974427.1	5.000683469	0.134961107	Over expressed
XM_027959083.1	4.952375849	0.081729291	Over expressed
XM_027962104.1	4.887547525	0.027580726	Over expressed
XM_027973351.1	4.870750121	0.104787921	Over expressed
XM_027959921.1	4.845727343	0.125073826	Over expressed
XM_004015395.3	4.80397937	0.032574103	Over expressed
XM_027967970.1	4.7982733	0.044895897	Over expressed
XM_027965538.1	4.754432791	0.108619376	Over expressed
XM_012189971.2	4.682000668	1.59E-09	Over expressed

XR_003586398.1	4.670107765	0.097737873	Over expressed
XM_027967408.1	4.670042888	0.147665985	Over expressed
XM_027978849.1	4.664969074	0.125724353	Over expressed
XM_012178039.3	4.582745309	0.071909275	Over expressed
XM_012187144.2	4.531465237	0.127437844	Over expressed
NM_001009774.3	4.497633009	0.024261056	Over expressed
XM_027968321.1	4.451221053	0.004716716	Over expressed
XM_027980275.1	4.420074226	0.003450801	Over expressed
XM_027977436.1	4.41688048	0.042807627	Over expressed
XM_012096405.3	4.404579219	0.003836091	Over expressed
XM_012102718.3	4.403700189	0.127289261	Over expressed
XM_004018431.4	4.402739575	0.049002159	Over expressed
XM_027975116.1	4.397440565	0.100569221	Over expressed
XM_027974521.1	4.396219802	0.028011135	Over expressed
XM_027961288.1	4.380229537	0.003527047	Over expressed
XM_027965388.1	4.339713307	0.093173579	Over expressed
XM_027973480.1	4.331977521	0.007413789	Over expressed
XM_012190181.3	4.323434098	0.134188408	Over expressed
XM_027962679.1	4.316820022	0.112206663	Over expressed
XR_001434329.2	4.310865036	0.009470702	Over expressed
XM_027966147.1	4.285531641	0.136552173	Over expressed
NR_107953.1	4.273827377	0.088608902	Over expressed
XM_027956657.1	4.226702443	0.000312328	Over expressed
XM_012188703.2	4.183173123	0.031828754	Over expressed
XM_012098716.3	4.170989637	0.01556651	Over expressed
XR_003588432.1	4.148019549	0.037413702	Over expressed
XM_027966819.1	4.135158925	0.069503187	Over expressed
XM_012102859.2	4.1289564	0.086606981	Over expressed
XM_015104664.2	4.104440306	0.010527307	Over expressed
XM_027958399.1	4.09627443	0.018061258	Over expressed
XM_027966103.1	4.078083004	0.147499337	Over expressed
XM_012185671.3	4.049443561	0.019705513	Over expressed
XM_027956882.1	4.011094754	0.073304131	Over expressed
XM_027956937.1	3.98219055	0.102526499	Over expressed
XM_027966136.1	3.960754683	0.047872437	Over expressed
XM_027974448.1	3.936282837	0.010890013	Over expressed
XM_027963835.1	3.924692469	0.043400475	Over expressed
XM_027965917.1	3.913522054	0.122562539	Over expressed
XM_027975093.1	3.899084543	0.046931213	Over expressed
XM_027967762.1	3.883759781	0.00439289	Over expressed
XM_027959818.1	3.873080984	0.004976659	Over expressed
XM_004008129.4	3.851895116	0.014255666	Over expressed
XM_027962131.1	3.798084115	0.087873872	Over expressed
XM_027957859.1	3.795559685	0.001797027	Over expressed

XM_012185695.3	3.776090768	0.061431822	Over expressed
XM_027967506.1	3.751879445	0.080911067	Over expressed
XM_027974124.1	3.751054622	9.24E-08	Over expressed
XM_015100027.2	3.750284002	0.000213057	Over expressed
XM_027974501.1	3.740975248	0.00119733	Over expressed
XR_003590846.1	3.740206	0.010237078	Over expressed
XM_027957281.1	3.726118358	0.109242473	Over expressed
XR_003588878.1	3.7254887	0.093765712	Over expressed
XM_012183804.1	3.717403168	0.042247525	Over expressed
XR_003589064.1	3.7041914	0.086197018	Over expressed
XM_015098545.2	3.703344533	0.105217377	Over expressed
XM_027959636.1	3.70150094	0.112444848	Over expressed
XM_027968059.1	3.700665595	0.047872437	Over expressed
XM_027975514.1	3.697735582	0.000896743	Over expressed
NM_001162545.1	3.687689812	0.090169349	Over expressed
XM_027979792.1	3.672240114	2.09E-05	Over expressed
XM_027968998.1	3.670245443	0.093173579	Over expressed
XM_027957472.1	3.666150539	0.071849297	Over expressed
XM_027969379.1	3.63886024	0.056350326	Over expressed
XM_015100290.2	3.627046263	0.004963364	Over expressed
XM_027978314.1	3.595328716	0.050774793	Over expressed
XM_027977999.1	3.593855599	0.020762164	Over expressed
XM_027974502.1	3.588914239	0.146817476	Over expressed
XM_004012742.4	3.582512144	0.057072535	Over expressed
XM_027971415.1	3.573117695	0.095164789	Over expressed
XM_027961262.1	3.566285438	0.114276131	Over expressed
XM_027962249.1	3.550320613	0.01035351	Over expressed
XM_027960842.1	3.548787401	0.021421875	Over expressed
XR_001435442.2	3.524420159	0.036080406	Over expressed
XM_027969619.1	3.521965054	0.068576147	Over expressed
XM_012190684.3	3.516002568	0.013093738	Over expressed
XM_027965559.1	3.50938903	0.011815879	Over expressed
XM_012177565.3	3.503467721	0.118281968	Over expressed
XM_027960291.1	3.499228857	6.87E-06	Over expressed
XR_003588172.1	3.494792752	0.084467286	Over expressed
XM_012098663.3	3.489304418	0.067797253	Over expressed
XM_012126652.3	3.473682287	0.050135514	Over expressed
XM_027969549.1	3.46427637	9.01E-05	Over expressed
XM_027975974.1	3.455913932	0.051417105	Over expressed
XM_027967173.1	3.432413847	0.004734909	Over expressed
XM_012158633.3	3.431992047	0.147381143	Over expressed
XM_004015352.4	3.428725449	0.052261732	Over expressed
XM_027976050.1	3.411243815	0.016863613	Over expressed
XR_003586392.1	3.400384735	6.35E-08	Over expressed

XM_027966462.1	3.39007942	0.066977628	Over expressed
XM_027967996.1	3.385290514	0.005346166	Over expressed
XM_027975669.1	3.385130991	0.135789221	Over expressed
XM_027962405.1	3.378231972	6.87E-06	Over expressed
XR_003585528.1	3.372177722	9.89E-11	Over expressed
XM_027974057.1	3.369411389	0.104618029	Over expressed
XM_027970397.1	3.367947921	0.051225037	Over expressed
XR_003586124.1	3.36747672	0.097704543	Over expressed
XR_003589866.1	3.359881247	0.001825172	Over expressed
XM_004023496.4	3.354842123	0.000646533	Over expressed
XM_027971322.1	3.352857617	0.128596432	Over expressed
XM_027978458.1	3.331535521	0.010662357	Over expressed
XM_027962743.1	3.308642168	0.133471132	Over expressed
XM_027978860.1	3.302190054	6.09E-05	Over expressed
XM_027963525.1	3.28791888	0.024372266	Over expressed
XM_027968291.1	3.282964205	0.01727368	Over expressed
XM_027969756.1	3.275807319	0.040611246	Over expressed
XM_027968789.1	3.270360763	0.003720507	Over expressed
XM_004017510.3	3.269206892	0.002641912	Over expressed
XM_012174650.2	3.261131096	0.017406573	Over expressed
XM_015102282.2	3.256553091	0.130933705	Over expressed
XM_004015234.3	3.224965371	0.097704543	Over expressed
XM_027961646.1	3.198506995	0.101316526	Over expressed
XM_015101004.2	3.191118441	0.019558062	Over expressed
XM_027960007.1	3.172344544	0.000163787	Over expressed
XM_015094858.2	3.16161988	0.008617917	Over expressed
XR_003591641.1	3.158048778	0.044718896	Over expressed
XM_027959673.1	3.157296431	0.001770161	Over expressed
XM_027971275.1	3.136928502	0.010237078	Over expressed
XM_027963765.1	3.136659793	0.125073826	Over expressed
XM_027977395.1	3.127686898	0.000183562	Over expressed
XM_027978099.1	3.124653674	0.05866608	Over expressed
XM_004015233.4	3.123571506	0.122693503	Over expressed
XM_012181882.3	3.122179935	0.036282311	Over expressed
XM_027965229.1	3.115432965	2.76E-09	Over expressed
XM_027977519.1	3.110120151	0.099181007	Over expressed
XM_004019688.4	3.103558285	0.131249752	Over expressed
XM_027969860.1	3.097342885	0.06263427	Over expressed
XM_027973944.1	3.094520671	0.003061434	Over expressed
XM_027977407.1	3.089298564	0.069328983	Over expressed
XM_027967938.1	3.087621261	0.001020787	Over expressed
NR_107962.1	3.086737464	0.052730363	Over expressed
XM_027977163.1	3.085391146	0.018200669	Over expressed
XM_027961107.1	3.084900377	0.115808202	Over expressed

XM_027978459.1	3.080753084	0.000209246	Over expressed
XM_027957877.1	3.080175554	0.000853046	Over expressed
XM_015092222.2	3.075203176	5.13E-05	Over expressed
XM_027962624.1	3.074234348	0.031308523	Over expressed
XM_027979831.1	3.04936248	0.064873365	Over expressed
XM_027959254.1	3.043439869	1.52E-12	Over expressed
XM_004010380.3	3.037028546	0.130933705	Over expressed
NM_001009220.1	3.036222653	0.097574786	Over expressed
XM_027961468.1	3.018784943	5.84E-05	Over expressed
XM_012131679.3	3.018010404	0.043359338	Over expressed
NM_001139448.1	3.016979993	0.025010063	Over expressed
XM_027961369.1	3.012031454	0.007234519	Over expressed
NM_001009436.1	2.999268627	0.080676102	Over expressed
XM_027975447.1	2.99890635	0.000674865	Over expressed
XR_003590817.1	2.99694815	0.026127568	Over expressed
XM_027976055.1	2.994939371	0.115642766	Over expressed
XM_027959977.1	2.992641796	0.010297484	Over expressed
XM_027966616.1	2.984682166	0.038616448	Over expressed
XM_027963220.1	2.966273243	0.038453148	Over expressed
XM_012175808.3	2.962118612	0.000113376	Over expressed
XM_027974649.1	2.953976136	0.004979877	Over expressed
XM_012174787.2	2.952730243	0.06847315	Over expressed
XM_027972878.1	2.952538117	0.01867031	Over expressed
XM_012149891.3	2.948825242	1.54E-05	Over expressed
XM_027967758.1	2.948496927	0.059454582	Over expressed
XM_027969457.1	2.944347395	0.030793443	Over expressed
XM_027978899.1	2.934500943	0.000666349	Over expressed
XM_027977795.1	2.932125638	0.019529324	Over expressed
XM_012105513.2	2.926950135	0.00040445	Over expressed
XM_027962322.1	2.926444877	0.018337655	Over expressed
XM_027968157.1	2.923397709	0.141322302	Over expressed
XM_012166381.3	2.921350142	0.043808817	Over expressed
XM_012178435.2	2.919602941	0.000235346	Over expressed
XM_027959581.1	2.918553534	1.14E-05	Over expressed
XM_015095645.2	2.912897714	0.011554037	Over expressed
NM_001078655.1	2.911264659	0.000116674	Over expressed
XM_027960006.1	2.911012924	0.065677688	Over expressed
XM_027961244.1	2.910420316	0.022772139	Over expressed
XM_004018522.3	2.90866814	0.01330496	Over expressed
XM_015100117.2	2.906903763	0.015923826	Over expressed
XM_027973217.1	2.89848384	0.000638215	Over expressed
XM_027975307.1	2.896358734	0.005239998	Over expressed
XM_027958867.1	2.888864719	0.114700133	Over expressed
XM_015097574.2	2.887451412	0.130933705	Over expressed

XM_027968702.1	2.887022703	0.002254399	Over expressed
XM_027966134.1	2.885192155	0.004183811	Over expressed
XM_027966794.1	2.882859171	0.007127445	Over expressed
XM_015096020.2	2.882553083	0.006047766	Over expressed
XM_027970661.1	2.880087944	0.004790066	Over expressed
XM_027960607.1	2.87760216	0.089666148	Over expressed
XM_027960275.1	2.87073394	0.000731727	Over expressed
XM_027961894.1	2.869344113	1.60E-05	Over expressed
XM_027966076.1	2.868232161	0.090115683	Over expressed
XM_027979321.1	2.859805092	0.119033215	Over expressed
XM_027960238.1	2.859530154	0.001049084	Over expressed
XM_027959493.1	2.857730234	0.000782958	Over expressed
XM_027961218.1	2.854505574	0.057416218	Over expressed
XM_027979708.1	2.837583468	3.40E-07	Over expressed
XM_004018807.4	2.836494567	0.03639558	Over expressed
XM_027967513.1	2.83529806	0.007234519	Over expressed
XR_003591021.1	2.83173233	0.063617621	Over expressed
XM_027976014.1	2.823523946	0.041008729	Over expressed
XM_004013064.4	2.823377084	0.000163787	Over expressed
XM_027968119.1	2.821517607	0.044142627	Over expressed
XM_027967273.1	2.813180646	0.038093593	Over expressed
NR_107879.1	2.799555352	0.000253302	Over expressed
XM_027962101.1	2.798239049	0.139185896	Over expressed
XM_015096951.2	2.785650022	0.125601875	Over expressed
XR_003590830.1	2.783546282	0.086765688	Over expressed
XM_015103513.2	2.782109269	4.89E-08	Over expressed
XM_027973386.1	2.78135727	0.001928158	Over expressed
XM_004008418.4	2.775540574	0.008383677	Over expressed
XM_004020697.4	2.76623257	0.019823259	Over expressed
XR_003588776.1	2.765447032	0.016999286	Over expressed
XR_003591637.1	2.765165023	0.004225515	Over expressed
XM_027966111.1	2.758164175	0.02762261	Over expressed
XM_027969354.1	2.756632066	0.005553335	Over expressed
XR_001043674.3	2.756291327	0.022395235	Over expressed
XM_027956560.1	2.754781798	0.043621124	Over expressed
XM_027978643.1	2.7536356	0.045713802	Over expressed
XR_003588086.1	2.752438125	0.001859641	Over expressed
XM_027969370.1	2.740690362	3.03E-06	Over expressed
XM_027974044.1	2.73949069	0.000146787	Over expressed
XM_027977587.1	2.737145298	0.005145597	Over expressed
XM_027965631.1	2.733713511	1.42E-06	Over expressed
XM_027970422.1	2.73314546	4.59E-08	Over expressed
NM_001146271.1	2.731605984	0.137930188	Over expressed
XM_027975786.1	2.730680003	0.014992707	Over expressed

XM_027971542.1	2.729870519	0.074093987	Over expressed
XM_012126973.3	2.729634591	0.000716358	Over expressed
XM_027961366.1	2.724082427	0.006440947	Over expressed
XM_027956747.1	2.70821738	0.019881571	Over expressed
XM_027956931.1	2.707915466	7.78E-05	Over expressed
XM_027966096.1	2.705275115	0.002349699	Over expressed
XM_027968308.1	2.702425847	0.000883136	Over expressed
XM_012181723.2	2.695791064	0.000518102	Over expressed
XM_027975869.1	2.691790734	0.00220499	Over expressed
XM_027960831.1	2.685181699	0.000494728	Over expressed
XM_027977585.1	2.682595222	0.05267501	Over expressed
XM_027968219.1	2.670759978	0.077415236	Over expressed
XM_027967570.1	2.669630686	9.65E-05	Over expressed
XM_027973447.1	2.669096315	0.004889329	Over expressed
XM_027976407.1	2.665575773	0.029556502	Over expressed
XM_015100100.2	2.663830946	0.008713435	Over expressed
XM_027969609.1	2.66111258	0.025814029	Over expressed
XM_027978142.1	2.658845318	0.14187072	Over expressed
XM_015103987.2	2.652461638	0.090169349	Over expressed
XM_027968094.1	2.650821652	0.068437798	Over expressed
XM_027966395.1	2.649219502	0.004344478	Over expressed
XM_027966405.1	2.646299701	0.010297484	Over expressed
XR_003587196.1	2.642420946	0.001130604	Over expressed
XM_027978826.1	2.636107495	1.31E-06	Over expressed
XM_027979470.1	2.633306831	0.080309947	Over expressed
XM_004003498.4	2.626418661	0.012910881	Over expressed
XM_027967025.1	2.6257846	0.000214732	Over expressed
XM_012186335.3	2.624719468	0.000171935	Over expressed
XM_027969854.1	2.622925046	0.074207643	Over expressed
XM_027957886.1	2.61445661	5.95E-05	Over expressed
XM_004019223.4	2.612661592	0.001910145	Over expressed
XM_027959377.1	2.607454961	0.000195064	Over expressed
XM_027964670.1	2.607283961	0.066753208	Over expressed
XM_027969394.1	2.602914335	0.004233853	Over expressed
XM_015093830.2	2.601762699	0.00617518	Over expressed
XM_027975553.1	2.600795078	7.50E-10	Over expressed
XM_027959710.1	2.589426171	0.000765534	Over expressed
XM_027956932.1	2.587207843	0.007407886	Over expressed
XM_027959275.1	2.583744456	0.131199935	Over expressed
XM_027976938.1	2.577349698	0.011352421	Over expressed
XM_027970135.1	2.573349936	0.00372592	Over expressed
XM_012185609.2	2.570148815	0.010154235	Over expressed
XM_027978778.1	2.570013545	0.0134182	Over expressed
XM_027956807.1	2.567795878	0.026896125	Over expressed

XM_027969853.1	2.558179861	0.137339498	Over expressed
XM_027971608.1	2.555471876	0.099922906	Over expressed
XM_015097366.2	2.5502416	0.000615929	Over expressed
XM_027957465.1	2.550104854	0.001235093	Over expressed
XM_027968435.1	2.548301106	0.077737502	Over expressed
XM_027956431.1	2.546876046	0.130933705	Over expressed
XM_027966079.1	2.546618454	3.20E-05	Over expressed
NM_001009406.1	2.544315126	0.084518502	Over expressed
XM_027965473.1	2.544010039	0.041978419	Over expressed
XM_027967120.1	2.540128074	0.043063167	Over expressed
XM_015095851.2	2.53980863	0.020417913	Over expressed
XM_027962394.1	2.539329036	0.017658529	Over expressed
XM_015095798.2	2.536574858	0.012538451	Over expressed
XM_015101052.2	2.535271603	0.015161941	Over expressed
XM_027961278.1	2.532797422	0.003335178	Over expressed
XM_027961706.1	2.528416814	3.05E-10	Over expressed
XM_004002319.4	2.518621372	0.063294169	Over expressed
XM_027979520.1	2.518010054	0.102718375	Over expressed
XM_027975794.1	2.514953251	0.004544186	Over expressed
XM_027972755.1	2.512488301	0.032525901	Over expressed
XM_015099797.2	2.507231125	2.99E-05	Over expressed
XM_027976122.1	2.506882517	0.05098375	Over expressed
XM_015092224.2	2.502790416	0.006025018	Over expressed
XM_027969393.1	2.500824574	0.014474016	Over expressed
NM_001166186.1	2.499720198	0.000643223	Over expressed
XM_012119517.3	2.498561863	0.018741233	Over expressed
XM_015100809.2	2.49584364	0.043394599	Over expressed
XM_027962044.1	2.493598715	0.001044745	Over expressed
XM_012095319.3	2.493548751	0.004704199	Over expressed
XM_027959501.1	2.487577131	0.012306933	Over expressed
XM_004002526.4	2.487568436	0.013794559	Over expressed
XM_027963649.1	2.480011403	0.095422899	Over expressed
XM_027957483.1	2.47906451	0.10869922	Over expressed
XM_027958821.1	2.478422585	0.00010221	Over expressed
XM_027956921.1	2.476013352	0.021353308	Over expressed
XM_027960379.1	2.475264902	0.02580157	Over expressed
XM_027970695.1	2.473092063	0.093867612	Over expressed
XM_027974100.1	2.470740296	0.06585707	Over expressed
XM_012098057.3	2.469094435	0.000467742	Over expressed
XM_027957473.1	2.468123186	0.01454658	Over expressed
XM_004003400.4	2.466165367	0.005621843	Over expressed
XR_003588680.1	2.46297987	0.063641717	Over expressed
XM_012174208.3	2.461530253	0.094373585	Over expressed
XM_027956942.1	2.460498763	0.021436219	Over expressed

XM_027966066.1	2.458901557	0.020033532	Over expressed
XM_004005704.3	2.458333829	0.088004748	Over expressed
XM_004003720.4	2.45745536	0.099593916	Over expressed
XR_001436150.2	2.457397421	0.127532501	Over expressed
XM_027969185.1	2.457112378	0.135789221	Over expressed
XM_027959284.1	2.453829888	0.026292077	Over expressed
XM_027957549.1	2.450317423	0.000153308	Over expressed
XM_027967278.1	2.444407344	0.119309115	Over expressed
XM_012095497.2	2.4442692	0.096997656	Over expressed
XM_027977440.1	2.443089592	0.037456646	Over expressed
XM_027959549.1	2.442241007	0.00090475	Over expressed
XM_004023292.4	2.441461087	0.006047766	Over expressed
XM_027975742.1	2.440695472	0.051076808	Over expressed
XM_004008537.3	2.440200747	0.021244802	Over expressed
XM_027959859.1	2.437913628	0.046479751	Over expressed
XM_012179247.3	2.437288618	0.057423749	Over expressed
XM_027972813.1	2.429887711	0.04129278	Over expressed
XM_027961466.1	2.424210503	0.011448767	Over expressed
XM_004019661.4	2.423697067	0.016718906	Over expressed
XM_027956777.1	2.420062496	0.002595607	Over expressed
XM_027966078.1	2.414417421	0.12875644	Over expressed
XM_027978858.1	2.412392263	0.017307158	Over expressed
XM_004006276.4	2.411268238	0.120191014	Over expressed
XM_012190204.3	2.410821664	0.075520292	Over expressed
XM_027969607.1	2.410091776	0.004848344	Over expressed
XM_027957834.1	2.407675828	0.03942363	Over expressed
XM_027956739.1	2.407031904	4.52E-07	Over expressed
XM_027969623.1	2.403632451	0.000943714	Over expressed
XM_027977058.1	2.403114761	0.098828365	Over expressed
XR_003588572.1	2.401376742	0.080643936	Over expressed
XM_012181011.3	2.396841902	0.001129342	Over expressed
XM_027973437.1	2.396378178	0.065677688	Over expressed
XM_015102394.2	2.38552954	0.042776766	Over expressed
XM_027964402.1	2.384933437	0.008154444	Over expressed
XM_027974424.1	2.383069162	3.20E-05	Over expressed
XM_015102960.2	2.383018034	3.33E-13	Over expressed
XM_027973425.1	2.380051486	0.000669454	Over expressed
XM_027961911.1	2.377987376	0.082930467	Over expressed
XM_027959492.1	2.376703051	0.012639426	Over expressed
XM_027960010.1	2.375171489	0.092805918	Over expressed
XM_004005022.4	2.371437897	0.115568862	Over expressed
XM_027976232.1	2.371151518	0.003456153	Over expressed
XM_027967976.1	2.369975569	0.010789577	Over expressed
XM_027968311.1	2.366002908	2.13E-08	Over expressed

XM_012180575.3	2.365073939	0.117748156	Over expressed
NM_001009396.1	2.364726445	0.027450454	Over expressed
XM_027959402.1	2.363316307	1.19E-16	Over expressed
XM_027969608.1	2.358427653	4.21E-05	Over expressed
XM_027963874.1	2.355284344	0.029126185	Over expressed
XM_027975793.1	2.352723242	0.009939424	Over expressed
XM_027974126.1	2.352491732	2.10E-09	Over expressed
XM_027961057.1	2.351360906	0.000195064	Over expressed
XM_004018462.4	2.350119683	0.015139536	Over expressed
XM_004020916.4	2.347963882	0.008201946	Over expressed
XM_004013122.4	2.345847619	0.001316152	Over expressed
XR_003590635.1	2.345786907	0.017741268	Over expressed
XM_027978383.1	2.341988349	0.01953568	Over expressed
XM_004022782.4	2.341176549	0.000168012	Over expressed
XM_012182573.3	2.339896547	0.040686494	Over expressed
XM_027959472.1	2.338689857	0.010209275	Over expressed
XM_027963647.1	2.335578392	0.011286072	Over expressed
XM_027978058.1	2.33507815	0.010154235	Over expressed
XM_027957286.1	2.334331788	0.005610556	Over expressed
XM_027966315.1	2.334225673	0.00211852	Over expressed
NM_001161882.1	2.330966393	0.00115211	Over expressed
XM_027974935.1	2.329931385	0.127925516	Over expressed
XM_027964067.1	2.328803733	4.44E-05	Over expressed
XR_003587307.1	2.326570352	0.04748622	Over expressed
XM_004006731.4	2.323433234	0.022689947	Over expressed
XM_027957159.1	2.32116493	0.141812568	Over expressed
XM_004002523.3	2.318775426	0.003224408	Over expressed
XR_001435313.2	2.31658634	3.27E-05	Over expressed
XM_027966091.1	2.315628493	0.045359478	Over expressed
XM_027971296.1	2.315132631	0.000492242	Over expressed
XM_027959995.1	2.313443978	0.006964631	Over expressed
XM_027964022.1	2.312108502	0.027783282	Over expressed
XM_027978428.1	2.311555919	3.05E-11	Over expressed
XM_027956832.1	2.307156243	0.002524519	Over expressed
XM_027970144.1	2.305198909	2.45E-07	Over expressed
XM_027961856.1	2.304614664	0.064852056	Over expressed
XM_004017391.4	2.297062971	0.008027201	Over expressed
XM_015093628.2	2.290885024	0.117931714	Over expressed
XM_012177833.3	2.290637097	0.000412032	Over expressed
XM_027968239.1	2.288906186	0.032051653	Over expressed
XR_003590599.1	2.286790169	2.29E-05	Over expressed
XM_015098536.2	2.282715018	0.000412032	Over expressed
XM_027966184.1	2.281940431	0.087207655	Over expressed
XM_015097989.2	2.281494361	0.013372331	Over expressed

XM_004008600.4	2.281379681	0.061571085	Over expressed
XM_004015420.3	2.280941267	0.03417323	Over expressed
XM_027974087.1	2.279822953	0.12197309	Over expressed
XM_027959151.1	2.275736575	1.02E-05	Over expressed
XM_027967913.1	2.275213093	0.115218779	Over expressed
XM_015103999.2	2.271102767	0.000258475	Over expressed
XM_027957148.1	2.271091842	0.000138193	Over expressed
XM_015104523.2	2.269745195	0.048171702	Over expressed
XM_015101063.2	2.268512393	0.001262701	Over expressed
XM_027969548.1	2.268149483	0.001305023	Over expressed
XM_004012518.4	2.265480504	0.071949208	Over expressed
XM_027976883.1	2.263607286	0.03195564	Over expressed
XM_027961436.1	2.256927571	0.00488154	Over expressed
NM_001252177.2	2.254209348	0.02030149	Over expressed
XM_004018960.4	2.251344623	0.103654233	Over expressed
XM_027972867.1	2.25102014	1.15E-06	Over expressed
XM_027956924.1	2.247232934	7.67E-05	Over expressed
XM_027968016.1	2.247057809	0.104824887	Over expressed
XR_001024822.3	2.243405686	0.005761197	Over expressed
XM_027957476.1	2.243136857	0.043359338	Over expressed
XR_001023556.3	2.240502277	0.068437798	Over expressed
XM_027959386.1	2.240294093	0.001952713	Over expressed
XM_004012823.3	2.238373836	0.020613284	Over expressed
XM_027960962.1	2.236791281	0.029824081	Over expressed
XM_027963743.1	2.235331069	0.045730072	Over expressed
XM_004023408.4	2.234850086	0.000430463	Over expressed
XM_027956949.1	2.233229274	0.00508936	Over expressed
XM_004005593.4	2.231020081	0.001694704	Over expressed
XM_027957492.1	2.228687745	0.022546466	Over expressed
XM_027969518.1	2.227853909	0.021464107	Over expressed
XM_027966080.1	2.227764261	0.000216684	Over expressed
XM_027974676.1	2.225001246	0.000725919	Over expressed
XM_027957563.1	2.224887685	0.060390695	Over expressed
XM_012102126.2	2.222433729	0.000176042	Over expressed
XM_027978897.1	2.221670586	0.001775269	Over expressed
XM_027968066.1	2.216190045	0.002474541	Over expressed
XM_004008602.4	2.215628202	0.060934593	Over expressed
XR_003589107.1	2.210401229	0.133324457	Over expressed
XM_012095332.3	2.208809247	0.140434525	Over expressed
XM_027956872.1	2.207021842	0.000113932	Over expressed
XM_027976870.1	2.204683132	0.000731727	Over expressed
XM_027978233.1	2.204682888	0.002923544	Over expressed
XM_015095733.2	2.199070898	1.08E-06	Over expressed
XR_003589889.1	2.19888506	0.063617621	Over expressed

XM_027974080.1	2.198631647	0.000632757	Over expressed
XM_012104345.3	2.197666116	0.000262429	Over expressed
XM_027959506.1	2.197447098	7.45E-07	Over expressed
XM_027965758.1	2.196500879	0.054921606	Over expressed
XM_027966003.1	2.193065447	0.105513473	Over expressed
XM_027957597.1	2.192497734	0.007275229	Over expressed
NM_001009429.1	2.187474182	0.142280067	Over expressed
XM_004016051.4	2.187287417	0.142393907	Over expressed
XM_027965629.1	2.186451728	0.013262645	Over expressed
XM_027956558.1	2.186143017	0.000521269	Over expressed
XM_027959797.1	2.183898746	0.012421174	Over expressed
XR_003590597.1	2.182956806	0.024242344	Over expressed
XM_027977200.1	2.180709551	0.012386965	Over expressed
XR_001030067.3	2.180300819	0.081834057	Over expressed
XM_027958855.1	2.180017364	0.130933705	Over expressed
XR_003586251.1	2.178704801	0.120153508	Over expressed
XM_015098906.2	2.171702671	0.011190743	Over expressed
XR_003590726.1	2.170934934	0.04821245	Over expressed
XM_004021001.3	2.167029897	0.030579045	Over expressed
XM_027978472.1	2.164997405	0.073745649	Over expressed
XM_027967668.1	2.164165205	0.110074099	Over expressed
XM_027970217.1	2.159051858	0.000130615	Over expressed
XM_027974271.1	2.158110586	0.000921468	Over expressed
XM_027959850.1	2.156302315	0.004642387	Over expressed
XM_027974179.1	2.152443033	0.137042147	Over expressed
NM_001134308.1	2.151727328	0.017406573	Over expressed
NM_001161869.1	2.149126625	0.001711775	Over expressed
XM_004017084.3	2.148350055	0.007131201	Over expressed
XM_027966112.1	2.144784901	0.000820085	Over expressed
XM_012158642.3	2.144203348	0.056963038	Over expressed
XM_027974993.1	2.141083501	0.116245729	Over expressed
XM_027971076.1	2.140437507	0.04270552	Over expressed
XM_027966106.1	2.136209087	0.009070639	Over expressed
NM_001035224.1	2.130387672	0.130265711	Over expressed
XM_012190732.3	2.128003722	0.045369055	Over expressed
XM_027969869.1	2.125483326	0.021481972	Over expressed
XM_027966600.1	2.125017079	0.017793199	Over expressed
XR_003589379.1	2.123746822	0.113299773	Over expressed
XM_027958093.1	2.122369043	0.093513871	Over expressed
XM_027968940.1	2.121860604	0.098453336	Over expressed
XM_015102172.2	2.119671025	0.001454395	Over expressed
XM_027973593.1	2.118923686	9.75E-06	Over expressed
XM_027975635.1	2.11647379	0.130540181	Over expressed
XM_027959959.1	2.115964804	0.081423408	Over expressed

XM_027966378.1	2.114121113	0.002680482	Over expressed
XM_015105278.2	2.1128905	0.009333354	Over expressed
XM_027974279.1	2.109866618	0.061431822	Over expressed
XM_027970191.1	2.109626138	0.011815879	Over expressed
XM_015100161.2	2.109211716	0.013166229	Over expressed
XM_027978340.1	2.107992658	0.080659293	Over expressed
XM_027961270.1	2.105164279	0.004630032	Over expressed
XM_027961465.1	2.100729877	0.030997269	Over expressed
XM_027957446.1	2.100359741	0.006836925	Over expressed
XM_027969338.1	2.10013364	1.20E-11	Over expressed
XM_027969610.1	2.098732917	0.000293771	Over expressed
XM_004018410.3	2.097733752	0.019206715	Over expressed
XM_027961144.1	2.095123982	0.009560257	Over expressed
XM_027977160.1	2.094337987	0.045256103	Over expressed
XM_004015082.4	2.092976257	0.011394995	Over expressed
XM_027959672.1	2.092760509	0.039893983	Over expressed
XM_012174798.3	2.092253529	0.007413789	Over expressed
NM_001009319.2	2.090403885	0.015994314	Over expressed
XM_004005644.4	2.089773215	0.004976659	Over expressed
XM_027969707.1	2.088867834	0.148620498	Over expressed
XM_027979802.1	2.088387888	0.09194277	Over expressed
XM_004001806.4	2.086665259	1.98E-05	Over expressed
XM_027969367.1	2.082940741	1.78E-14	Over expressed
XM_012103924.2	2.082839368	0.000924237	Over expressed
XM_027974212.1	2.080896055	7.55E-07	Over expressed
XM_027956943.1	2.078714915	0.079710872	Over expressed
XM_004006376.4	2.075711198	0.000924016	Over expressed
XM_027974190.1	2.074059902	0.104618029	Over expressed
XM_027959593.1	2.073927308	0.001559245	Over expressed
XM_004019615.4	2.073535323	0.000996364	Over expressed
XM_027965225.1	2.073180772	0.007163966	Over expressed
XM_004005271.3	2.071571171	0.027480359	Over expressed
XM_027977340.1	2.069318718	0.003835012	Over expressed
NM_001162552.1	2.067924914	0.006130053	Over expressed
XM_004012486.3	2.065220024	0.004225515	Over expressed
XM_027971264.1	2.063496465	0.014711053	Over expressed
XM_027977447.1	2.06240144	0.063641717	Over expressed
XM_027975637.1	2.060840056	0.009671194	Over expressed
XM_004022127.4	2.060809586	0.041973645	Over expressed
XM_027961046.1	2.059968586	0.027637143	Over expressed
XM_027974025.1	2.05524645	8.15E-10	Over expressed
XM_027960288.1	2.050292263	0.035200575	Over expressed
NM_001105261.1	2.049412325	0.035485701	Over expressed
XR_003590639.1	2.048313589	0.027866223	Over expressed

XM_012102025.3	2.047779003	0.046917179	Over expressed
XM_004005098.4	2.047319436	0.085845743	Over expressed
XM_027978401.1	2.043112349	0.027192005	Over expressed
XM_012102192.3	2.039105032	0.000814822	Over expressed
XM_027961829.1	2.037073754	0.004594698	Over expressed
XM_004015465.4	2.036257044	0.051563883	Over expressed
XM_027973355.1	2.0362261	0.002922056	Over expressed
XM_027976121.1	2.032803774	0.030216242	Over expressed
XM_004020924.3	2.031524836	0.05070529	Over expressed
XM_004008511.3	2.02951902	0.001406393	Over expressed
XR_003590770.1	2.029350165	0.026699482	Over expressed
XM_027975042.1	2.027466524	0.01556651	Over expressed
XM_027961472.1	2.026533446	0.023350651	Over expressed
NM_001308574.1	2.026041832	0.027366442	Over expressed
XM_027969284.1	2.025830912	0.008234738	Over expressed
XM_004014614.4	2.025482227	0.121259282	Over expressed
XM_004008468.4	2.023178919	0.022602338	Over expressed
XM_015100063.2	2.021891828	0.070747668	Over expressed
XM_027980283.1	2.017374085	0.001689254	Over expressed
XM_004006304.4	2.017270393	5.88E-06	Over expressed
XM_004010350.4	2.016887457	0.005199432	Over expressed
XR_003590125.1	2.015944362	0.10202281	Over expressed
XM_027974316.1	2.011331869	0.004828293	Over expressed
XM_004008775.4	2.010090302	0.002853833	Over expressed
NM_001012341.1	2.006206177	0.015738733	Over expressed
XM_012103012.3	2.006046224	0.01380001	Over expressed
XM_027974978.1	2.005544156	0.055677671	Over expressed
XM_027970396.1	2.003410689	0.024259214	Over expressed
XM_027979276.1	2.000945713	0.096559574	Over expressed
XM_012183465.2	2.00010099	0.012150769	Over expressed
XM_027959471.1	1.999728962	0.0303893	Over expressed
XM_027967308.1	1.998977907	0.004119139	Over expressed
XM_027977535.1	1.997794907	0.000782958	Over expressed
XM_027961828.1	1.996114737	0.001952713	Over expressed
XM_027959152.1	1.99443362	0.000274553	Over expressed
XM_027956706.1	1.990771994	0.056591949	Over expressed
XM_027957868.1	1.990115418	0.072405437	Over expressed
XM_027961208.1	1.990020156	0.028801284	Over expressed
XR_003586475.1	1.988892215	0.018304547	Over expressed
XM_027963297.1	1.987036757	0.014921056	Over expressed
XM_027961874.1	1.98682875	0.044142776	Over expressed
XM_027978839.1	1.98603889	0.071936565	Over expressed
XM_004008427.4	1.984313897	0.000413203	Over expressed
XM_012111094.3	1.983688309	0.015017327	Over expressed

XM_012169242.2	1.982215281	0.013002218	Over expressed
XM_027958660.1	1.980976248	0.006077065	Over expressed
XR_003588546.1	1.979706154	0.005249634	Over expressed
XM_027956445.1	1.9790844	0.000598911	Over expressed
XM_027960009.1	1.977189397	0.031146204	Over expressed
XM_004022778.3	1.976028424	0.001136631	Over expressed
XM_027961272.1	1.974968535	0.00295007	Over expressed
XM_004013065.4	1.972978552	0.028743102	Over expressed
XM_027978234.1	1.971259165	0.008864064	Over expressed
XM_012169237.3	1.971091026	0.004848344	Over expressed
XM_027956916.1	1.969046804	0.008504815	Over expressed
XM_015095576.2	1.967228165	0.040447863	Over expressed
XM_027967225.1	1.966680719	0.068243233	Over expressed
XM_027966627.1	1.963896074	0.057233625	Over expressed
XM_027966310.1	1.961106917	0.006326504	Over expressed
XM_004023592.4	1.959293269	0.073150133	Over expressed
XM_027976238.1	1.958769218	0.098471359	Over expressed
XM_027976335.1	1.957592476	0.083622691	Over expressed
XM_015101737.2	1.95736534	0.001205496	Over expressed
XM_027975638.1	1.955178886	0.011386162	Over expressed
XM_027959998.1	1.953032921	0.123593076	Over expressed
XM_012102093.2	1.952981761	0.051021572	Over expressed
XM_027979041.1	1.951756791	0.066811816	Over expressed
XM_012117934.3	1.951018952	0.047825491	Over expressed
XM_027966402.1	1.950335377	0.009045651	Over expressed
XM_027974127.1	1.950299718	0.010209275	Over expressed
XM_004008760.3	1.947905865	0.033993234	Over expressed
XM_027957787.1	1.947527018	5.29E-06	Over expressed
XM_004019672.4	1.946785038	0.005677224	Over expressed
XM_004005870.4	1.946652679	0.005152304	Over expressed
XM_027959273.1	1.945131817	0.001424413	Over expressed
NM_001009287.1	1.944855209	0.143650048	Over expressed
XM_027962802.1	1.943515602	0.02153044	Over expressed
XM_004017842.4	1.943131439	0.026656617	Over expressed
XM_015102223.2	1.943011304	0.00030712	Over expressed
XM_004001759.3	1.942410156	0.003030019	Over expressed
XM_004017737.3	1.942213425	0.002315763	Over expressed
XM_027961445.1	1.942182808	0.052342651	Over expressed
XM_027963524.1	1.94168303	0.145118602	Over expressed
XM_027957880.1	1.941542817	0.028011135	Over expressed
XM_004004394.4	1.941260689	0.02470076	Over expressed
XM_004007051.3	1.937613986	0.000369034	Over expressed
XM_027969397.1	1.936821463	0.001694704	Over expressed
XM_027961484.1	1.934403889	0.079988329	Over expressed

XM_004021776.4	1.934201442	2.74E-15	Over expressed
XM_004003094.4	1.934166045	0.018061258	Over expressed
XM_027960013.1	1.931937203	0.001134756	Over expressed
XM_027961332.1	1.931898814	0.045290788	Over expressed
XM_027973792.1	1.928397069	0.027039937	Over expressed
XM_004022254.4	1.928360702	0.001977086	Over expressed
XM_027957870.1	1.925110599	0.126095431	Over expressed
XM_027961373.1	1.922753946	0.01953568	Over expressed
XM_027968279.1	1.922556731	0.001933013	Over expressed
XM_027959928.1	1.922313201	0.00295007	Over expressed
XM_027976935.1	1.921433162	0.034011567	Over expressed
XM_027970171.1	1.920769427	5.67E-06	Over expressed
XM_012188069.3	1.920486581	0.035795462	Over expressed
XM_027964040.1	1.920075532	6.83E-07	Over expressed
XM_027976724.1	1.918066339	0.052211094	Over expressed
XM_004002292.4	1.916537717	0.149734613	Over expressed
XM_012175625.3	1.915160704	0.026396168	Over expressed
XM_015102889.2	1.910971048	5.70E-05	Over expressed
XM_004015685.4	1.908604667	0.005349469	Over expressed
XR_003588756.1	1.90813719	0.134808642	Over expressed
XM_027965744.1	1.907793751	0.00338515	Over expressed
XM_027958611.1	1.907492046	0.054640495	Over expressed
XM_027959994.1	1.907481785	0.117301299	Over expressed
XR_003590614.1	1.907160544	0.022452826	Over expressed
XM_027967221.1	1.907112263	0.006025018	Over expressed
XM_004023656.4	1.906858751	0.015738733	Over expressed
XM_027962060.1	1.904571093	0.008616488	Over expressed
XM_027969569.1	1.902869796	0.015344598	Over expressed
XM_015097618.2	1.900175147	0.116970127	Over expressed
XM_015103179.2	1.899369886	0.002558817	Over expressed
XM_027960677.1	1.895468336	0.001274938	Over expressed
XM_027963314.1	1.895440427	0.005643499	Over expressed
XM_027973168.1	1.895276769	0.021020478	Over expressed
XM_027957907.1	1.894601571	0.00012736	Over expressed
XM_015103984.2	1.892241388	0.029089452	Over expressed
XM_004014961.4	1.891580581	1.21E-05	Over expressed
XM_012158304.2	1.891224924	0.005339784	Over expressed
XM_027969913.1	1.889286124	0.075015492	Over expressed
XM_027956914.1	1.888024549	0.021448748	Over expressed
XM_012104341.3	1.888023414	0.003243995	Over expressed
XM_012190632.3	1.887349265	0.018156716	Over expressed
XM_027978940.1	1.886662288	0.001372545	Over expressed
XM_004005617.4	1.885364422	0.016899885	Over expressed
XM_027969312.1	1.885212556	0.101580157	Over expressed

XM_027978545.1	1.884319751	0.008805434	Over expressed
XM_015094112.2	1.883525883	0.041056259	Over expressed
XM_004012999.4	1.88222733	0.101580157	Over expressed
XM_027969408.1	1.881841983	0.114734629	Over expressed
XM_012187713.2	1.881399491	0.069323597	Over expressed
XM_027961645.1	1.88131024	2.53E-06	Over expressed
XM_027966364.1	1.880510814	4.29E-05	Over expressed
XR_003585754.1	1.880352854	0.008681624	Over expressed
XM_027969187.1	1.879867567	0.015923826	Over expressed
XM_027966541.1	1.87937331	0.000109708	Over expressed
XM_027957493.1	1.87804595	0.090061595	Over expressed
XM_004019706.4	1.87790001	0.005643499	Over expressed
XM_015100495.2	1.877719073	0.000268404	Over expressed
XM_004011604.4	1.8762995	0.000272931	Over expressed
XM_012150244.3	1.87550798	0.040138578	Over expressed
XM_015096865.2	1.875089394	0.017875212	Over expressed
XM_027966108.1	1.874070552	0.001981158	Over expressed
XM_027958153.1	1.873666654	0.118562687	Over expressed
XM_004010807.4	1.873220108	0.124196021	Over expressed
XM_027978821.1	1.873086769	3.87E-10	Over expressed
XM_004018994.4	1.872271075	0.005677224	Over expressed
XM_004013783.3	1.871735569	0.009134949	Over expressed
XM_027974776.1	1.871650893	0.126641018	Over expressed
XR_003591235.1	1.871456614	0.114227342	Over expressed
XM_004017484.3	1.870001168	0.005420103	Over expressed
XM_027958322.1	1.868185888	0.085729313	Over expressed
XM_004011587.4	1.867603759	0.036012555	Over expressed
XM_004007001.4	1.864478318	0.007348466	Over expressed
XM_004020888.4	1.864326068	0.056711472	Over expressed
NM_001038013.1	1.862548334	0.00432305	Over expressed
XM_027971986.1	1.862327099	0.08601828	Over expressed
XM_027966225.1	1.861112216	0.00035315	Over expressed
XM_027973387.1	1.860722335	8.34E-05	Over expressed
XM_027961179.1	1.860570913	0.077087707	Over expressed
XM_027956884.1	1.859013686	0.040317966	Over expressed
XM_004006053.4	1.857507373	0.09134946	Over expressed
XM_027975731.1	1.856106281	0.105556895	Over expressed
XM_027958830.1	1.856040132	0.000708045	Over expressed
XM_004008585.4	1.855273952	0.056242652	Over expressed
XM_012106143.3	1.854533666	0.047872437	Over expressed
XM_004005869.3	1.85420657	0.000194252	Over expressed
XM_004003428.4	1.85204277	0.012825176	Over expressed
XM_027970132.1	1.850244478	0.093062156	Over expressed
XM_027978769.1	1.849917808	0.009043296	Over expressed

XM_027973388.1	1.849889036	0.00303335	Over expressed
XM_027968352.1	1.848690209	0.017875212	Over expressed
XM_004005577.3	1.848383692	0.120498265	Over expressed
XM_004011603.3	1.848366803	0.000648606	Over expressed
XM_027970131.1	1.847195108	1.03E-09	Over expressed
XM_004012682.4	1.847184771	0.040192347	Over expressed
XM_015098783.2	1.846987242	0.044439613	Over expressed
XM_027966081.1	1.846422375	0.068970619	Over expressed
XM_004010301.4	1.843292165	5.95E-26	Over expressed
XM_004002403.4	1.842031236	0.040905381	Over expressed
XM_027967941.1	1.840958119	0.095995136	Over expressed
XM_027970185.1	1.838257629	0.000175445	Over expressed
XM_015094093.2	1.836947463	0.011051851	Over expressed
XM_027974094.1	1.83582046	0.001525587	Over expressed
XM_027960478.1	1.835270408	0.017344171	Over expressed
XM_027961905.1	1.834969217	1.14E-05	Over expressed
XM_027958712.1	1.834518976	0.008701424	Over expressed
XM_012099136.3	1.831578853	0.012526807	Over expressed
XM_004020994.4	1.830972008	0.006553512	Over expressed
XM_027976987.1	1.830837977	0.008248762	Over expressed
XM_012100180.3	1.830778811	0.117347728	Over expressed
XM_004006724.4	1.829707159	0.124841266	Over expressed
XM_004008401.3	1.828309708	0.023162673	Over expressed
XM_027973401.1	1.826766633	0.010826965	Over expressed
XM_027978298.1	1.826701533	0.003404664	Over expressed
XM_027974841.1	1.825631276	0.01987145	Over expressed
XM_004021022.4	1.825537558	0.000468678	Over expressed
XM_027961264.1	1.825221122	0.008121416	Over expressed
XM_027968199.1	1.825030354	0.00109297	Over expressed
XR_003591878.1	1.82385134	0.033991724	Over expressed
XM_027978928.1	1.821743049	0.043806866	Over expressed
XM_027969219.1	1.821468715	0.011747675	Over expressed
XM_012190166.3	1.821394244	0.067518204	Over expressed
XM_004019653.4	1.81998788	0.043424052	Over expressed
XM_012106759.3	1.81824016	0.000212418	Over expressed
XM_004006965.4	1.816995815	0.040317383	Over expressed
XR_003586211.1	1.815130195	0.066419921	Over expressed
XM_027972122.1	1.815051403	0.007413789	Over expressed
XR_003590709.1	1.814959812	0.013090879	Over expressed
XM_027975010.1	1.814767658	0.025728453	Over expressed
XM_004003093.4	1.814250015	0.041508388	Over expressed
XM_004002602.4	1.8137327	0.031196313	Over expressed
XM_027978857.1	1.813727212	3.40E-07	Over expressed
XM_027976885.1	1.813128649	0.024887046	Over expressed

XM_004004943.4	1.81272476	0.023077164	Over expressed
XR_001020974.2	1.810393432	0.09519335	Over expressed
XM_027963526.1	1.81003653	0.125073826	Over expressed
NM_001009449.1	1.809194239	0.08771212	Over expressed
XM_027959786.1	1.809051132	0.051021572	Over expressed
XM_004017758.4	1.808395888	0.019442625	Over expressed
XM_027965993.1	1.80680741	4.22E-07	Over expressed
XM_027971361.1	1.805740193	0.055980039	Over expressed
XM_027961165.1	1.805161841	2.47E-08	Over expressed
XM_004007021.4	1.804123314	0.011082114	Over expressed
XM_004018535.4	1.803576328	0.084608164	Over expressed
XM_004008437.4	1.802174412	0.139019656	Over expressed
XM_027978395.1	1.800568704	0.091631433	Over expressed
XM_004014955.3	1.798155331	0.114734629	Over expressed
XM_004005579.3	1.795528412	0.006990559	Over expressed
XM_027957885.1	1.795044801	0.077415236	Over expressed
XM_015093944.2	1.794694945	0.023939488	Over expressed
XM_027974247.1	1.79148782	0.000419328	Over expressed
XM_027977444.1	1.791022862	0.009043296	Over expressed
XM_004015329.4	1.789810799	0.000604191	Over expressed
XM_027969443.1	1.789110293	0.001699137	Over expressed
XM_027959017.1	1.788365214	0.08353401	Over expressed
XM_004008548.4	1.786438554	0.133159195	Over expressed
XM_027977512.1	1.785629023	0.081729291	Over expressed
XM_004010566.4	1.785286527	0.090169349	Over expressed
XM_027974276.1	1.784142266	1.79E-05	Over expressed
XM_027974275.1	1.781737016	0.022773918	Over expressed
XM_027979478.1	1.779084276	0.003724018	Over expressed
XM_027975986.1	1.776710081	0.100241732	Over expressed
XM_012100994.3	1.775380739	0.002224336	Over expressed
XM_027978476.1	1.774982211	0.000652953	Over expressed
XM_027978453.1	1.774886284	0.05051446	Over expressed
XM_027974105.1	1.774848436	0.142567156	Over expressed
XM_027967526.1	1.773892611	0.067998257	Over expressed
XM_027979806.1	1.773511686	0.019881571	Over expressed
XM_004020145.3	1.773126055	0.02231269	Over expressed
XM_015101934.2	1.772912204	0.020798222	Over expressed
XM_027966737.1	1.770311196	0.002149353	Over expressed
XM_027978758.1	1.76996411	4.42E-08	Over expressed
XM_027974500.1	1.769810377	6.18E-06	Over expressed
XR_003589446.1	1.769759697	0.037180511	Over expressed
XM_027978594.1	1.769352949	0.000477849	Over expressed
XM_027966397.1	1.769169903	0.103520258	Over expressed
XM_012160346.3	1.769138897	0.044202863	Over expressed

XM_027960175.1	1.768294296	0.034864585	Over expressed
XM_012100278.3	1.76812468	0.006809358	Over expressed
XM_004017663.4	1.763693444	0.011352163	Over expressed
XM_004005872.4	1.763522063	0.006018776	Over expressed
XM_027959990.1	1.763214608	6.13E-08	Over expressed
XR_003591670.1	1.761364992	0.115627782	Over expressed
XM_012190698.2	1.7603224	0.059518391	Over expressed
NM_001142517.1	1.759422114	3.02E-11	Over expressed
XM_015093072.2	1.758704375	0.008681624	Over expressed
XM_004009292.3	1.756956437	0.089666148	Over expressed
XR_003588567.1	1.75683974	0.008791375	Over expressed
XM_027961413.1	1.755325054	0.007106993	Over expressed
XM_027978650.1	1.752323924	0.017073828	Over expressed
XM_012174157.2	1.751238493	0.117022896	Over expressed
XM_012177564.2	1.749863639	0.03317565	Over expressed
XM_004015187.4	1.748110991	0.068050639	Over expressed
XM_027966012.1	1.747669993	0.144023509	Over expressed
XM_004008784.3	1.746831052	0.075896173	Over expressed
XM_027961903.1	1.742545229	0.04415186	Over expressed
XM_027959020.1	1.742046478	0.080938909	Over expressed
XM_027973986.1	1.740893668	0.079710872	Over expressed
XM_027971848.1	1.740438705	0.108934881	Over expressed
XM_015092769.2	1.739890295	0.004577608	Over expressed
XM_004019677.4	1.739678192	0.002270708	Over expressed
XM_027967381.1	1.737945549	0.053579367	Over expressed
XM_012104077.3	1.737320331	0.030743544	Over expressed
XM_027970026.1	1.736561008	2.80E-10	Over expressed
XM_027977514.1	1.734267449	0.021508369	Over expressed
XM_027959376.1	1.733428214	0.10548702	Over expressed
XM_004002522.3	1.732658724	0.089161969	Over expressed
XM_027969434.1	1.730727822	0.012444279	Over expressed
NM_001205150.1	1.730177419	0.037188369	Over expressed
XM_027959367.1	1.730125613	0.011448767	Over expressed
XM_004019650.4	1.727024041	0.034128172	Over expressed
XM_027958665.1	1.726904875	0.000178742	Over expressed
XM_027961791.1	1.726500748	0.035935522	Over expressed
XM_027957006.1	1.726120065	0.051900231	Over expressed
XM_004018416.4	1.726047435	0.089666148	Over expressed
XM_027973919.1	1.72473719	0.026507839	Over expressed
XM_012185668.3	1.724689984	0.065347716	Over expressed
XM_027961789.1	1.723625604	0.008025361	Over expressed
XM_004018740.4	1.71910779	0.090169349	Over expressed
XM_004002608.4	1.71900361	0.105599617	Over expressed
XM_027978389.1	1.718415833	0.033228707	Over expressed

XM_012174651.2	1.717826543	0.016903023	Over expressed
XM_027969310.1	1.717590046	0.008426193	Over expressed
XM_012162156.3	1.716373681	0.046931213	Over expressed
XM_027966855.1	1.715168779	0.049923796	Over expressed
NM_001245974.1	1.715163003	0.065347716	Over expressed
XM_027969649.1	1.71448729	0.005199432	Over expressed
XM_015104226.2	1.714203791	0.01972331	Over expressed
XM_027959973.1	1.712552948	0.006025018	Over expressed
XM_027978121.1	1.712194868	0.016610802	Over expressed
XM_004015437.4	1.712188015	0.050209353	Over expressed
XR_001034198.3	1.711439502	0.017559004	Over expressed
XM_027971423.1	1.710705337	0.017073828	Over expressed
XM_027960758.1	1.709862805	0.094805354	Over expressed
XM_027958036.1	1.709469911	0.076557047	Over expressed
XM_012104520.3	1.708138501	0.14617597	Over expressed
NM_001139452.1	1.707966296	0.057852004	Over expressed
XM_012095885.3	1.705393379	0.000404514	Over expressed
XM_012187710.2	1.702778471	0.079997542	Over expressed
XM_004023490.4	1.702552649	0.063321285	Over expressed
XM_027971704.1	1.700771835	0.084035725	Over expressed
XM_027959847.1	1.700622373	0.006245894	Over expressed
XM_027957340.1	1.699382423	0.033449144	Over expressed
XM_027962961.1	1.699170844	3.94E-18	Over expressed
XM_004002580.4	1.698575475	0.015017327	Over expressed
XM_004008546.4	1.69756559	0.003621121	Over expressed
XM_027973438.1	1.695083911	0.021421875	Over expressed
XR_001034868.3	1.692036385	0.00215865	Over expressed
XM_004013080.4	1.691575219	0.071725236	Over expressed
XM_004020717.4	1.69058606	0.000953447	Over expressed
XM_012104387.2	1.690207723	0.007461413	Over expressed
XM_027966834.1	1.690203978	0.105556895	Over expressed
XM_012107522.2	1.68824198	0.015861546	Over expressed
XM_027960820.1	1.686502179	0.004348569	Over expressed
XM_027972279.1	1.682871492	0.021124401	Over expressed
XM_027971553.1	1.68103618	0.03509318	Over expressed
XM_004018850.4	1.680791813	0.027857065	Over expressed
XM_004006979.4	1.680090522	0.023535402	Over expressed
XM_027974439.1	1.678815549	0.001055054	Over expressed
XM_027977221.1	1.677485667	0.093765712	Over expressed
XM_027968254.1	1.676839025	0.030888534	Over expressed
XM_027973793.1	1.675620781	0.067344828	Over expressed
XM_015104222.2	1.674720086	0.018513262	Over expressed
XM_027969320.1	1.674425346	0.072458819	Over expressed
XM_027969446.1	1.673563674	0.001040944	Over expressed

XM_015098668.2	1.671516555	0.005349469	Over expressed
XM_027963523.1	1.670544986	0.042779189	Over expressed
XM_027956242.1	1.66981021	0.029102016	Over expressed
XM_012189344.3	1.668828711	0.021301653	Over expressed
XM_012099958.3	1.668064668	0.116127278	Over expressed
XM_027974048.1	1.667676342	3.66E-06	Over expressed
XM_027956939.1	1.667102527	0.030743544	Over expressed
XM_027975636.1	1.666457764	0.054004676	Over expressed
XM_004012669.3	1.665799102	0.026507839	Over expressed
XM_004008049.4	1.664871556	0.012505497	Over expressed
XM_012177581.2	1.664116399	0.10929768	Over expressed
XM_027966284.1	1.66020868	0.115757473	Over expressed
XM_027978396.1	1.659763861	0.127543618	Over expressed
XM_027971297.1	1.659348696	9.24E-08	Over expressed
XM_012183404.2	1.659136495	0.004834655	Over expressed
XM_027960479.1	1.659128758	0.008331256	Over expressed
XM_027966150.1	1.658757265	4.74E-05	Over expressed
XM_004017395.4	1.655640958	0.141812568	Over expressed
XR_003591756.1	1.654308646	0.049948168	Over expressed
XM_004006729.4	1.65394876	0.031196313	Over expressed
XM_004008576.4	1.653083915	6.25E-05	Over expressed
XM_027971211.1	1.652338557	0.094592043	Over expressed
XM_004020978.4	1.649930247	0.021565867	Over expressed
XM_027975631.1	1.649168206	0.018769702	Over expressed
XM_004020904.4	1.648644821	0.040192347	Over expressed
XM_012183007.2	1.648262378	0.00652663	Over expressed
XM_012174322.3	1.647887729	0.029800936	Over expressed
XM_004021571.3	1.647547135	0.032574721	Over expressed
XM_027968256.1	1.64695957	0.127677096	Over expressed
XM_027971016.1	1.644550285	0.017406573	Over expressed
XM_015094163.2	1.64434121	0.009954469	Over expressed
XM_015104240.2	1.644152613	0.000652631	Over expressed
XM_027968893.1	1.643721333	0.023996518	Over expressed
XM_012130401.3	1.643559109	0.037920102	Over expressed
XM_004007055.4	1.643054889	0.0016319	Over expressed
XM_015093696.2	1.642551658	0.014994054	Over expressed
XM_027963037.1	1.641727488	0.005492587	Over expressed
XM_004012580.4	1.639331208	0.084518502	Over expressed
XM_015100713.2	1.638111739	7.69E-05	Over expressed
XM_027968244.1	1.637638857	0.008155947	Over expressed
XR_003591376.1	1.63647184	0.009134949	Over expressed
XM_027969724.1	1.635905273	0.060794449	Over expressed
XR_003587283.1	1.635614588	0.04978737	Over expressed
XR_003588569.1	1.634300717	0.000990045	Over expressed

XM_012174649.2	1.633227882	0.13094989	Over expressed
XM_027971171.1	1.632049792	0.000110161	Over expressed
XM_004015111.4	1.631975422	0.142200853	Over expressed
XM_027979347.1	1.631056778	0.078081541	Over expressed
XM_027974139.1	1.629757934	0.031508716	Over expressed
XM_004002695.4	1.629544357	1.86E-07	Over expressed
XM_012183443.3	1.62899948	0.002634761	Over expressed
XM_027970028.1	1.627364442	0.001537661	Over expressed
XM_004004894.4	1.626506764	0.118757638	Over expressed
XM_027978526.1	1.625333111	0.054657193	Over expressed
XM_004015419.4	1.624596164	0.001980175	Over expressed
XM_004004265.4	1.623088643	0.068576147	Over expressed
XM_015100617.2	1.622528964	0.023409628	Over expressed
XM_027979805.1	1.622345924	0.007275229	Over expressed
XM_004022140.3	1.620270709	0.064733848	Over expressed
XM_027958117.1	1.618682897	0.08471501	Over expressed
XM_027969836.1	1.616724573	3.53E-06	Over expressed
XM_015096093.2	1.615332982	0.033309104	Over expressed
XM_027970239.1	1.613900437	0.000391364	Over expressed
XM_027977945.1	1.61279382	0.013657581	Over expressed
XM_012100093.2	1.611771824	4.43E-05	Over expressed
XM_012173775.2	1.611503858	0.08012409	Over expressed
XM_004023349.4	1.608660861	0.026896125	Over expressed
XM_015094243.2	1.608333564	0.037413702	Over expressed
XM_012175860.3	1.607964624	0.010557192	Over expressed
XM_027974097.1	1.606857217	0.086085084	Over expressed
XM_027977006.1	1.606468058	0.049027713	Over expressed
XM_027961469.1	1.605400878	0.064251997	Over expressed
XM_027967524.1	1.60347348	0.03760585	Over expressed
XM_027966433.1	1.602995129	0.111091235	Over expressed
XM_015098606.2	1.601046946	0.032193485	Over expressed
XM_027957539.1	1.600362674	0.049002159	Over expressed
XM_004002344.4	1.600236247	0.073745649	Over expressed
XM_004004921.3	1.599644556	9.23E-05	Over expressed
XM_004008556.3	1.598059662	0.134256419	Over expressed
XM_027970192.1	1.597983593	0.140400335	Over expressed
XM_027961638.1	1.596962707	0.027991076	Over expressed
XM_027967615.1	1.596829262	0.036723101	Over expressed
XM_027971267.1	1.5966963	0.098121422	Over expressed
XM_027956936.1	1.596099662	7.80E-06	Over expressed
XM_027961934.1	1.595416103	0.04269936	Over expressed
XM_004005559.4	1.595293071	0.000652866	Over expressed
XR_003588841.1	1.592940815	0.017569601	Over expressed
XM_015095865.2	1.590974175	0.024405786	Over expressed

XM_027975385.1	1.59067198	0.018297923	Over expressed
XM_004012769.3	1.590514025	0.044764845	Over expressed
XM_027967940.1	1.58918727	0.057400697	Over expressed
XM_004015386.4	1.588466102	0.104694174	Over expressed
XM_027966154.1	1.588403276	0.06819525	Over expressed
XM_004008575.4	1.587877331	0.024310909	Over expressed
XM_004018837.4	1.587054521	0.012910881	Over expressed
XM_004017738.3	1.586911302	0.013372331	Over expressed
XM_027963278.1	1.585590557	0.075116027	Over expressed
XM_015097580.2	1.584863338	0.011386162	Over expressed
XM_004020714.4	1.584789989	0.108390843	Over expressed
XM_027977141.1	1.582532688	0.010501455	Over expressed
XM_027979778.1	1.582267556	0.021482536	Over expressed
NM_001105263.1	1.581630728	0.112194594	Over expressed
XM_004022654.4	1.580817006	5.53E-08	Over expressed
XM_027976051.1	1.580641209	0.058804733	Over expressed
XM_004006836.4	1.580601341	0.046233285	Over expressed
XM_027974280.1	1.578188564	0.113782772	Over expressed
XM_027970127.1	1.575634535	3.11E-05	Over expressed
XR_003591239.1	1.575167625	0.094813745	Over expressed
XM_004019714.4	1.573708597	0.00348434	Over expressed
XM_027958779.1	1.573572436	0.000989973	Over expressed
XM_015098595.2	1.573045452	0.003884336	Over expressed
XM_004014164.4	1.572544352	0.005013366	Over expressed
XM_027962102.1	1.5703743	0.122519414	Over expressed
XM_004007014.4	1.569228857	0.010154235	Over expressed
XM_004014883.4	1.56892214	0.065004996	Over expressed
XM_027961895.1	1.567809587	0.023077164	Over expressed
XM_027972960.1	1.567641063	0.125007433	Over expressed
XM_027959130.1	1.567526848	0.025548037	Over expressed
XM_027957639.1	1.566815467	0.018152862	Over expressed
XM_004018227.4	1.566325907	0.000643223	Over expressed
XM_004006935.4	1.564699329	0.04452835	Over expressed
XM_027975856.1	1.563223364	0.126219877	Over expressed
XM_027957448.1	1.562634319	0.128230622	Over expressed
XM_027978069.1	1.561878812	0.091811836	Over expressed
XM_027970576.1	1.560757648	0.001559245	Over expressed
XM_027978419.1	1.558524194	0.076341965	Over expressed
XM_015102653.2	1.558128068	0.037190462	Over expressed
XM_027957862.1	1.557059434	0.007230763	Over expressed
XM_015098950.2	1.554971618	0.133956634	Over expressed
XM_027962103.1	1.553239967	0.1063414	Over expressed
XM_027978765.1	1.551802789	0.074307569	Over expressed
XM_027970129.1	1.54958956	0.137884717	Over expressed

XM_027959466.1	1.549530904	0.000364108	Over expressed
XM_027968127.1	1.548473179	0.025050032	Over expressed
XM_027962018.1	1.547820345	0.055677671	Over expressed
XM_027966891.1	1.546154269	0.011394995	Over expressed
XM_004013784.4	1.543090688	4.94E-05	Over expressed
XM_027958387.1	1.542643787	0.086774575	Over expressed
XM_027967017.1	1.542172091	0.068050639	Over expressed
XM_027957192.1	1.539971389	0.11980626	Over expressed
XM_027978859.1	1.538355488	0.01204115	Over expressed
XM_027961148.1	1.537523606	0.005429258	Over expressed
XM_004004374.4	1.535753628	0.071849297	Over expressed
NM_001134303.1	1.535381661	0.057877864	Over expressed
XM_015098887.2	1.53350247	0.063876801	Over expressed
XM_004008478.3	1.532067599	0.03303547	Over expressed
XM_027978531.1	1.531223985	5.89E-06	Over expressed
XM_027956642.1	1.528839492	0.109608148	Over expressed
XM_027963034.1	1.527268483	0.048195485	Over expressed
XM_027961198.1	1.52613878	0.114942497	Over expressed
XM_012100685.2	1.524749896	0.000725919	Over expressed
XM_027976832.1	1.524079059	0.020793974	Over expressed
XR_003590450.1	1.523585628	0.125437259	Over expressed
XM_027958685.1	1.522962464	0.113291459	Over expressed
XM_027970532.1	1.522887186	0.048743718	Over expressed
XM_004012825.4	1.522370522	0.033995756	Over expressed
XM_027972446.1	1.522188005	0.002562297	Over expressed
XM_027960852.1	1.521652653	0.038314995	Over expressed
XM_027961926.1	1.521588194	0.009155017	Over expressed
XM_027977627.1	1.521423693	0.107697796	Over expressed
XM_004008988.4	1.521069551	0.054921606	Over expressed
XM_004020696.3	1.519558372	4.58E-05	Over expressed
XM_027964963.1	1.51915114	0.102272972	Over expressed
XM_027959395.1	1.518997315	0.038243343	Over expressed
XM_027958015.1	1.518810741	0.081837807	Over expressed
XM_027961490.1	1.518529221	6.83E-07	Over expressed
XM_004017783.4	1.517383671	0.083679115	Over expressed
XM_027969020.1	1.517272237	0.023325618	Over expressed
XM_027971345.1	1.516600629	0.042176471	Over expressed
XM_015101978.2	1.515387328	0.13014285	Over expressed
XM_004021069.4	1.512137115	0.008252168	Over expressed
XR_003589942.1	1.511960131	0.12460053	Over expressed
XM_012099274.2	1.511185737	6.14E-19	Over expressed
XM_004019603.4	1.510673972	4.03E-05	Over expressed
XM_004015318.4	1.510248174	0.110224638	Over expressed
XM_027957723.1	1.508423329	0.044142627	Over expressed

XM_027967358.1	1.508294653	0.13014285	Over expressed
XM_027962056.1	1.508076541	0.07956239	Over expressed
XM_012099936.3	1.507502653	0.132221696	Over expressed
XM_012100964.2	1.505504814	0.069012514	Over expressed
XM_027961356.1	1.505208443	0.010012584	Over expressed
XM_027966182.1	1.505144771	0.013856063	Over expressed
XM_027961751.1	1.504712876	0.024617121	Over expressed
XM_012101646.3	1.504585354	0.023137406	Over expressed
XM_027961610.1	1.504521537	0.002921991	Over expressed
XR_003591587.1	1.504174913	0.090703348	Over expressed
XM_004020897.4	1.503846271	0.006368803	Over expressed
XM_027966738.1	1.502741378	0.040905381	Over expressed
XM_012120828.3	1.502575634	0.001851213	Over expressed
XM_027970313.1	1.501741584	0.039275584	Over expressed
XM_004007057.4	1.500470328	0.00752197	Over expressed
XM_027967403.1	1.499996126	0.037172299	Over expressed
XR_003587633.1	1.498545069	0.000248305	Over expressed
XR_003591300.1	1.498505059	0.038453148	Over expressed
XM_027978544.1	1.4984481	0.020480763	Over expressed
XM_027968699.1	1.496042456	0.009270789	Over expressed
XM_027969448.1	1.495812908	0.027450496	Over expressed
XM_004019694.4	1.495021867	0.021828034	Over expressed
XM_004021023.4	1.493620663	0.012639273	Over expressed
XM_027961582.1	1.493531812	0.028883374	Over expressed
XM_004018927.4	1.493171814	0.009684676	Over expressed
XM_004007054.4	1.492866177	0.135976784	Over expressed
XM_004012926.3	1.492583577	1.69E-09	Over expressed
XM_027963691.1	1.491138892	0.002062358	Over expressed
XM_027965673.1	1.491109195	0.03082761	Over expressed
XM_015091922.2	1.490912589	0.037683243	Over expressed
XM_004015648.4	1.490362175	0.000362879	Over expressed
XM_027968065.1	1.489463475	0.012505497	Over expressed
XM_027970150.1	1.488125393	0.047973402	Over expressed
XM_004012666.4	1.487847406	0.017317709	Over expressed
XM_004013075.3	1.486920949	0.002053308	Over expressed
XM_004006603.4	1.483513442	0.0897164	Over expressed
XM_004015366.3	1.482408626	0.001049855	Over expressed
XM_004010530.4	1.481944951	0.069598092	Over expressed
XM_004012985.4	1.481772036	0.004577608	Over expressed
XM_027957876.1	1.480962307	0.002642975	Over expressed
XM_027966067.1	1.479134563	0.036353636	Over expressed
XM_027961460.1	1.478979993	0.000734898	Over expressed
XM_012100918.3	1.476663494	0.003282492	Over expressed
XM_027965218.1	1.476402531	0.00366982	Over expressed

XM_027973336.1	1.476368484	0.001452677	Over expressed
XR_003591460.1	1.47365263	0.08218688	Over expressed
XR_001436363.2	1.472930581	0.11401293	Over expressed
XM_027963279.1	1.471521684	0.004079251	Over expressed
XM_015099477.2	1.469798863	0.002524616	Over expressed
XM_012182049.2	1.468710343	0.073745649	Over expressed
XM_015099409.2	1.468565895	0.086422197	Over expressed
XM_004006170.4	1.468217508	0.001159486	Over expressed
XM_004020701.4	1.467899072	0.055676361	Over expressed
XM_004012798.4	1.466946989	0.000927343	Over expressed
XM_027978469.1	1.46494419	0.023405321	Over expressed
XM_012105786.3	1.464606989	0.009134949	Over expressed
XM_027978291.1	1.462217336	1.21E-06	Over expressed
XR_003591061.1	1.460510484	0.000304676	Over expressed
XM_004002480.4	1.459651827	0.120599782	Over expressed
XM_027969415.1	1.458139905	0.000632757	Over expressed
XM_027965709.1	1.456569954	0.043359338	Over expressed
XM_027978777.1	1.4547977	0.009910192	Over expressed
XM_004015372.3	1.448817118	0.115757473	Over expressed
XM_027957668.1	1.448753743	0.031713873	Over expressed
XM_027977435.1	1.447387221	0.101661628	Over expressed
XM_004012763.4	1.446874567	2.09E-05	Over expressed
XM_027965207.1	1.44530304	0.055671546	Over expressed
XM_015098669.2	1.445191865	0.02145162	Over expressed
XM_004006155.3	1.444633235	0.084223096	Over expressed
XM_004015605.4	1.444147243	1.19E-06	Over expressed
XM_004016101.4	1.442977876	0.005908167	Over expressed
XM_027977780.1	1.442506861	0.08686471	Over expressed
XM_015095152.2	1.440253669	0.014706451	Over expressed
XM_027961255.1	1.440055072	0.038453148	Over expressed
XM_012174323.3	1.439858402	0.056767097	Over expressed
XM_027966844.1	1.438930421	0.029099651	Over expressed
XM_027978485.1	1.438826986	0.051900231	Over expressed
XM_027961752.1	1.438076852	6.77E-05	Over expressed
XM_004014607.4	1.43687785	0.120445737	Over expressed
XM_027960093.1	1.436767586	0.036157469	Over expressed
XM_027975040.1	1.436634356	0.132107442	Over expressed
XM_027978426.1	1.436614435	0.000589551	Over expressed
XM_027959594.1	1.436006252	0.026311405	Over expressed
XM_012188066.3	1.435289599	0.011678027	Over expressed
XM_027960724.1	1.43464443	0.010154235	Over expressed
XM_012174780.3	1.434373982	0.010209275	Over expressed
XM_012100843.3	1.433382165	0.08218688	Over expressed
XM_004020088.4	1.431866074	0.075520292	Over expressed

XR_003589751.1	1.430277265	0.056187657	Over expressed
XM_027960178.1	1.429943317	0.033188918	Over expressed
XM_027975909.1	1.429671148	0.081729291	Over expressed
XM_027978310.1	1.426627448	0.127903557	Over expressed
XM_015098945.2	1.426086745	0.040905381	Over expressed
XM_027962912.1	1.424785648	0.082637513	Over expressed
XM_027962781.1	1.423858903	1.91E-10	Over expressed
XM_027961182.1	1.423853061	0.019272011	Over expressed
XR_003587166.1	1.423515016	0.073591388	Over expressed
XM_004014247.4	1.422977697	0.026896125	Over expressed
XM_027964882.1	1.422568722	0.018614384	Over expressed
XM_012098122.3	1.417705663	0.00372592	Over expressed
XM_027960208.1	1.417004193	0.056950774	Over expressed
XM_027969781.1	1.41696949	0.048420843	Over expressed
XM_027974300.1	1.414720871	0.008183892	Over expressed
XM_027974613.1	1.413039408	0.047825491	Over expressed
XM_027978841.1	1.411920298	0.000551225	Over expressed
XM_027978947.1	1.411406869	0.031672061	Over expressed
XM_004015353.4	1.411035036	0.081599705	Over expressed
XM_027971271.1	1.409479152	0.001195832	Over expressed
XM_027958691.1	1.409337872	0.109397317	Over expressed
XM_027961816.1	1.408296594	1.45E-05	Over expressed
XM_027961830.1	1.40736146	0.032762505	Over expressed
XM_004014508.4	1.407258089	0.052559636	Over expressed
XM_012178062.2	1.407130058	0.01120223	Over expressed
XM_004015466.4	1.406395213	0.044764845	Over expressed
NM_001161734.1	1.405830054	0.033991724	Over expressed
XM_027972124.1	1.405551827	0.013296041	Over expressed
XM_015101666.2	1.403263581	0.068994586	Over expressed
XM_004005911.4	1.402873109	0.021436219	Over expressed
XM_027957323.1	1.402062844	0.098471359	Over expressed
XM_027974252.1	1.398901817	0.006440947	Over expressed
XM_027962067.1	1.398819827	4.61E-05	Over expressed
XM_004004295.3	1.398627544	0.024259214	Over expressed
XM_027960972.1	1.396565965	0.001033208	Over expressed
XM_027977786.1	1.396311428	0.11898354	Over expressed
XR_003590594.1	1.395309677	0.008213942	Over expressed
XM_027966319.1	1.394390736	0.096682644	Over expressed
XM_027964508.1	1.392297993	0.000184248	Over expressed
XM_027968713.1	1.39195174	0.023792089	Over expressed
XM_027970317.1	1.390749837	0.005035259	Over expressed
XM_027969889.1	1.388855566	0.097314195	Over expressed
XM_027959638.1	1.388333107	0.140216823	Over expressed
XM_012169297.3	1.387719956	0.001527043	Over expressed

XM_027959302.1	1.385368358	0.060289688	Over expressed
XR_003586407.1	1.384039844	0.114276131	Over expressed
XM_027977161.1	1.38371882	0.115757473	Over expressed
XR_003590713.1	1.382762986	0.057987599	Over expressed
XM_015100465.2	1.382729564	0.058259086	Over expressed
XM_004013842.3	1.382613092	0.009658024	Over expressed
XM_027971279.1	1.382230222	0.000979204	Over expressed
XM_027976077.1	1.381718502	0.034028849	Over expressed
XR_003585569.1	1.380976784	0.029824081	Over expressed
XM_027965923.1	1.379651869	0.087691539	Over expressed
XM_027966253.1	1.379208108	0.01133288	Over expressed
XM_004004069.4	1.379087378	0.149437976	Over expressed
XM_015096058.2	1.377802911	0.099181007	Over expressed
XM_004020915.4	1.377574712	0.021289469	Over expressed
XM_027957166.1	1.376794312	0.069739752	Over expressed
XM_004007027.3	1.376556382	0.004243687	Over expressed
XM_027979834.1	1.376448419	0.006965488	Over expressed
XM_027956406.1	1.375052216	0.003330493	Over expressed
XM_027973344.1	1.374732338	0.004724994	Over expressed
XM_004012943.4	1.374038076	0.029824081	Over expressed
XM_012158598.2	1.373282721	0.026507839	Over expressed
XM_027959715.1	1.371661771	0.14014596	Over expressed
XM_004020126.4	1.369767921	0.002840247	Over expressed
XM_015099492.2	1.369281707	0.036462063	Over expressed
NM_001126344.1	1.369233058	0.025050032	Over expressed
XM_027966174.1	1.368418295	3.29E-06	Over expressed
XM_015103188.2	1.367864633	0.029824081	Over expressed
XM_027968290.1	1.36766912	0.019989356	Over expressed
XM_027966739.1	1.366575546	0.00090434	Over expressed
XM_004012790.4	1.366317292	0.094373585	Over expressed
XM_004002593.4	1.366248571	0.007970897	Over expressed
XM_004008974.2	1.366146631	0.071123693	Over expressed
XM_027957872.1	1.365798233	0.063812555	Over expressed
XM_027970834.1	1.365571553	0.04962176	Over expressed
XM_027967982.1	1.364200799	0.0090353	Over expressed
XM_004021798.4	1.361639158	0.001087475	Over expressed
XM_004023614.4	1.360794697	0.040249175	Over expressed
NM_001166181.1	1.360694139	0.05740509	Over expressed
XM_004010321.4	1.357896367	0.010914748	Over expressed
XM_015103636.2	1.357812577	0.07218715	Over expressed
XM_027961287.1	1.357286069	0.114734629	Over expressed
XM_027968250.1	1.356651887	0.109989214	Over expressed
XM_027961234.1	1.356173328	0.038453148	Over expressed
XM_027974052.1	1.355476821	0.007275229	Over expressed

XM_015101781.2	1.35498993	0.034864585	Over expressed
XM_015093687.2	1.354461634	0.136981256	Over expressed
XM_027959526.1	1.354419115	0.038565798	Over expressed
XM_004022513.4	1.35349905	0.008681624	Over expressed
XM_027969837.1	1.353477456	0.029126185	Over expressed
XM_012096635.3	1.353160621	0.107697796	Over expressed
XM_027974994.1	1.352976933	0.084035725	Over expressed
XM_027961162.1	1.351711531	0.010809775	Over expressed
XR_001043959.3	1.351235737	0.122511419	Over expressed
XM_004022084.4	1.350342253	0.00209685	Over expressed
XM_027962804.1	1.349605838	0.023325618	Over expressed
XM_027959257.1	1.349421113	0.122387102	Over expressed
XM_012183038.3	1.349399277	0.041498006	Over expressed
XM_027960505.1	1.348715429	0.001136631	Over expressed
XM_027959583.1	1.348667206	0.039560416	Over expressed
XM_027961297.1	1.348353963	0.005581919	Over expressed
XM_027958037.1	1.344373766	0.027487238	Over expressed
XM_027958440.1	1.344273763	0.011087854	Over expressed
XM_004018464.4	1.344094158	0.048219576	Over expressed
XR_001041966.3	1.342989459	0.140216823	Over expressed
XM_027961052.1	1.342472737	0.008864064	Over expressed
XM_015099215.2	1.339336462	0.022395235	Over expressed
XM_027975141.1	1.338896871	1.26E-11	Over expressed
XM_027965459.1	1.338315483	0.013372331	Over expressed
XM_027962068.1	1.337830827	0.08471501	Over expressed
XM_004015467.4	1.337095759	0.018769702	Over expressed
XM_027972754.1	1.336357478	0.012264715	Over expressed
XM_012104119.3	1.335915326	0.129988258	Over expressed
XM_027958658.1	1.335459735	0.004630032	Over expressed
XM_027977612.1	1.335443956	0.010724842	Over expressed
XM_027977463.1	1.334049068	0.063675194	Over expressed
XM_027969694.1	1.333391138	0.033453964	Over expressed
XM_004014361.3	1.333252888	0.047872437	Over expressed
NM_001131030.1	1.331780678	0.074207643	Over expressed
XM_004015256.4	1.331691492	0.000453158	Over expressed
XM_027974287.1	1.331115227	0.115720612	Over expressed
XM_004004132.4	1.330106592	0.076791544	Over expressed
NM_001009786.2	1.328656351	0.029412644	Over expressed
XM_027972933.1	1.328493546	0.149437976	Over expressed
XM_004022237.3	1.326270247	0.094592043	Over expressed
XM_004014563.4	1.326034014	0.036012555	Over expressed
XM_027960123.1	1.324605438	0.097778363	Over expressed
XM_015104208.2	1.324259603	0.000128364	Over expressed
XM_027961788.1	1.324090476	0.097335284	Over expressed

XM_012173607.3	1.321009735	0.028161111	Over expressed
XM_027961738.1	1.320388753	0.130110015	Over expressed
XM_004018476.4	1.319615053	0.028011135	Over expressed
XR_003588455.1	1.319302021	0.069913687	Over expressed
XM_004001992.3	1.317761092	0.10561358	Over expressed
XM_027977707.1	1.317082145	0.012526807	Over expressed
XM_004014461.4	1.316096528	0.023894404	Over expressed
XM_027960091.1	1.314905368	0.04821245	Over expressed
XM_004016454.4	1.31440834	0.001276696	Over expressed
XM_004020271.4	1.314362334	0.075594242	Over expressed
XR_001435461.2	1.314350202	0.113278846	Over expressed
XM_012130509.3	1.313660944	0.002342774	Over expressed
XM_027977760.1	1.313559008	0.054668764	Over expressed
XM_027969571.1	1.311518858	0.028538033	Over expressed
XM_012175492.2	1.31071134	0.142567156	Over expressed
XM_012190518.2	1.310153508	0.044162281	Over expressed
XM_027959704.1	1.309649512	0.132291047	Over expressed
XM_027977425.1	1.309536582	0.024259214	Over expressed
XM_027974040.1	1.308903248	0.062221978	Over expressed
XM_012100083.2	1.307726865	0.129315715	Over expressed
XM_027958663.1	1.305359711	0.01795119	Over expressed
XM_027958657.1	1.304470119	0.000515035	Over expressed
XM_027962323.1	1.303579809	0.083747551	Over expressed
XM_027976777.1	1.303204237	0.142676804	Over expressed
XM_004019902.4	1.302938279	0.104439427	Over expressed
XM_027973349.1	1.302467405	0.001487448	Over expressed
XM_012098681.3	1.302061605	0.000921468	Over expressed
XM_004006719.4	1.300106878	0.001105824	Over expressed
XM_027975751.1	1.295773092	0.076791544	Over expressed
XM_027969373.1	1.29530512	0.061730028	Over expressed
NM_001126353.1	1.293655556	0.03650862	Over expressed
XM_027958723.1	1.293036168	0.127620913	Over expressed
XM_027959527.1	1.292872385	0.007961153	Over expressed
XM_015095885.2	1.292730381	9.17E-08	Over expressed
XM_004022128.4	1.290498568	0.097178042	Over expressed
XM_027976978.1	1.290212089	0.139681086	Over expressed
XM_012101347.3	1.288642264	0.053799132	Over expressed
XM_012187324.2	1.287669436	0.120737778	Over expressed
XM_027957663.1	1.287356902	0.101580157	Over expressed
XM_004007040.3	1.28711924	0.004179779	Over expressed
XM_015101747.2	1.286729294	0.01681293	Over expressed
XM_027956913.1	1.286078871	0.000686503	Over expressed
XM_012182917.2	1.284453029	0.005349469	Over expressed
XM_027957709.1	1.283969098	0.012707724	Over expressed

XM_027971918.1	1.282822002	0.023405321	Over expressed
XM_004023580.3	1.282101604	0.079801577	Over expressed
XR_001040309.3	1.280443703	0.096355195	Over expressed
XM_027974202.1	1.278281248	0.059715981	Over expressed
XR_003590367.1	1.27771742	0.055296389	Over expressed
XM_027962053.1	1.27484816	0.006836925	Over expressed
XM_027967873.1	1.269974049	0.002477824	Over expressed
XM_027960259.1	1.267895244	0.094029764	Over expressed
XR_001435729.2	1.266752489	1.37E-06	Over expressed
XM_012104398.2	1.263251954	0.006164355	Over expressed
XR_003591808.1	1.262900492	0.008768154	Over expressed
XM_027979486.1	1.262591307	0.091428569	Over expressed
XM_004006521.4	1.261503849	0.00220499	Over expressed
XM_027959548.1	1.261179665	0.091788073	Over expressed
XM_027959207.1	1.259192892	0.006047766	Over expressed
XM_015100653.2	1.257620847	0.020084475	Over expressed
XM_004019719.4	1.257590986	0.048660169	Over expressed
XM_004014369.4	1.256832002	0.122511419	Over expressed
XM_027965638.1	1.2565729	0.048781598	Over expressed
XM_004003424.4	1.256054186	0.089062417	Over expressed
XM_027967099.1	1.255552006	0.010896328	Over expressed
XM_004012448.4	1.25519407	0.052379531	Over expressed
XM_012176841.3	1.254777681	0.031672061	Over expressed
XM_027973444.1	1.254198339	0.041973645	Over expressed
XM_027967702.1	1.251410032	0.005429258	Over expressed
XM_004010651.4	1.251404724	0.126095431	Over expressed
XM_004008553.4	1.250608921	0.132221452	Over expressed
XM_027962962.1	1.250601499	0.076325158	Over expressed
XM_027958321.1	1.250363854	0.126992313	Over expressed
XM_027971849.1	1.249352983	0.137603646	Over expressed
XM_027968152.1	1.247753221	0.01433464	Over expressed
XM_027966429.1	1.246956472	0.133490939	Over expressed
XM_015095535.2	1.246891488	2.07E-06	Over expressed
XM_027961939.1	1.24634338	0.147646218	Over expressed
XM_004010314.4	1.245555415	0.088837651	Over expressed
XM_004015312.4	1.245069434	0.002270272	Over expressed
XM_027956904.1	1.24444728	0.061008198	Over expressed
XM_027968942.1	1.24403272	9.18E-05	Over expressed
XM_012096270.3	1.243541022	0.107697796	Over expressed
XM_027974792.1	1.242997398	0.016787434	Over expressed
XM_004007974.2	1.242902798	0.035169692	Over expressed
XM_012190368.3	1.242771977	0.108808946	Over expressed
XM_004021914.4	1.240026711	0.037683243	Over expressed
XM_027960285.1	1.239342182	0.000520444	Over expressed

XR_003590712.1	1.238390351	0.017559004	Over expressed
XM_027961327.1	1.237822215	0.000169433	Over expressed
XM_015095895.2	1.237680339	0.0503123	Over expressed
XM_012102212.3	1.237241669	5.02E-05	Over expressed
XM_012096752.3	1.23606447	0.036187875	Over expressed
XM_004008406.4	1.235355957	0.001285659	Over expressed
XM_027956917.1	1.235043896	0.100174248	Over expressed
XM_027967852.1	1.235038977	0.080283244	Over expressed
XM_004002351.4	1.234780747	0.061847952	Over expressed
XM_027965464.1	1.234051425	0.10197323	Over expressed
XM_004012674.4	1.233378157	0.120651302	Over expressed
XM_012101890.3	1.233284604	0.008864064	Over expressed
XR_003588265.1	1.23239534	0.023939488	Over expressed
XM_027969929.1	1.231401911	0.143253784	Over expressed
XM_027959937.1	1.230244735	0.068944747	Over expressed
XM_027956865.1	1.229998001	0.024241302	Over expressed
XM_027969758.1	1.22830306	9.64E-06	Over expressed
XM_004015179.4	1.227536807	0.120153508	Over expressed
XM_012104342.3	1.22703252	0.095824919	Over expressed
XM_004021260.4	1.226988196	0.01120223	Over expressed
XM_027961340.1	1.226950687	0.025010063	Over expressed
XM_004010451.4	1.225895228	0.050774793	Over expressed
XM_004012238.4	1.225554535	0.105631895	Over expressed
XM_004015209.4	1.225477174	0.036012555	Over expressed
XM_027967935.1	1.224722894	0.037413372	Over expressed
XM_015094704.2	1.223109144	8.32E-05	Over expressed
XM_004020746.4	1.222114975	0.025231937	Over expressed
XM_015104005.2	1.222103963	0.001560273	Over expressed
XM_012099040.3	1.218009806	0.070494162	Over expressed
XM_027970421.1	1.216504137	0.033309104	Over expressed
XM_004018967.4	1.215827149	9.23E-05	Over expressed
XR_003588659.1	1.215596297	0.000223243	Over expressed
XM_012174672.2	1.215136896	0.009363893	Over expressed
XM_027977394.1	1.214459989	0.128740583	Over expressed
XR_003588506.1	1.214357314	0.117572887	Over expressed
XM_012182071.3	1.214042466	0.067666038	Over expressed
XM_027974109.1	1.213624145	0.042918317	Over expressed
XM_027974098.1	1.213133392	0.059988279	Over expressed
XM_027962048.1	1.211768259	0.003621121	Over expressed
XM_027956876.1	1.210433211	0.011511868	Over expressed
NM_001024863.1	1.208462994	0.013559821	Over expressed
XM_004012641.4	1.208356496	0.011241574	Over expressed
XM_027969412.1	1.207776037	0.000596778	Over expressed
XM_004017327.4	1.207525425	0.128885414	Over expressed

XM_027976778.1	1.206180461	0.028161111	Over expressed
XM_004020920.3	1.204484801	0.012213552	Over expressed
XM_004008568.4	1.203478167	0.135667057	Over expressed
XM_012178143.2	1.202994045	0.125007433	Over expressed
XM_027977741.1	1.201438246	1.06E-05	Over expressed
XM_004002631.4	1.19931807	0.013626649	Over expressed
XM_027969323.1	1.199188534	0.14779519	Over expressed
NM_001168634.1	1.198540418	0.002113844	Over expressed
XM_004015038.4	1.19792344	0.046888031	Over expressed
XM_027958386.1	1.197420063	0.137339498	Over expressed
XM_004006291.4	1.195239891	0.086696977	Over expressed
XM_004013563.4	1.195120674	0.002558817	Over expressed
XM_027968184.1	1.19291894	2.33E-05	Over expressed
XM_012184243.2	1.191826603	0.000654075	Over expressed
XM_027975144.1	1.190765135	0.000420255	Over expressed
XM_027978597.1	1.190057424	1.45E-13	Over expressed
XM_004019598.4	1.189634	0.133584439	Over expressed
XM_027960003.1	1.186290746	0.054989114	Over expressed
XM_027961188.1	1.185870724	0.096237181	Over expressed
XM_027974099.1	1.185649207	0.017565173	Over expressed
XM_004008520.4	1.1851041	0.073591388	Over expressed
XM_015098651.2	1.184525236	0.120634334	Over expressed
XR_003586540.1	1.182961681	0.030212212	Over expressed
XM_015103943.2	1.18162072	2.98E-22	Over expressed
XM_027959560.1	1.180642903	0.014505765	Over expressed
XM_004020489.4	1.180532289	0.049456155	Over expressed
XM_027979633.1	1.180487445	0.010557192	Over expressed
XM_027978629.1	1.179555893	1.33E-05	Over expressed
XM_027966316.1	1.179471019	0.012707724	Over expressed
XM_004001786.3	1.178223614	0.001257305	Over expressed
XM_004018938.3	1.177810512	0.007994087	Over expressed
XM_027974303.1	1.177762797	0.113628781	Over expressed
XM_027959544.1	1.177361212	0.035144156	Over expressed
XM_027961930.1	1.175683808	0.003282492	Over expressed
XM_004008697.3	1.175353627	0.14014596	Over expressed
XM_027971599.1	1.175276486	0.022811929	Over expressed
XM_004010536.4	1.174639912	0.049526479	Over expressed
XM_012095529.3	1.171203511	0.133584439	Over expressed
XM_027969496.1	1.16989636	0.041498006	Over expressed
XM_004015186.3	1.168327872	0.028342021	Over expressed
XM_012100976.3	1.167223334	0.008669331	Over expressed
XM_027965715.1	1.166969825	0.025010063	Over expressed
XM_027977122.1	1.166080384	0.000106876	Over expressed
XM_027968167.1	1.16588119	0.075594242	Over expressed

XM_004020756.4	1.165493899	0.041724869	Over expressed
XM_027974269.1	1.163288582	0.070832792	Over expressed
XM_015091821.2	1.163036835	0.011886317	Over expressed
XM_004015142.4	1.162707271	0.134637215	Over expressed
XM_027967566.1	1.159841174	0.139457359	Over expressed
XM_027977096.1	1.158747269	0.146406744	Over expressed
XM_027968225.1	1.15784934	0.015053323	Over expressed
XM_015091944.2	1.157216099	0.009593081	Over expressed
XM_027965235.1	1.15607879	0.052914836	Over expressed
XM_027977461.1	1.155259524	0.002069099	Over expressed
XM_027964059.1	1.154196863	0.01433464	Over expressed
XM_004015229.4	1.153957137	0.046637077	Over expressed
XM_027961864.1	1.152251781	0.060289688	Over expressed
XM_004007255.4	1.15098806	0.059510019	Over expressed
XM_027959899.1	1.150001101	0.003617324	Over expressed
XM_027971323.1	1.149478315	0.107360975	Over expressed
XM_004008565.3	1.149317148	0.105599617	Over expressed
XM_027973054.1	1.148690465	0.010203145	Over expressed
XM_027958630.1	1.147191111	0.111672128	Over expressed
XM_027958839.1	1.146813624	4.03E-05	Over expressed
XM_027968014.1	1.145399312	0.028897473	Over expressed
XM_027978792.1	1.144665347	4.53E-05	Over expressed
XM_004015475.4	1.143363573	0.019544665	Over expressed
XM_015100619.2	1.143363238	0.100318365	Over expressed
XM_027967240.1	1.143315139	0.081116281	Over expressed
XM_027978367.1	1.141940423	0.020180848	Over expressed
XR_001034045.3	1.14152912	0.105599617	Over expressed
XM_027967051.1	1.140619495	0.046812964	Over expressed
XM_027969806.1	1.140404803	0.033958612	Over expressed
XM_027978260.1	1.14039402	0.009910192	Over expressed
XM_004008492.4	1.140045351	0.049526479	Over expressed
XM_012173883.3	1.13917814	3.34E-06	Over expressed
XM_027974075.1	1.138339843	0.007994087	Over expressed
XM_004020893.4	1.13747175	0.0864156	Over expressed
XM_015094997.2	1.137355535	0.039939834	Over expressed
XM_027976774.1	1.137128834	0.001527043	Over expressed
XR_003589941.1	1.136979571	0.034005553	Over expressed
XM_027978753.1	1.136238224	0.046433031	Over expressed
XM_015100473.2	1.135948405	0.113125338	Over expressed
XM_004008603.4	1.135918133	0.095711882	Over expressed
XM_027977252.1	1.135030081	0.090703348	Over expressed
XM_027956901.1	1.134847103	0.024565188	Over expressed
XM_027968560.1	1.133387852	0.126277833	Over expressed
XM_027973663.1	1.131576508	0.004225515	Over expressed

XM_027966724.1	1.129632051	0.011555383	Over expressed
XM_027978300.1	1.129057375	0.003621121	Over expressed
XM_004019684.4	1.12812881	0.000291365	Over expressed
XM_027977614.1	1.125964058	0.060173505	Over expressed
XM_004011143.4	1.124675203	0.049361328	Over expressed
XM_027976142.1	1.123922226	6.25E-05	Over expressed
XM_004004150.3	1.123268567	0.115627782	Over expressed
XM_004002611.4	1.121959628	0.013842457	Over expressed
XM_015104052.2	1.120790324	0.012525433	Over expressed
XM_004008507.4	1.120575754	0.004119139	Over expressed
XM_027963646.1	1.118622344	0.025179409	Over expressed
XM_027979327.1	1.118493845	0.057852004	Over expressed
XM_027972382.1	1.118187047	0.001391057	Over expressed
XM_004010270.4	1.116365068	0.146122524	Over expressed
XM_004023454.3	1.114940255	0.074637024	Over expressed
XM_027968834.1	1.113816851	0.008011398	Over expressed
XM_004015286.4	1.112351604	0.084118225	Over expressed
XM_004015161.2	1.111830761	0.082930467	Over expressed
XM_027959996.1	1.11092976	0.020613284	Over expressed
XM_027965495.1	1.110002454	0.10249294	Over expressed
XM_004013023.3	1.108912231	0.010836182	Over expressed
NM_001142511.2	1.107603576	0.131199935	Over expressed
XM_012104152.3	1.106574228	0.013130177	Over expressed
XM_004002585.4	1.106546759	0.000694924	Over expressed
XM_027977393.1	1.105878265	0.127642024	Over expressed
XM_027971586.1	1.101597775	0.039043909	Over expressed
XM_027978042.1	1.099750742	0.000552087	Over expressed
XM_015094798.2	1.099267817	0.146929191	Over expressed
XM_027959964.1	1.09907047	0.137884717	Over expressed
XM_004010313.4	1.098592158	0.142868413	Over expressed
XM_027959705.1	1.097108503	0.062638247	Over expressed
XM_004019686.4	1.096595895	0.030175535	Over expressed
XM_004020761.4	1.095718274	0.110830866	Over expressed
XM_012105396.3	1.095158746	0.051238831	Over expressed
XM_027965677.1	1.094153066	0.123706657	Over expressed
XM_004008484.4	1.093038696	0.012664043	Over expressed
XM_004017098.4	1.091883872	0.00295007	Over expressed
XM_012104744.3	1.090890663	0.006481182	Over expressed
XM_004006387.4	1.090701562	0.004106846	Over expressed
XM_027976474.1	1.090550022	0.064993576	Over expressed
XM_027961495.1	1.089073094	0.009049771	Over expressed
XM_004017348.4	1.08737066	0.06263427	Over expressed
NM_001166201.1	1.085997437	2.69E-06	Over expressed
NM_001280704.1	1.085686561	0.051238831	Over expressed

XM_004006921.4	1.085465179	0.088837651	Over expressed
XM_004006069.4	1.084845303	0.039497066	Over expressed
XM_004005586.4	1.084550161	0.062982021	Over expressed
XM_027978945.1	1.082589279	0.014886753	Over expressed
XM_012186201.2	1.081295235	0.055956111	Over expressed
XM_027956850.1	1.080468442	0.046520631	Over expressed
XM_004011869.3	1.079941785	0.062748005	Over expressed
XM_004018449.4	1.07807875	0.032699088	Over expressed
XM_004019646.4	1.077740489	0.002232244	Over expressed
NM_001145179.1	1.074423867	0.039487009	Over expressed
XM_027968289.1	1.074084086	0.040317966	Over expressed
XM_015093274.2	1.072858467	0.104680605	Over expressed
XM_004015435.4	1.072340784	0.100318365	Over expressed
XM_012096032.3	1.070124108	0.019720235	Over expressed
XM_027961411.1	1.0700578	0.044299126	Over expressed
XM_004008498.3	1.069333861	0.051826364	Over expressed
XM_004013584.4	1.06912613	0.052693073	Over expressed
XM_027959339.1	1.068812417	0.021164466	Over expressed
XM_012098977.3	1.068469825	0.008456554	Over expressed
XR_003587964.1	1.068396144	0.026896125	Over expressed
XM_027967902.1	1.063893582	0.052224211	Over expressed
XM_027975798.1	1.063747578	0.12256427	Over expressed
XM_027961180.1	1.062168907	0.056711472	Over expressed
NM_001135926.1	1.058839275	0.066138598	Over expressed
XM_012189126.3	1.056349488	0.010237078	Over expressed
XR_003590010.1	1.055288338	0.055213011	Over expressed
XM_004014632.4	1.053375395	0.001337455	Over expressed
XM_027964157.1	1.053304746	0.104981898	Over expressed
XM_027959361.1	1.053003356	0.003285759	Over expressed
XM_027962939.1	1.052997914	0.059466235	Over expressed
XM_027978768.1	1.051301172	0.07218715	Over expressed
NM_001135931.1	1.050807561	0.129080677	Over expressed
XM_027978620.1	1.049893348	0.125839733	Over expressed
XM_027977759.1	1.048411964	0.001714686	Over expressed
XM_004013042.4	1.048242273	0.111172921	Over expressed
XM_012147961.3	1.048095224	3.20E-05	Over expressed
XM_027974314.1	1.046015585	0.032243392	Over expressed
XM_004022064.3	1.044070714	0.094818409	Over expressed
XM_027957871.1	1.044004978	0.002054399	Over expressed
XM_027963352.1	1.043006274	0.135789221	Over expressed
XM_015095053.2	1.042373099	0.095211005	Over expressed
XM_004017846.4	1.040918276	4.82E-05	Over expressed
XM_004015418.4	1.038632257	0.055802697	Over expressed
XM_004005025.3	1.036819012	0.046917179	Over expressed

XM_027958866.1	1.036535548	0.000890618	Over expressed
XM_004005614.4	1.034718923	0.123265146	Over expressed
XM_015104234.2	1.033877262	0.125073826	Over expressed
XM_012099271.2	1.033068731	0.081612208	Over expressed
XM_004020710.4	1.029242084	0.112206663	Over expressed
XM_004016091.4	1.029172933	0.025476205	Over expressed
XM_027966606.1	1.026215239	0.10929768	Over expressed
XM_027959365.1	1.026051294	0.008069249	Over expressed
XM_027961938.1	1.02543977	0.038453148	Over expressed
XM_004022320.4	1.025238019	0.087382476	Over expressed
XM_027967928.1	1.023818383	0.014742046	Over expressed
XR_003588740.1	1.023569084	0.024046223	Over expressed
XM_027960801.1	1.023541508	0.057347633	Over expressed
XM_027969445.1	1.023256906	0.0897164	Over expressed
XM_027975816.1	1.022559486	0.010237078	Over expressed
XM_004019745.4	1.021334579	0.032781004	Over expressed
XM_004008617.4	1.021005192	0.121459753	Over expressed
XM_027966105.1	1.020334351	0.002347029	Over expressed
XM_004008761.4	1.020025885	0.00963251	Over expressed
XM_027967942.1	1.019305296	0.006326504	Over expressed
XM_012184828.3	1.019245041	0.114276131	Over expressed
XM_004018761.4	1.018390945	0.121459753	Over expressed
XM_004013792.4	1.018375016	0.013599888	Over expressed
XM_004013066.4	1.016991623	3.46E-09	Over expressed
XM_004005740.4	1.016064224	0.127399632	Over expressed
XM_027957579.1	1.014382861	0.033190168	Over expressed
XM_004014925.3	1.01309614	0.051076808	Over expressed
XM_004005564.4	1.013066117	0.136552173	Over expressed
XR_003588061.1	1.012259848	0.149536351	Over expressed
XR_003590958.1	1.011147328	0.007163966	Over expressed
XM_027971139.1	1.010478855	0.035590589	Over expressed
XM_027956730.1	1.009636814	0.000105089	Over expressed
XM_027975896.1	1.006429996	0.128337423	Over expressed
XM_004021423.4	1.005807533	0.035725856	Over expressed
XM_015099420.2	1.003250666	0.082227618	Over expressed
XM_004018856.3	1.00319825	0.040192347	Over expressed
XM_027974446.1	1.002098955	0.088374281	Over expressed
XM_015099888.2	1.000430953	0.009939424	Over expressed
XM_012107284.3	-12.21750864	3.87E-14	Under expressed
XR_001044470.3	-12.21521825	0.035052823	Under expressed
XM_004002003.4	-12.17137027	0.001799711	Under expressed
XM_027980063.1	-11.724502	0.018455121	Under expressed
XM_004013579.4	-11.64156576	0.022349694	Under expressed
XM_027966360.1	-11.58378998	0.007163966	Under expressed

XM_027956925.1	-11.27802848	3.70E-07	Under expressed
XM_027968298.1	-11.11034704	0.012987121	Under expressed
NM_001161875.1	-11.05578607	0.010237078	Under expressed
XM_004012386.4	-11.02038409	0.010346108	Under expressed
XM_027973218.1	-10.95645726	0.013316878	Under expressed
XM_027971758.1	-10.9090075	8.63E-12	Under expressed
XM_004004539.4	-10.88696948	1.05E-28	Under expressed
XM_012098775.3	-10.82654073	0.014620963	Under expressed
XM_015102717.2	-10.67874875	0.008617931	Under expressed
XM_004010189.3	-10.66944351	1.05E-28	Under expressed
NM_001267886.1	-10.62543487	0.008682363	Under expressed
XM_027969790.1	-10.60027485	0.007861936	Under expressed
XM_027973680.1	-10.58394686	0.009872716	Under expressed
XM_027975993.1	-10.55034427	0.041016503	Under expressed
XM_027957232.1	-10.53027655	2.54E-18	Under expressed
XM_004006637.4	-10.50760789	0.009954469	Under expressed
XM_027959566.1	-10.46508454	0.000292081	Under expressed
XM_012186977.3	-10.43503046	0.012546177	Under expressed
XM_027968824.1	-10.39938469	0.017597182	Under expressed
XM_027972013.1	-10.3621036	0.006521643	Under expressed
XM_027959215.1	-10.34250915	1.51E-11	Under expressed
XM_012099164.3	-10.32758457	4.85E-10	Under expressed
XM_027975678.1	-10.3207201	0.036052651	Under expressed
XM_027980046.1	-10.31976173	0.009241769	Under expressed
XM_004011171.4	-10.29250491	0.0092467	Under expressed
XM_012167463.3	-10.2888061	0.008257512	Under expressed
XM_027975301.1	-10.28850575	0.010237078	Under expressed
XM_015103799.2	-10.25719309	7.61E-11	Under expressed
XM_027980133.1	-10.20196952	0.009658024	Under expressed
XM_027973669.1	-10.19265347	0.008681624	Under expressed
XM_027959807.1	-10.18417411	0.000188204	Under expressed
XM_027979272.1	-10.17801815	0.053579367	Under expressed
XM_012106391.3	-10.17270159	0.009538179	Under expressed
XM_015096615.2	-10.1685496	0.009671194	Under expressed
XM_027968562.1	-10.14604095	0.000194999	Under expressed
XM_015103476.2	-10.12972541	1.21E-13	Under expressed
XM_027957456.1	-10.1253472	0.008805434	Under expressed
XM_015096599.2	-10.08415178	0.013890188	Under expressed
XM_027964706.1	-10.05323218	0.001136631	Under expressed
XM_004016877.4	-10.02335496	0.017170065	Under expressed
XM_004002019.4	-10.01856334	0.037172299	Under expressed
XM_012189031.3	-10.01551782	0.009354523	Under expressed
XM_012181721.3	-9.986208158	0.008961388	Under expressed
XM_004002679.4	-9.947126928	0.073561248	Under expressed

XM_012181269.2	-9.945090081	0.007994087	Under expressed
XM_012183459.2	-9.923808535	0.008204362	Under expressed
XM_027972995.1	-9.921352257	0.009411735	Under expressed
XM_015098202.2	-9.917594037	0.00936524	Under expressed
XR_003590182.1	-9.914285696	0.020714182	Under expressed
XM_027970465.1	-9.91163552	8.93E-15	Under expressed
XM_027979528.1	-9.91032283	0.018737395	Under expressed
XM_012182486.2	-9.866932078	0.011331293	Under expressed
XM_027972363.1	-9.861984761	0.009000653	Under expressed
XM_027973556.1	-9.812699266	0.012801619	Under expressed
XM_027973321.1	-9.778798235	6.24E-08	Under expressed
XM_027973958.1	-9.768215181	1.25E-15	Under expressed
XM_012178737.2	-9.764213047	5.68E-15	Under expressed
XM_015102675.2	-9.752307111	0.008712863	Under expressed
XM_004002026.4	-9.717252972	0.020714526	Under expressed
XM_012166878.3	-9.62359619	0.010297484	Under expressed
XM_027956283.1	-9.574705732	9.01E-10	Under expressed
XM_012112861.3	-9.56423255	1.80E-05	Under expressed
XM_012177073.3	-9.548998964	0.009396789	Under expressed
XM_015096897.2	-9.546778917	0.021945999	Under expressed
XM_004020467.4	-9.545358989	0.028526464	Under expressed
XM_004016919.4	-9.518852899	6.71E-05	Under expressed
XM_027962549.1	-9.517734228	0.015738733	Under expressed
XR_003586476.1	-9.511867128	2.54E-05	Under expressed
XM_027961854.1	-9.510654656	0.041507888	Under expressed
XM_027967636.1	-9.482711938	5.82E-11	Under expressed
XM_012184461.3	-9.482345744	0.010297484	Under expressed
XM_027980049.1	-9.476906762	1.58E-13	Under expressed
XM_012173562.3	-9.462582446	0.010674907	Under expressed
XM_004018287.4	-9.461210755	0.028217668	Under expressed
XM_027961780.1	-9.446387631	0.007352931	Under expressed
XM_012177137.2	-9.434039232	0.008486004	Under expressed
XM_012181001.3	-9.431339177	0.012878753	Under expressed
XM_004004099.4	-9.427926769	1.43E-23	Under expressed
XM_012180730.3	-9.427266308	4.22E-07	Under expressed
XM_012101164.3	-9.421612855	0.010258972	Under expressed
XM_012096956.2	-9.40791998	1.51E-05	Under expressed
XM_012186384.3	-9.388538752	0.048834017	Under expressed
XM_012141038.3	-9.372288333	0.009379891	Under expressed
XM_027957698.1	-9.371815591	1.29E-24	Under expressed
XM_027962333.1	-9.360372709	5.49E-07	Under expressed
XM_012181836.3	-9.359058714	0.012961531	Under expressed
XM_012180815.3	-9.344036899	8.72E-45	Under expressed
XM_027966495.1	-9.335557497	0.039406816	Under expressed

XM_027964674.1	-9.328927784	0.01070508	Under expressed
XM_027973973.1	-9.323583709	0.009333354	Under expressed
XM_027970437.1	-9.283771373	3.26E-21	Under expressed
XM_012158975.3	-9.282203726	0.017779096	Under expressed
XR_003587031.1	-9.278395605	8.53E-37	Under expressed
XM_015099883.2	-9.274870917	0.009685888	Under expressed
XM_012098687.3	-9.267093136	1.82E-16	Under expressed
XM_004002690.3	-9.245072859	0.01648434	Under expressed
XM_027971978.1	-9.241582058	5.49E-07	Under expressed
XM_027962571.1	-9.227342621	0.016272619	Under expressed
XR_001031435.3	-9.191377757	2.17E-07	Under expressed
XM_027961852.1	-9.178342188	0.009479526	Under expressed
XM_004010490.4	-9.163024733	5.65E-14	Under expressed
XM_004004085.3	-9.162203974	1.12E-06	Under expressed
XM_012182384.3	-9.148958779	7.95E-27	Under expressed
XM_004022036.4	-9.146671173	0.009047483	Under expressed
XM_012188683.3	-9.138875409	0.012526807	Under expressed
XM_027961124.1	-9.127008598	0.013647538	Under expressed
XM_012185066.3	-9.126855975	0.009479526	Under expressed
XM_012096778.3	-9.110854753	0.009939424	Under expressed
XM_012177138.2	-9.062361521	0.011412834	Under expressed
XM_027978710.1	-9.051401685	1.91E-09	Under expressed
XM_015103688.2	-9.043282002	3.38E-07	Under expressed
XM_027974035.1	-9.010529757	0.008681624	Under expressed
XM_027962841.1	-8.993987662	0.011782418	Under expressed
XM_027963308.1	-8.957656701	0.010297484	Under expressed
XM_027969077.1	-8.950914454	0.011162479	Under expressed
XM_027969422.1	-8.928856137	0.011678027	Under expressed
XM_012106919.2	-8.914384574	9.39E-06	Under expressed
XM_027974503.1	-8.888624927	0.017658529	Under expressed
XM_012185560.3	-8.885162801	3.37E-08	Under expressed
XM_027977095.1	-8.863741294	0.020798222	Under expressed
XM_027963539.1	-8.854978596	0.016595766	Under expressed
XM_012185222.3	-8.844034578	0.007275229	Under expressed
XM_027972409.1	-8.843912821	0.010237078	Under expressed
XM_004017934.4	-8.8437191	0.018545723	Under expressed
XM_027957534.1	-8.843587167	1.88E-14	Under expressed
XM_012179718.3	-8.839010133	2.77E-19	Under expressed
XM_012181025.3	-8.822707558	0.010169791	Under expressed
XM_027973650.1	-8.818632837	0.011322098	Under expressed
XM_027959153.1	-8.81485934	6.29E-28	Under expressed
XM_015098132.2	-8.811320253	0.048100684	Under expressed
XM_027969232.1	-8.797255341	0.007398033	Under expressed
XM_027962338.1	-8.786028247	2.54E-18	Under expressed

XM_027967698.1	-8.777174057	0.009134949	Under expressed
XM_004016499.4	-8.766957483	0.000979204	Under expressed
XM_027976675.1	-8.747525284	0.081729291	Under expressed
XM_012179026.2	-8.73017909	0.017229345	Under expressed
XM_027975572.1	-8.726255152	0.007874009	Under expressed
XM_012107667.2	-8.71477183	0.00966697	Under expressed
XM_012096966.2	-8.699488549	3.13E-05	Under expressed
XM_027962422.1	-8.68890241	0.000861088	Under expressed
XM_012096928.3	-8.68029023	0.008363405	Under expressed
XM_015099773.2	-8.678749278	0.016610802	Under expressed
XM_015105013.2	-8.644845867	0.013839825	Under expressed
XM_012132272.3	-8.638719545	0.013689236	Under expressed
XM_027978723.1	-8.63681801	0.025129359	Under expressed
XM_027972374.1	-8.62808852	9.75E-06	Under expressed
XM_027970914.1	-8.620009037	0.02580157	Under expressed
NM_001130937.1	-8.611909536	0.012939778	Under expressed
XM_012102903.3	-8.608892254	0.029903906	Under expressed
XM_027971688.1	-8.607028959	0.017486549	Under expressed
XM_012095278.3	-8.599425019	0.010182337	Under expressed
XM_004011430.4	-8.597948255	0.011964976	Under expressed
XM_027978564.1	-8.588965847	9.17E-08	Under expressed
XM_012187094.2	-8.576175486	0.009613043	Under expressed
XM_012178596.2	-8.576083897	0.011704526	Under expressed
XM_027976136.1	-8.570077312	0.03550659	Under expressed
XM_027969440.1	-8.563163084	0.006931981	Under expressed
XM_012101386.3	-8.539725599	0.03639558	Under expressed
XM_012177785.3	-8.484159246	1.51E-05	Under expressed
XM_027972923.1	-8.475198029	0.009968025	Under expressed
NM_001161863.1	-8.460243981	0.012227837	Under expressed
XM_027972592.1	-8.455853636	4.61E-11	Under expressed
XM_027960981.1	-8.440773945	0.000618342	Under expressed
XM_015096946.2	-8.396156483	0.018727344	Under expressed
XM_012105058.3	-8.39078535	4.16E-78	Under expressed
XM_012186196.3	-8.389506961	8.15E-11	Under expressed
XM_004017259.4	-8.36427919	0.01953568	Under expressed
XM_027970749.1	-8.361446883	0.060934593	Under expressed
XM_012182825.3	-8.355345856	0.01130395	Under expressed
XM_027979257.1	-8.354253683	0.012538451	Under expressed
XM_012188216.3	-8.354118888	8.89E-06	Under expressed
XM_012179569.2	-8.348356583	0.011241574	Under expressed
XM_012184129.3	-8.343956481	0.014327792	Under expressed
XM_027965540.1	-8.327412987	0.00538775	Under expressed
XM_012095359.3	-8.29492462	0.105631895	Under expressed
XM_027970887.1	-8.293021105	0.052730363	Under expressed

XM_027974491.1	-8.267352125	0.014957544	Under expressed
XM_004017226.4	-8.264431965	0.000163976	Under expressed
XM_012180705.2	-8.26045574	0.017406573	Under expressed
XM_004021347.3	-8.254997287	0.013994712	Under expressed
XM_027977518.1	-8.251708038	1.54E-09	Under expressed
XM_027978987.1	-8.225473182	0.043432759	Under expressed
XM_012096181.3	-8.215943777	0.021182952	Under expressed
XM_012188708.3	-8.172357412	0.024878407	Under expressed
XM_027975032.1	-8.169978091	0.008581049	Under expressed
XM_012096404.2	-8.158504374	0.012216998	Under expressed
XM_012188916.2	-8.152107335	0.024899733	Under expressed
XM_027975568.1	-8.09665611	0.02431223	Under expressed
XM_012105509.3	-8.088284485	0.0010131	Under expressed
XM_004009747.4	-8.059502973	0.007994087	Under expressed
XM_027970909.1	-8.037163232	3.72E-17	Under expressed
XM_004004666.3	-8.001240512	8.44E-07	Under expressed
NM_001112818.1	-7.995094328	0.005305707	Under expressed
XM_012178975.3	-7.979734437	5.05E-17	Under expressed
XM_004018359.3	-7.977003516	3.38E-07	Under expressed
XM_027963238.1	-7.94055025	0.012664043	Under expressed
XM_027979373.1	-7.937544827	0.010789577	Under expressed
XM_027971159.1	-7.934088858	0.043085938	Under expressed
XM_012178316.2	-7.932998075	0.019365815	Under expressed
XM_012178375.3	-7.896538844	0.010480428	Under expressed
XM_027960242.1	-7.86953706	0.01455937	Under expressed
XM_015091837.2	-7.865732866	0.02076635	Under expressed
XR_003591027.1	-7.863108686	0.076165361	Under expressed
XM_004011222.3	-7.836022449	0.035744042	Under expressed
XM_027961606.1	-7.833477489	0.023785588	Under expressed
XR_003586905.1	-7.832664545	3.02E-11	Under expressed
XM_027976732.1	-7.796603667	1.96E-05	Under expressed
XM_027963556.1	-7.792944117	0.013999152	Under expressed
XM_027962124.1	-7.787444243	0.017406573	Under expressed
XM_027973781.1	-7.779148208	0.009689926	Under expressed
XM_027979480.1	-7.761480496	0.013071557	Under expressed
XM_027966095.1	-7.754516949	0.021575221	Under expressed
XM_027973000.1	-7.743370728	8.15E-11	Under expressed
XM_027966939.1	-7.74275755	0.012656651	Under expressed
XM_027963867.1	-7.719275719	5.67E-11	Under expressed
XM_012122753.2	-7.703247649	0.014161487	Under expressed
XM_012175400.2	-7.692371789	0.059060401	Under expressed
XM_004020844.4	-7.677535322	1.05E-08	Under expressed
XM_004022319.4	-7.639706392	0.051826364	Under expressed
XM_027976626.1	-7.638045183	0.090925695	Under expressed

XM_012119403.3	-7.626834806	0.032971924	Under expressed
XM_027961083.1	-7.607933152	0.005492587	Under expressed
XM_012190459.3	-7.593019658	0.026896125	Under expressed
XM_004003332.4	-7.591932846	6.25E-05	Under expressed
XM_027966777.1	-7.555107757	0.099721279	Under expressed
XM_012096345.3	-7.535123954	4.79E-06	Under expressed
XM_027977429.1	-7.528387299	0.012467379	Under expressed
XM_027978660.1	-7.515188382	0.04590634	Under expressed
XM_012097057.2	-7.514046133	0.014161487	Under expressed
XM_027967807.1	-7.500921462	0.008864064	Under expressed
XR_003586502.1	-7.488108154	0.012002035	Under expressed
XM_012119432.3	-7.475130366	0.020223385	Under expressed
XM_012096180.3	-7.452721233	0.010012584	Under expressed
XM_004010535.4	-7.424738936	0.018122711	Under expressed
XM_004022177.4	-7.411611931	0.023028172	Under expressed
XM_027967804.1	-7.404906527	0.000844295	Under expressed
XM_027960326.1	-7.331350264	0.03674594	Under expressed
XM_012160811.3	-7.316556593	0.017989728	Under expressed
XM_027972992.1	-7.311622433	0.01556651	Under expressed
XM_027956582.1	-7.304344596	0.010159422	Under expressed
XM_027968697.1	-7.290502863	0.01260265	Under expressed
XM_027964985.1	-7.281081841	0.016021313	Under expressed
XM_012174144.3	-7.27379308	0.01666636	Under expressed
XM_027970571.1	-7.271794073	0.081837807	Under expressed
XM_027969332.1	-7.189841773	0.017658529	Under expressed
XM_015098899.2	-7.188960958	0.013316878	Under expressed
XM_012101865.3	-7.188541721	0.045026491	Under expressed
XM_027970388.1	-7.18476066	0.03158679	Under expressed
XM_027972412.1	-7.13341662	0.000312229	Under expressed
XM_004017197.4	-7.132524307	0.022970593	Under expressed
NM_001162562.1	-7.105881931	0.038616448	Under expressed
XM_027963735.1	-7.093827798	0.059518391	Under expressed
XM_027967029.1	-7.075102911	0.009134949	Under expressed
XM_027978155.1	-7.033152175	0.054004676	Under expressed
XM_027957826.1	-7.022341665	0.000144801	Under expressed
XM_027970693.1	-6.993109642	0.089666629	Under expressed
XM_027968617.1	-6.976511402	0.013163841	Under expressed
XM_015095234.2	-6.953561149	0.046260646	Under expressed
XM_004016510.4	-6.94871377	0.138721593	Under expressed
XM_015099846.2	-6.923504397	0.014635662	Under expressed
XM_027966762.1	-6.916906218	0.026594279	Under expressed
XM_027975891.1	-6.912603361	0.057974438	Under expressed
XM_015103130.2	-6.900688923	0.051563883	Under expressed
XM_012174548.2	-6.858491036	0.004144246	Under expressed

XM_027964420.1	-6.830892166	0.033102655	Under expressed
XM_012153155.3	-6.818166507	4.41E-06	Under expressed
XM_012188684.3	-6.816008592	0.001665501	Under expressed
XM_027972519.1	-6.806747314	0.000582469	Under expressed
XM_027967806.1	-6.755051736	0.028869287	Under expressed
XM_012105545.2	-6.624465467	0.10641625	Under expressed
XM_027965855.1	-6.608228329	0.082101819	Under expressed
XM_015099342.2	-6.577671774	0.000457809	Under expressed
XM_004002670.4	-6.547582663	7.60E-09	Under expressed
NM_001129735.1	-6.491410575	0.003904494	Under expressed
XM_027975516.1	-6.481626338	0.060691503	Under expressed
XM_012097256.3	-6.457640107	0.014012404	Under expressed
XM_015093117.2	-6.45038556	0.102690495	Under expressed
XR_001041729.2	-6.444011004	0.011386162	Under expressed
XM_012096086.3	-6.385646425	0.107697796	Under expressed
XM_012096676.2	-6.383594938	0.01433464	Under expressed
NM_001009241.1	-6.344646655	0.077524379	Under expressed
XM_027967441.1	-6.290485656	0.001393315	Under expressed
XM_027974343.1	-6.280464483	0.143047857	Under expressed
XM_027966753.1	-6.273388198	0.050774793	Under expressed
XM_004020845.3	-6.215657331	0.000238107	Under expressed
XM_012095100.2	-6.170739949	0.052886625	Under expressed
XM_004013958.4	-6.166517086	0.085798345	Under expressed
XR_003586500.1	-6.129370186	0.070494162	Under expressed
XM_004012878.4	-6.112441578	0.07677153	Under expressed
XM_004010192.4	-6.080373544	5.56E-06	Under expressed
XM_015102288.2	-6.06191625	6.02E-07	Under expressed
XM_015104334.2	-6.010292628	0.070490227	Under expressed
XM_027958880.1	-6.007323764	0.13405549	Under expressed
XM_027957771.1	-5.992715285	0.041973645	Under expressed
XM_027980054.1	-5.989651049	1.62E-24	Under expressed
XM_015104435.2	-5.965362681	6.73E-06	Under expressed
XM_012185551.3	-5.924973964	0.042807627	Under expressed
XM_004013806.3	-5.861554593	0.081837807	Under expressed
XM_027967168.1	-5.852878377	0.00488023	Under expressed
XR_003591174.1	-5.847494949	0.054391829	Under expressed
XM_027972165.1	-5.822712257	0.079801577	Under expressed
XM_027972362.1	-5.776971465	0.04270552	Under expressed
XM_012105243.3	-5.767712103	0.045166005	Under expressed
XM_027972908.1	-5.729308237	0.016029521	Under expressed
XM_004013612.4	-5.714236664	0.002688439	Under expressed
XM_027967473.1	-5.70925125	0.055677671	Under expressed
XM_012183078.3	-5.660477908	0.015682334	Under expressed
XM_004013839.3	-5.620968566	0.001506081	Under expressed

XM_027975251.1	-5.599134448	0.005698539	Under expressed
XM_015096572.2	-5.593207489	0.114050461	Under expressed
XM_012178689.3	-5.583349567	0.130658931	Under expressed
XM_012104587.3	-5.556578114	0.024832561	Under expressed
XM_012178585.3	-5.547411622	0.105599617	Under expressed
XR_001041766.3	-5.545960406	0.046514385	Under expressed
XM_012187651.2	-5.536198185	0.062261263	Under expressed
XM_004002929.4	-5.515337916	0.0271568	Under expressed
XM_027967502.1	-5.504625511	0.004094489	Under expressed
XM_027962105.1	-5.397400119	0.031014591	Under expressed
XM_004016930.4	-5.36892955	4.41E-05	Under expressed
XM_004007889.4	-5.345204399	0.138021514	Under expressed
XM_027971199.1	-5.328789918	0.122696693	Under expressed
XR_003588133.1	-5.321505371	0.094230576	Under expressed
XR_003591779.1	-5.320181376	0.082654258	Under expressed
XM_027976400.1	-5.319456578	0.033993234	Under expressed
XR_003586427.1	-5.299362635	1.11E-05	Under expressed
XM_027966141.1	-5.296503614	0.114734629	Under expressed
XM_027971071.1	-5.282542664	0.07833658	Under expressed
XM_027961922.1	-5.265188151	0.127925516	Under expressed
XM_015099709.2	-5.262814628	2.60E-05	Under expressed
NM_001040286.1	-5.259174625	9.08E-09	Under expressed
XM_012096734.3	-5.258457618	0.142857232	Under expressed
XM_027964413.1	-5.234354635	3.10E-05	Under expressed
XM_012173839.3	-5.221999542	0.11401293	Under expressed
XM_012187843.3	-5.213209347	0.137339498	Under expressed
XM_027971470.1	-5.179511048	0.063903909	Under expressed
XM_027976275.1	-5.164313092	0.127620913	Under expressed
XM_027957924.1	-5.134613185	0.04962176	Under expressed
XR_003589456.1	-5.107079061	0.005257165	Under expressed
XM_027962288.1	-5.098518884	0.096996407	Under expressed
XR_003587012.1	-5.092158818	0.01097913	Under expressed
XM_027977321.1	-5.07629899	0.027095428	Under expressed
XM_015101844.2	-5.07499549	0.102275821	Under expressed
XM_012178373.2	-5.062739765	0.147774338	Under expressed
XM_027956989.1	-5.042722469	0.078748788	Under expressed
XR_003586526.1	-5.033955307	0.101138774	Under expressed
XM_012179760.2	-5.008218288	0.043427162	Under expressed
XM_027970573.1	-5.006049602	0.086048531	Under expressed
XM_027957700.1	-4.983950254	0.073591388	Under expressed
XM_027976272.1	-4.959473353	0.024241302	Under expressed
XM_027960637.1	-4.957839353	0.093284742	Under expressed
XM_027977912.1	-4.954250533	0.076791544	Under expressed
XM_012098870.2	-4.919067464	0.043359338	Under expressed

XM_004016241.3	-4.91225828	0.072504325	Under expressed
XM_004017927.4	-4.906074862	1.88E-06	Under expressed
XM_027979948.1	-4.896088043	0.072817495	Under expressed
NM_001306119.1	-4.895656927	0.04452835	Under expressed
XM_012097458.3	-4.87680562	0.008469132	Under expressed
XM_027976731.1	-4.855324591	0.122621563	Under expressed
XM_027965987.1	-4.854367249	0.10197323	Under expressed
XM_015098260.2	-4.827243155	0.006047766	Under expressed
XM_004016165.4	-4.811898358	0.136176227	Under expressed
XM_015101316.2	-4.800369623	0.039650993	Under expressed
XM_027968528.1	-4.772992515	0.08185758	Under expressed
XM_027968756.1	-4.764657446	0.031508716	Under expressed
XM_012102946.3	-4.756806608	0.016999286	Under expressed
XM_027963394.1	-4.740287247	0.107687252	Under expressed
XM_027969914.1	-4.714799258	0.05098375	Under expressed
XM_012103062.3	-4.687994399	0.046204095	Under expressed
XM_015097842.2	-4.681145519	0.036203875	Under expressed
XM_027964608.1	-4.638120364	0.134637215	Under expressed
XR_003588694.1	-4.626736991	0.000113335	Under expressed
XM_012177589.3	-4.625315913	0.029106149	Under expressed
XM_027973620.1	-4.625302986	0.034812776	Under expressed
XR_001039498.3	-4.619451147	3.37E-25	Under expressed
XM_012179622.3	-4.613267574	0.088175326	Under expressed
NM_001009339.1	-4.608449485	0.123593076	Under expressed
XM_004002224.3	-4.592963287	0.082945208	Under expressed
NM_001145178.1	-4.571609749	0.000343544	Under expressed
XM_027958956.1	-4.57129581	0.117022896	Under expressed
XR_003590743.1	-4.552744277	0.059374232	Under expressed
XM_015100446.2	-4.540233945	0.071725236	Under expressed
XR_003591315.1	-4.498961729	0.006803773	Under expressed
XM_012178876.3	-4.496507253	0.038217416	Under expressed
XR_003590880.1	-4.496117743	0.01120223	Under expressed
XM_004002877.4	-4.477126878	0.095824919	Under expressed
XM_027971084.1	-4.470258332	0.146405568	Under expressed
XM_027964019.1	-4.465080678	0.136015816	Under expressed
XR_003586524.1	-4.463113772	0.06570014	Under expressed
XM_015092133.2	-4.45911492	0.118464172	Under expressed
XM_027968128.1	-4.45436624	0.137664572	Under expressed
XM_027959934.1	-4.453574444	0.123706657	Under expressed
XM_004016162.4	-4.447394243	0.082930467	Under expressed
XM_015093479.2	-4.423073714	0.06612904	Under expressed
XM_027966560.1	-4.420406096	1.95E-07	Under expressed
XM_027977125.1	-4.392511755	0.000644421	Under expressed
XM_012117704.3	-4.3909524	0.070939177	Under expressed

XM_012097668.2	-4.386149815	0.028744159	Under expressed
XM_012116929.3	-4.369234177	0.003224408	Under expressed
XM_027968629.1	-4.33976227	0.05578336	Under expressed
XM_027956390.1	-4.339473941	0.117301299	Under expressed
XM_027970711.1	-4.338071395	0.111212159	Under expressed
XM_027964191.1	-4.337599131	0.098453336	Under expressed
XM_004004534.4	-4.316750856	0.004642387	Under expressed
XM_004013227.4	-4.29821483	0.135704162	Under expressed
NM_001009763.1	-4.284619812	0.031508716	Under expressed
XR_003591795.1	-4.283450622	0.001391148	Under expressed
XM_027957967.1	-4.232119679	0.050943756	Under expressed
XM_015103394.2	-4.215759455	0.082930467	Under expressed
XM_012184115.3	-4.186862225	0.032060989	Under expressed
XM_027963535.1	-4.172406946	0.037073994	Under expressed
XM_027958888.1	-4.160954177	0.076791544	Under expressed
XM_027964418.1	-4.147972062	0.098121422	Under expressed
XR_003590477.1	-4.113037726	0.084518502	Under expressed
XM_027958009.1	-4.097341308	1.22E-05	Under expressed
XR_003589581.1	-4.092741503	0.097239536	Under expressed
XM_027975429.1	-4.084370141	0.120415948	Under expressed
XR_003587626.1	-4.084100036	0.022102665	Under expressed
XM_015096507.2	-4.074941039	0.045868386	Under expressed
XM_027966831.1	-4.071513715	0.008252168	Under expressed
XM_027965853.1	-4.070681032	0.093867612	Under expressed
XM_012099477.2	-4.058640741	0.148620498	Under expressed
XR_003590754.1	-4.015771876	0.025242388	Under expressed
NM_001038622.1	-4.008866266	9.62E-07	Under expressed
XM_004011358.4	-4.006446299	0.054700532	Under expressed
XM_012176795.2	-4.005325461	0.009921819	Under expressed
XM_027957226.1	-3.994745115	0.10706153	Under expressed
XM_027968364.1	-3.98753412	0.11382484	Under expressed
XM_004004537.4	-3.983303641	0.018282009	Under expressed
XR_003590267.1	-3.981667257	0.008845687	Under expressed
XM_004020464.4	-3.966429263	0.141928052	Under expressed
XM_012181465.3	-3.9626878	0.078271933	Under expressed
XM_004014567.4	-3.956091555	0.149437976	Under expressed
XR_001434733.2	-3.951334926	0.053683983	Under expressed
XR_001433796.2	-3.943241584	0.133011493	Under expressed
XR_003589488.1	-3.942383391	0.087444049	Under expressed
XM_004022406.4	-3.935674342	0.071849297	Under expressed
XM_012104134.3	-3.928604138	0.070416907	Under expressed
XM_012186999.3	-3.927280865	0.053156568	Under expressed
XR_003587561.1	-3.921413941	0.067525111	Under expressed
XM_027977570.1	-3.921146699	0.106605467	Under expressed

XR_003586579.1	-3.919461192	0.051798465	Under expressed
XM_027961733.1	-3.917070343	0.122098693	Under expressed
XM_027958728.1	-3.914014313	0.000339147	Under expressed
XM_027971753.1	-3.913861221	0.034812776	Under expressed
XM_012099026.3	-3.911197041	0.020582879	Under expressed
XM_004002077.4	-3.903718741	0.022102665	Under expressed
XM_027973595.1	-3.903382311	1.28E-05	Under expressed
XM_012103274.3	-3.902433763	0.000168012	Under expressed
XM_027979678.1	-3.887285642	0.012538451	Under expressed
XR_003586875.1	-3.881203917	0.081834057	Under expressed
XM_012190354.3	-3.868775271	0.058934401	Under expressed
XM_015102700.2	-3.857177483	0.039508055	Under expressed
XM_027970861.1	-3.838938973	0.001594503	Under expressed
XM_027980319.1	-3.824053699	0.043073883	Under expressed
XM_027980252.1	-3.794517938	0.025405313	Under expressed
XM_004014990.4	-3.791642614	0.12534926	Under expressed
XM_027964736.1	-3.791482771	0.023732179	Under expressed
XM_027972650.1	-3.790014153	0.00017145	Under expressed
XR_003591311.1	-3.775932339	0.094439677	Under expressed
XM_027970582.1	-3.760495257	0.133490939	Under expressed
XM_027973868.1	-3.746941888	0.13313411	Under expressed
XM_004002928.4	-3.733534617	0.077921291	Under expressed
XM_027959031.1	-3.726656542	0.132782558	Under expressed
XM_015094933.2	-3.717111733	0.075299708	Under expressed
XM_012184537.3	-3.710038197	0.094967488	Under expressed
XM_027966645.1	-3.709657728	0.0010131	Under expressed
XM_027975152.1	-3.697369774	0.128596432	Under expressed
XM_027964792.1	-3.696179755	0.006495216	Under expressed
XM_015102004.2	-3.696133539	0.051642296	Under expressed
XM_027963141.1	-3.674697338	0.140326578	Under expressed
XR_003590498.1	-3.67393441	0.114734629	Under expressed
XM_027972150.1	-3.670585323	0.038626055	Under expressed
XM_015099371.2	-3.662180881	0.065325801	Under expressed
XM_027962796.1	-3.661322494	0.057812527	Under expressed
XM_027971473.1	-3.65508183	0.025010063	Under expressed
XM_027971093.1	-3.649371006	0.083725294	Under expressed
XM_004021920.4	-3.644373617	0.139019656	Under expressed
XM_012099381.3	-3.643875598	0.079710872	Under expressed
XM_027972889.1	-3.633179087	0.069503187	Under expressed
XM_012186045.3	-3.631338841	0.08771212	Under expressed
XM_027963440.1	-3.629717702	0.093360417	Under expressed
XM_012099802.3	-3.627752206	0.001019594	Under expressed
XM_027977107.1	-3.617828959	0.001814838	Under expressed
XR_003588425.1	-3.594520085	0.10197323	Under expressed

XM_015099701.2	-3.58169075	0.127620913	Under expressed
XM_004007898.4	-3.576669541	0.046917179	Under expressed
XM_012139105.3	-3.575719542	0.033198502	Under expressed
XM_027980305.1	-3.567150396	0.012213552	Under expressed
XM_027962289.1	-3.544968183	0.035033069	Under expressed
XM_027972582.1	-3.541745857	1.80E-05	Under expressed
XR_003591089.1	-3.539139825	0.145341556	Under expressed
XM_012145231.3	-3.536287079	0.031159511	Under expressed
XM_027969368.1	-3.533184511	0.112501745	Under expressed
XM_027970993.1	-3.528187457	0.11401293	Under expressed
XM_015097680.2	-3.51199581	0.116871668	Under expressed
XM_027962716.1	-3.507156224	0.142679959	Under expressed
XR_003591766.1	-3.492910636	0.03760585	Under expressed
XR_003586552.1	-3.479907799	0.084608164	Under expressed
XM_027960518.1	-3.475013255	5.47E-07	Under expressed
XM_027960409.1	-3.469677258	0.127995335	Under expressed
XM_027960039.1	-3.469204981	0.11980626	Under expressed
XM_027979577.1	-3.467332949	0.002805783	Under expressed
XM_027963688.1	-3.466993912	0.016803344	Under expressed
XM_012105328.3	-3.458889129	0.108417189	Under expressed
XM_027968349.1	-3.454043339	0.027309206	Under expressed
XM_027962740.1	-3.450450733	0.019507638	Under expressed
XR_003589761.1	-3.446531368	0.082654258	Under expressed
XR_003585663.1	-3.434358453	0.003627491	Under expressed
XM_012177580.3	-3.419447675	0.007163966	Under expressed
XM_012117978.3	-3.416906093	0.011309071	Under expressed
XM_012100965.3	-3.407054901	0.053574869	Under expressed
XM_027957301.1	-3.406830385	0.142139877	Under expressed
XM_027958354.1	-3.398869072	0.117347728	Under expressed
NM_001174111.1	-3.397648985	0.009009317	Under expressed
XM_027971422.1	-3.387058536	0.03909164	Under expressed
XM_027972738.1	-3.375799097	0.026978676	Under expressed
XM_012187838.3	-3.370146967	0.108417189	Under expressed
XM_004022060.4	-3.363054254	0.070494162	Under expressed
XM_027970459.1	-3.362838415	0.127399632	Under expressed
XM_004006693.4	-3.358534933	0.0001793	Under expressed
XM_012106636.3	-3.356901956	0.144023509	Under expressed
XM_027978118.1	-3.353571286	0.113352537	Under expressed
XM_004016161.4	-3.350150835	0.100710785	Under expressed
XM_004009623.4	-3.336406356	0.091246676	Under expressed
XM_027963983.1	-3.332484473	0.124595136	Under expressed
XM_027965908.1	-3.331836409	0.084473785	Under expressed
XM_004008639.4	-3.321957672	0.136068274	Under expressed
XR_003590311.1	-3.315467417	0.010688741	Under expressed

XR_003590050.1	-3.313976172	0.112903455	Under expressed
XM_004016381.4	-3.313442904	0.026374953	Under expressed
XM_004021393.4	-3.309617302	0.013316878	Under expressed
XM_027977876.1	-3.308356202	0.103472892	Under expressed
XM_027972514.1	-3.307450079	0.057445438	Under expressed
XR_003589712.1	-3.301212055	0.147632137	Under expressed
XM_012096836.3	-3.29629812	0.10197323	Under expressed
XM_015097833.2	-3.289021629	0.096087619	Under expressed
XR_003591780.1	-3.287911571	0.041613764	Under expressed
XM_004021474.4	-3.283155733	0.077188914	Under expressed
XM_027978376.1	-3.278703154	0.06570014	Under expressed
XM_012181325.3	-3.275927721	0.061431822	Under expressed
XM_027970939.1	-3.272451783	0.111212159	Under expressed
XM_015101283.2	-3.270020309	0.048420843	Under expressed
XM_027976539.1	-3.259658401	0.046658722	Under expressed
XM_012175760.3	-3.252964112	0.027286394	Under expressed
XM_012178891.2	-3.235582237	0.047387753	Under expressed
XM_015105087.2	-3.233464689	0.046520631	Under expressed
XM_012182232.3	-3.228714498	0.101186784	Under expressed
XR_001044627.2	-3.227871477	0.020985767	Under expressed
XM_004004867.4	-3.226709258	0.02643817	Under expressed
XM_015092009.2	-3.225455035	0.034028849	Under expressed
XM_015096788.2	-3.219791885	0.061571085	Under expressed
XR_003589137.1	-3.216510861	0.146251529	Under expressed
XM_027970561.1	-3.215599122	0.074918443	Under expressed
XM_004017011.4	-3.210387295	0.001695058	Under expressed
XM_027963738.1	-3.210363642	0.036953517	Under expressed
XM_027964965.1	-3.208704179	0.001070761	Under expressed
XR_003588708.1	-3.207760086	0.127298693	Under expressed
XM_027974688.1	-3.204012992	0.098258683	Under expressed
XM_027960751.1	-3.195043812	0.10711526	Under expressed
NM_001305890.1	-3.192221234	0.129296313	Under expressed
XM_015098156.2	-3.189595826	0.133159195	Under expressed
XM_004017190.4	-3.188917988	0.028441002	Under expressed
XM_012178176.3	-3.186330533	0.031508716	Under expressed
XM_027974864.1	-3.184604757	4.43E-05	Under expressed
XM_027970980.1	-3.178417568	0.028644199	Under expressed
XM_012190050.3	-3.176700974	0.115757473	Under expressed
XM_012186553.2	-3.160105448	0.122098693	Under expressed
XM_027968443.1	-3.159862787	0.06475597	Under expressed
XM_027975070.1	-3.159490922	0.082852122	Under expressed
XM_012176569.3	-3.155284503	0.085187659	Under expressed
XM_027957398.1	-3.135249844	0.05288789	Under expressed
XM_027979335.1	-3.133554981	0.06263427	Under expressed

XM_015094472.2	-3.133298621	0.043427162	Under expressed
XM_015096185.2	-3.126966356	0.080099051	Under expressed
XR_003586839.1	-3.124327114	0.009009317	Under expressed
XM_012106269.3	-3.116628425	0.10561358	Under expressed
XM_027978984.1	-3.116258488	0.000144801	Under expressed
XR_003589957.1	-3.102322805	0.065169794	Under expressed
XM_027976375.1	-3.101619357	0.00102176	Under expressed
XM_012096967.3	-3.092725286	0.140691344	Under expressed
XR_003589811.1	-3.091558701	0.017406573	Under expressed
XM_004010647.4	-3.090520542	0.018422269	Under expressed
XM_004007932.4	-3.078511389	0.143253784	Under expressed
XM_004016457.4	-3.077677606	0.095995136	Under expressed
XM_004017080.4	-3.06737564	0.122511419	Under expressed
XM_012174584.3	-3.066988961	9.92E-09	Under expressed
XM_004004742.4	-3.066652357	0.007248281	Under expressed
XM_027973686.1	-3.066558519	0.015619392	Under expressed
XR_003588688.1	-3.066330162	0.108105478	Under expressed
XM_015104474.2	-3.065614029	0.117572887	Under expressed
XR_003588233.1	-3.063008958	0.093173579	Under expressed
XM_012102454.3	-3.049856239	0.080210851	Under expressed
XM_015097696.2	-3.049809109	0.127289533	Under expressed
XM_027961562.1	-3.049159929	0.032858196	Under expressed
XM_015101039.2	-3.041513004	0.01556651	Under expressed
XM_027959799.1	-3.041289864	0.113628781	Under expressed
XR_003588720.1	-3.039276922	0.089516665	Under expressed
XM_012172342.2	-3.037180628	0.006051791	Under expressed
XM_027960509.1	-3.030955543	0.047825491	Under expressed
XM_027976000.1	-3.029543733	0.105083273	Under expressed
XM_027965712.1	-3.027155635	0.149734613	Under expressed
XM_015102411.2	-3.02254405	0.125787596	Under expressed
XM_015095217.2	-3.022329286	0.006047766	Under expressed
XM_004017952.4	-3.021577276	2.77E-05	Under expressed
XM_004006842.4	-3.009760407	0.065004996	Under expressed
XM_012096467.2	-3.001483372	0.01713513	Under expressed
XM_027976383.1	-2.995486192	0.125527597	Under expressed
XR_003587623.1	-2.991121917	0.133956634	Under expressed
XM_015101375.2	-2.989669455	0.020033532	Under expressed
XM_004019533.3	-2.983752628	0.052049823	Under expressed
XM_027965055.1	-2.982547251	0.122687386	Under expressed
XM_015097698.2	-2.979825994	0.000643223	Under expressed
XR_001039592.3	-2.979025268	0.043089562	Under expressed
XM_027977851.1	-2.978444433	0.144023509	Under expressed
XM_004005133.4	-2.972625541	0.052914836	Under expressed
XM_012142240.2	-2.969465451	0.080938909	Under expressed

XM_004009079.4	-2.963001589	0.044142627	Under expressed
XM_012187117.3	-2.962446092	0.115757473	Under expressed
XM_015105144.2	-2.961755408	0.053799132	Under expressed
XM_015095723.2	-2.958063027	0.018422269	Under expressed
XM_012178337.3	-2.956617528	0.000936587	Under expressed
XM_027956589.1	-2.950269944	0.146616147	Under expressed
XM_027960945.1	-2.945580376	0.128740583	Under expressed
XM_012181722.3	-2.941995163	0.069503187	Under expressed
XM_015099403.2	-2.941826856	0.001560815	Under expressed
XM_027958177.1	-2.940894476	0.140223705	Under expressed
XM_027971035.1	-2.938683756	0.108201965	Under expressed
XM_027975887.1	-2.935500584	0.095711882	Under expressed
XM_004021402.4	-2.933965911	0.066419921	Under expressed
XM_004003008.3	-2.924147603	0.091784069	Under expressed
XM_012106519.3	-2.920269163	0.006778374	Under expressed
XM_015104557.2	-2.914289984	0.026507839	Under expressed
XM_015094353.2	-2.910907858	0.001357238	Under expressed
XM_027961443.1	-2.909639663	0.037978177	Under expressed
XM_027971471.1	-2.906331005	0.09363129	Under expressed
XM_027964724.1	-2.904974909	0.019535061	Under expressed
XM_012094667.3	-2.899284842	0.102575101	Under expressed
XM_004010194.4	-2.897276643	1.23E-05	Under expressed
XM_012180994.3	-2.897152344	0.08540445	Under expressed
XM_004005978.3	-2.893315462	3.05E-05	Under expressed
XM_015094718.2	-2.893031525	0.048934257	Under expressed
XM_027967658.1	-2.890724954	0.00115211	Under expressed
XM_004007785.4	-2.884613216	0.099762831	Under expressed
XM_012183909.2	-2.882168261	0.081729291	Under expressed
XM_015101322.2	-2.872292264	0.003624264	Under expressed
XM_012150644.2	-2.870306127	0.025020946	Under expressed
XM_012102001.3	-2.870115715	0.143047857	Under expressed
XM_004011272.4	-2.868214287	0.102344539	Under expressed
XM_027963815.1	-2.866045454	0.076791544	Under expressed
XM_004015127.4	-2.861127358	0.008296233	Under expressed
XM_027966406.1	-2.854631581	0.128839736	Under expressed
XM_027968532.1	-2.853230158	0.11490007	Under expressed
XM_012174434.3	-2.85242705	0.007646818	Under expressed
XM_015098220.2	-2.849886196	0.090169349	Under expressed
XM_027971225.1	-2.848433548	0.024749164	Under expressed
XM_015099745.2	-2.844699679	0.090061595	Under expressed
XR_003588148.1	-2.842273265	0.081834057	Under expressed
XM_004017332.4	-2.842272109	0.057223023	Under expressed
XM_027966720.1	-2.840952152	0.120995794	Under expressed
XM_015091903.2	-2.831884128	1.33E-06	Under expressed

XM_012099831.3	-2.831172082	0.140048768	Under expressed
XM_027965014.1	-2.827638299	0.008234738	Under expressed
XM_027958615.1	-2.82199508	0.017875212	Under expressed
XM_004010486.4	-2.817899534	0.092427625	Under expressed
XM_004017500.3	-2.81061786	0.006898071	Under expressed
XM_027961395.1	-2.80423492	0.111401573	Under expressed
XM_027964251.1	-2.804180235	0.074690408	Under expressed
XM_012098166.3	-2.795733066	0.03881947	Under expressed
XM_012104365.3	-2.791472939	0.0143192	Under expressed
XM_027965856.1	-2.791296368	0.124879075	Under expressed
NM_001162557.1	-2.781540794	0.000979204	Under expressed
XM_027963476.1	-2.781439439	0.017344171	Under expressed
XM_027968481.1	-2.780319183	0.033731472	Under expressed
XM_027960212.1	-2.780063428	0.055890225	Under expressed
XM_027958873.1	-2.775475945	0.067688084	Under expressed
XM_004004787.4	-2.766563457	0.111498645	Under expressed
XM_027956256.1	-2.764837428	0.020335664	Under expressed
XR_003589154.1	-2.752273474	0.141339378	Under expressed
XM_004006885.4	-2.751033618	0.110345465	Under expressed
XM_012179769.2	-2.750046327	0.117761176	Under expressed
XM_004012185.3	-2.749889997	0.053979527	Under expressed
XR_003585800.1	-2.749097774	3.28E-05	Under expressed
XM_015092359.2	-2.748788597	0.043063167	Under expressed
NM_001009404.1	-2.748148411	0.087444049	Under expressed
XR_003591283.1	-2.733209695	0.024435478	Under expressed
XM_004016356.3	-2.729675019	0.007398033	Under expressed
XM_015097512.2	-2.729385209	0.071622917	Under expressed
XM_015102549.2	-2.725579314	0.00064948	Under expressed
XM_015099338.2	-2.724899336	0.115180028	Under expressed
XM_027971277.1	-2.721555626	4.45E-05	Under expressed
XM_027960408.1	-2.718265588	0.009470702	Under expressed
XM_027980257.1	-2.717550937	0.134485445	Under expressed
XM_004011770.4	-2.716699728	0.017793199	Under expressed
XM_004017895.4	-2.713325191	0.002442182	Under expressed
XM_004022047.4	-2.704260925	0.00030712	Under expressed
XM_027975157.1	-2.703860655	0.075077706	Under expressed
XM_027962179.1	-2.702220831	0.117761176	Under expressed
XM_012178589.3	-2.702121081	0.016180215	Under expressed
XM_027971773.1	-2.699717009	0.116757225	Under expressed
XM_027964486.1	-2.698333957	0.111498645	Under expressed
XM_012189779.3	-2.698275958	0.001507388	Under expressed
XM_027976103.1	-2.697591485	0.120958033	Under expressed
XM_027980217.1	-2.696653225	0.020714526	Under expressed
XM_015092274.2	-2.696134225	0.003330493	Under expressed

XM_027964526.1	-2.695235777	0.137957869	Under expressed
XM_027960540.1	-2.690780127	0.001160346	Under expressed
XM_027962857.1	-2.690198409	0.000710522	Under expressed
XM_027970562.1	-2.688123639	1.51E-06	Under expressed
XM_027975308.1	-2.685237744	0.146590833	Under expressed
XM_027958780.1	-2.684022597	0.132439213	Under expressed
XM_015096606.2	-2.679445696	0.109608148	Under expressed
XM_012184389.3	-2.678057191	0.031508716	Under expressed
XM_027964163.1	-2.672443334	0.114276131	Under expressed
XM_027977136.1	-2.661503097	0.014161487	Under expressed
XR_003588410.1	-2.660617332	0.067325675	Under expressed
XM_015101295.2	-2.658870798	0.000124912	Under expressed
XM_004011234.4	-2.652565303	0.000582665	Under expressed
XM_027962502.1	-2.651771148	0.134129884	Under expressed
XM_027958622.1	-2.649031051	0.114734629	Under expressed
XR_003588119.1	-2.64795633	0.018634073	Under expressed
XM_004004178.4	-2.644690047	0.00078933	Under expressed
XM_027965546.1	-2.642309598	0.080817572	Under expressed
XM_004007775.4	-2.637821658	0.098121422	Under expressed
XM_015096352.2	-2.633943431	0.121570771	Under expressed
XM_004022206.4	-2.633101399	0.043196012	Under expressed
XM_027957154.1	-2.630850027	0.10561358	Under expressed
XM_027976373.1	-2.628656204	0.04916893	Under expressed
XM_004011406.4	-2.62860521	0.008900524	Under expressed
XM_027975878.1	-2.625881995	0.129718133	Under expressed
XM_027962807.1	-2.625476802	0.123643194	Under expressed
XM_027964974.1	-2.624922092	0.11785419	Under expressed
XM_015092231.2	-2.623095354	0.051430511	Under expressed
XM_004016163.3	-2.620118737	0.004889329	Under expressed
XM_004011373.3	-2.61854611	0.049857534	Under expressed
XM_027957576.1	-2.618335913	3.55E-06	Under expressed
XM_012102009.3	-2.615504856	0.052342651	Under expressed
XM_027962054.1	-2.611001956	0.110715545	Under expressed
XM_027976831.1	-2.605729691	0.020340668	Under expressed
XM_012189153.3	-2.603492493	0.087881703	Under expressed
XM_012178660.3	-2.598026485	0.03703447	Under expressed
XM_004011704.4	-2.593630166	0.137339498	Under expressed
XM_004003222.4	-2.591321484	3.17E-14	Under expressed
XM_027975644.1	-2.591089763	0.102443706	Under expressed
XM_027973105.1	-2.589378575	0.000703953	Under expressed
NM_001093785.2	-2.583659208	0.029824081	Under expressed
XM_027979397.1	-2.581538124	0.051900231	Under expressed
XM_012176896.3	-2.58127935	0.020781231	Under expressed
NM_001287473.1	-2.578217586	0.017734551	Under expressed

XM_004010629.4	-2.57791457	0.001259681	Under expressed
XM_027970753.1	-2.577435191	0.023939488	Under expressed
XM_027979147.1	-2.577370867	0.124147993	Under expressed
XM_027964768.1	-2.576900176	0.084350196	Under expressed
NM_001166179.1	-2.576252051	0.117031255	Under expressed
XM_012190496.3	-2.575766841	0.000110181	Under expressed
XM_027965056.1	-2.57332503	0.109677011	Under expressed
XM_027977661.1	-2.571140059	0.014994054	Under expressed
XM_027965128.1	-2.569759366	0.043256975	Under expressed
XM_027976892.1	-2.568835793	0.000782958	Under expressed
NM_001009293.1	-2.565541926	0.049760155	Under expressed
XM_027964840.1	-2.563483942	0.002404799	Under expressed
NM_001246209.1	-2.559845038	0.129296313	Under expressed
XM_027977103.1	-2.557261932	4.80E-05	Under expressed
XM_004005642.4	-2.551554007	0.149437976	Under expressed
XM_012096909.2	-2.551344676	0.08353401	Under expressed
XM_027959102.1	-2.550782828	0.115316184	Under expressed
XM_027964937.1	-2.547901367	0.07653042	Under expressed
XM_012124958.3	-2.54756548	0.056472868	Under expressed
XM_015096203.2	-2.54563585	0.029615382	Under expressed
XR_003588162.1	-2.543968284	0.141928052	Under expressed
XM_004016068.4	-2.54164378	0.005145597	Under expressed
XM_012103494.3	-2.540907682	0.064610026	Under expressed
XM_004009930.3	-2.537866117	0.000492242	Under expressed
XM_027960720.1	-2.535439142	0.001165931	Under expressed
XM_012178991.3	-2.534524484	0.029903906	Under expressed
XM_027973573.1	-2.533904354	0.000643223	Under expressed
XM_004013634.4	-2.533561962	0.005613327	Under expressed
XM_027959347.1	-2.532386572	0.018614384	Under expressed
XM_004015503.3	-2.532229707	0.115627782	Under expressed
XM_027979709.1	-2.530862531	0.135150578	Under expressed
XM_004011372.4	-2.53085244	0.046968184	Under expressed
XM_004020238.4	-2.523287902	0.012394268	Under expressed
XM_027978698.1	-2.522024073	0.084596675	Under expressed
XR_003590978.1	-2.519186034	0.050091499	Under expressed
XM_015099445.2	-2.518705595	0.088637851	Under expressed
XM_012100566.3	-2.517785349	0.136751216	Under expressed
XR_001042902.2	-2.517630772	4.19E-08	Under expressed
XM_027959263.1	-2.51658205	0.028011135	Under expressed
XM_004021485.4	-2.516540719	0.004889329	Under expressed
XM_012184136.2	-2.513320107	0.001485509	Under expressed
XM_004008623.3	-2.509477628	0.141062276	Under expressed
XM_004016542.3	-2.505109905	0.048918956	Under expressed
XM_012175197.2	-2.502373304	0.006047766	Under expressed

XM_015093381.2	-2.502073819	0.028191205	Under expressed
XM_027973013.1	-2.495152241	0.050693452	Under expressed
XM_027964900.1	-2.494066858	0.137768563	Under expressed
XM_012133312.3	-2.490122276	0.075594242	Under expressed
XM_027978926.1	-2.48837903	0.005715957	Under expressed
XM_027971117.1	-2.487832742	0.038337281	Under expressed
XR_003587350.1	-2.48781016	0.081403978	Under expressed
XM_027965401.1	-2.486371588	0.078578143	Under expressed
XM_027979939.1	-2.48497606	0.140400335	Under expressed
XM_004022208.4	-2.481191298	0.024808047	Under expressed
XM_004006895.3	-2.478677056	0.064928596	Under expressed
XM_015104100.2	-2.475064107	0.036268187	Under expressed
XM_012101125.3	-2.47415569	0.038722969	Under expressed
XM_027966866.1	-2.473769151	0.06649804	Under expressed
XM_015097743.2	-2.472997011	0.005503951	Under expressed
XM_004010606.4	-2.465773877	0.010320022	Under expressed
XM_027964209.1	-2.465360775	0.012061769	Under expressed
XM_027967244.1	-2.463793407	0.087415582	Under expressed
XM_004018341.4	-2.463258808	0.000381695	Under expressed
XM_027960636.1	-2.460015215	0.066638332	Under expressed
XR_003586536.1	-2.459764397	0.009134949	Under expressed
NM_001127283.1	-2.45916185	0.023347857	Under expressed
NM_001306117.1	-2.45646364	0.073745649	Under expressed
XM_015094763.2	-2.452859578	0.059532632	Under expressed
XR_003586846.1	-2.450216351	0.117603829	Under expressed
XM_004011849.3	-2.450191269	0.000956676	Under expressed
XM_015095181.2	-2.443105471	0.092045171	Under expressed
XM_027971763.1	-2.431926928	0.139877251	Under expressed
XR_001435971.2	-2.431907498	0.023634219	Under expressed
XM_004022011.3	-2.430712867	0.003488937	Under expressed
XM_027960494.1	-2.428901337	0.004195614	Under expressed
XM_012130478.3	-2.427518124	0.141928052	Under expressed
XM_004001961.4	-2.426864331	0.024990657	Under expressed
XR_003590462.1	-2.424942916	0.04154997	Under expressed
XM_027960253.1	-2.423112156	0.038352983	Under expressed
XM_004022470.3	-2.421557382	0.117273093	Under expressed
XM_027978697.1	-2.419811757	0.001174062	Under expressed
XM_012107035.3	-2.41601862	0.004256588	Under expressed
XM_027971803.1	-2.414924264	0.004984436	Under expressed
XM_012181318.2	-2.411667044	0.096594072	Under expressed
XM_027959293.1	-2.4051698	0.114061303	Under expressed
XR_003586255.1	-2.404059904	0.059741331	Under expressed
XM_015093054.2	-2.398580493	0.006077946	Under expressed
XM_015097210.2	-2.398132616	0.031871959	Under expressed

XM_027963003.1	-2.39064164	0.010012584	Under expressed
XM_004012195.4	-2.389045758	0.000174424	Under expressed
XM_027964780.1	-2.385378693	0.054527649	Under expressed
XM_012102986.3	-2.385232758	0.117761176	Under expressed
XM_012178936.3	-2.385017532	0.049857534	Under expressed
XM_027976590.1	-2.383273933	0.11984936	Under expressed
XM_027957447.1	-2.380196168	0.070174815	Under expressed
NM_001281825.1	-2.377271739	0.01120223	Under expressed
XM_004007842.4	-2.376090069	0.045213358	Under expressed
XM_004022371.4	-2.374061821	0.140366915	Under expressed
XM_027971634.1	-2.37403585	0.104239894	Under expressed
XM_027972436.1	-2.372591532	0.018120173	Under expressed
XM_012097061.2	-2.371985907	0.05098375	Under expressed
XM_027976185.1	-2.37114257	0.147752049	Under expressed
XM_015095934.2	-2.370361549	0.023409628	Under expressed
XR_003590728.1	-2.368259563	0.003484533	Under expressed
XM_004020480.4	-2.368222412	0.028158243	Under expressed
NM_001195318.1	-2.364195541	0.000648606	Under expressed
XM_012096363.3	-2.362614897	0.03158679	Under expressed
XM_012174395.3	-2.362287933	2.16E-05	Under expressed
XM_004009673.4	-2.361020977	0.00186862	Under expressed
XM_027975389.1	-2.358617949	0.05514832	Under expressed
XM_004014191.4	-2.357446215	0.002012264	Under expressed
XM_004017018.4	-2.356606461	0.012505497	Under expressed
XM_015102838.2	-2.354925111	0.020982754	Under expressed
XM_004006498.4	-2.351273062	0.010351125	Under expressed
XM_012174145.3	-2.351070441	0.107697796	Under expressed
NM_001162551.1	-2.347615242	0.099010274	Under expressed
NM_001143892.1	-2.347539835	0.013815031	Under expressed
NM_001280672.1	-2.346654725	0.04070628	Under expressed
XM_027972954.1	-2.345749554	0.056759396	Under expressed
XM_015096973.2	-2.339706237	0.133584439	Under expressed
XM_004002337.4	-2.339304511	0.060794449	Under expressed
XM_027972216.1	-2.3357603	0.063445055	Under expressed
XM_004005927.4	-2.334642184	0.005107369	Under expressed
XR_003588734.1	-2.333417854	0.017914835	Under expressed
XM_027980051.1	-2.33220138	0.011935602	Under expressed
XM_004020189.4	-2.330880327	0.135150578	Under expressed
XM_027960308.1	-2.327891639	0.148620498	Under expressed
XM_027970531.1	-2.32775094	0.006415198	Under expressed
XM_027973705.1	-2.323845511	0.080147962	Under expressed
XM_027965132.1	-2.321973489	0.055677671	Under expressed
XR_003589742.1	-2.319832763	0.115360518	Under expressed
XM_012105052.3	-2.312705224	0.005011484	Under expressed

XM_004002179.4	-2.30939893	0.007216024	Under expressed
XM_027974854.1	-2.307409181	0.126095431	Under expressed
XR_003586495.1	-2.301711011	0.044867291	Under expressed
XM_012137392.2	-2.300921124	0.002442899	Under expressed
XM_027960936.1	-2.299914241	0.00329434	Under expressed
XM_027969891.1	-2.298540717	0.084035725	Under expressed
XM_027962755.1	-2.29740851	0.137337678	Under expressed
XM_027959724.1	-2.295100649	0.068403482	Under expressed
XM_027966193.1	-2.288014943	0.000710836	Under expressed
XM_004002710.3	-2.28714055	0.006150103	Under expressed
XM_004003336.4	-2.286322203	0.013972216	Under expressed
XM_027957526.1	-2.279087073	0.114734629	Under expressed
NM_001009803.1	-2.277133115	0.129354138	Under expressed
XM_027966825.1	-2.276782202	0.002301445	Under expressed
XM_012185866.3	-2.275846753	0.018430334	Under expressed
XM_027966972.1	-2.274507074	0.037413702	Under expressed
XM_012106260.3	-2.274328442	0.042329928	Under expressed
XM_012184450.2	-2.27286809	0.094967488	Under expressed
XM_027970941.1	-2.269567718	0.011309071	Under expressed
XM_015092360.2	-2.269414031	0.069792492	Under expressed
XM_004015202.4	-2.263944431	0.011966257	Under expressed
XM_012095475.3	-2.263941228	9.92E-09	Under expressed
XM_004002746.4	-2.262489272	0.007253212	Under expressed
XR_001044485.3	-2.261731954	0.055980039	Under expressed
XM_027964909.1	-2.26170111	0.125073826	Under expressed
XM_004008031.4	-2.258222979	0.002033644	Under expressed
XM_015094597.2	-2.257421744	0.031471739	Under expressed
XM_015096188.2	-2.24944231	0.062892863	Under expressed
XM_004018246.4	-2.249037331	0.071936565	Under expressed
XM_027979572.1	-2.248474978	0.007402277	Under expressed
XM_004018862.4	-2.247213598	0.012995781	Under expressed
XM_027975557.1	-2.247002818	0.041056259	Under expressed
NM_001162546.1	-2.246198509	0.043394599	Under expressed
XM_004008807.4	-2.244944193	0.027866223	Under expressed
XM_027971375.1	-2.244555943	0.013130177	Under expressed
XM_004005657.4	-2.241439329	0.001937031	Under expressed
XM_015092367.2	-2.241027412	0.001138348	Under expressed
XM_027973702.1	-2.240874177	0.14598292	Under expressed
XM_027973562.1	-2.239639619	0.006894064	Under expressed
XM_027973524.1	-2.238505922	0.027657722	Under expressed
XR_003590249.1	-2.237902755	0.005239709	Under expressed
XM_027978933.1	-2.235129895	0.143047857	Under expressed
XM_027960554.1	-2.232024773	0.060101614	Under expressed
XM_015104559.2	-2.230529725	0.046858452	Under expressed

XM_027966723.1	-2.229110935	0.005533585	Under expressed
XM_004004031.4	-2.228227515	0.085845743	Under expressed
XM_015092049.2	-2.227130706	0.02458214	Under expressed
XM_004002484.4	-2.226087455	0.012216998	Under expressed
XM_004021939.4	-2.221936074	0.008270735	Under expressed
XM_027972310.1	-2.220241348	0.067325675	Under expressed
XM_012181770.3	-2.212182028	0.001216114	Under expressed
XM_015096775.2	-2.210833199	0.000194455	Under expressed
XM_027970867.1	-2.210346365	0.041863402	Under expressed
XM_012184971.3	-2.208427253	0.044466842	Under expressed
XM_004017922.3	-2.207358676	0.000153308	Under expressed
XR_003585923.1	-2.20210179	0.062180562	Under expressed
XR_003591490.1	-2.198576009	0.005364164	Under expressed
XM_027974335.1	-2.198041703	0.028801284	Under expressed
XM_027975992.1	-2.197867613	0.004348272	Under expressed
XM_027979334.1	-2.19487977	0.007310305	Under expressed
XM_027979607.1	-2.194859427	0.08218688	Under expressed
XM_027967229.1	-2.194802081	0.08501799	Under expressed
XM_027962302.1	-2.189688338	0.056582848	Under expressed
XM_004014303.4	-2.188537991	0.000153257	Under expressed
XM_015091599.2	-2.187341811	0.035136979	Under expressed
XM_004017830.3	-2.184878215	0.014194453	Under expressed
XM_004022471.4	-2.183125172	5.13E-05	Under expressed
XM_012178334.3	-2.182827129	0.038357434	Under expressed
NM_001163598.1	-2.178208466	0.008701424	Under expressed
XR_003588722.1	-2.176711409	0.001554644	Under expressed
XM_004009926.4	-2.176261527	2.50E-06	Under expressed
XM_027963731.1	-2.176166818	0.038816282	Under expressed
XM_004020009.4	-2.175797748	0.029498492	Under expressed
XM_027964017.1	-2.174722167	0.084608164	Under expressed
XM_004016167.3	-2.174250034	0.006836244	Under expressed
XM_027968500.1	-2.173879376	0.007131137	Under expressed
XM_027977324.1	-2.171762994	0.127289261	Under expressed
XM_027973275.1	-2.170461862	0.007970897	Under expressed
XR_003588553.1	-2.168699018	7.27E-09	Under expressed
XM_027964730.1	-2.167934654	0.063294169	Under expressed
XM_015097395.2	-2.167699421	0.001183007	Under expressed
XM_004002866.3	-2.167589531	0.133441161	Under expressed
XM_027971984.1	-2.167475282	0.018737395	Under expressed
XR_003588888.1	-2.163539599	0.032699088	Under expressed
XM_027967158.1	-2.162320662	0.073304131	Under expressed
XM_027962854.1	-2.162277848	0.015126033	Under expressed
XM_015092418.2	-2.160250858	0.027941289	Under expressed
XM_027967784.1	-2.159526746	0.051378708	Under expressed

XM_004016872.3	-2.159032308	0.0859512	Under expressed
XM_012097270.3	-2.156658804	0.009807799	Under expressed
XM_004013898.3	-2.156229215	0.04085217	Under expressed
XM_004022220.4	-2.156137226	0.028191205	Under expressed
XR_003586541.1	-2.154575715	0.022091556	Under expressed
XM_015096231.2	-2.153954464	0.012490613	Under expressed
XM_027956292.1	-2.150993509	0.122105756	Under expressed
XM_004015199.4	-2.150287684	0.059634063	Under expressed
XM_004009621.3	-2.150221801	0.084118225	Under expressed
XM_012180719.3	-2.14774891	0.006553512	Under expressed
XM_004014912.4	-2.145243931	6.65E-05	Under expressed
XM_027964707.1	-2.143767822	0.093264292	Under expressed
XM_015092136.2	-2.140858841	0.000276847	Under expressed
XM_015092054.2	-2.139773271	0.023609912	Under expressed
XM_027980137.1	-2.139201453	0.001506081	Under expressed
NM_001126361.1	-2.138081182	0.144611121	Under expressed
XM_027962669.1	-2.137523474	0.025010063	Under expressed
XM_027968476.1	-2.136021735	0.011309071	Under expressed
XM_015098436.2	-2.130947462	0.060691503	Under expressed
XM_027971483.1	-2.130641023	0.024405786	Under expressed
XM_012135959.3	-2.130267372	0.057673062	Under expressed
XM_027956992.1	-2.130002474	0.082649792	Under expressed
XM_004009709.4	-2.127578043	0.014393813	Under expressed
XM_012184327.3	-2.125835826	0.027563549	Under expressed
XM_012185228.3	-2.125697275	0.021320172	Under expressed
XM_027962303.1	-2.124851674	0.00090434	Under expressed
XR_003591761.1	-2.11857493	0.137815679	Under expressed
XM_027956323.1	-2.117911263	0.021755749	Under expressed
XM_027964951.1	-2.114882579	0.101002209	Under expressed
XM_027975590.1	-2.111777062	0.06435357	Under expressed
XM_012175401.3	-2.111138797	7.97E-06	Under expressed
XM_004014973.3	-2.11043558	0.10963996	Under expressed
XM_027980075.1	-2.109439342	0.120210216	Under expressed
XR_003588225.1	-2.106508872	0.115228927	Under expressed
XM_027978673.1	-2.105427196	2.77E-05	Under expressed
XM_004020249.3	-2.10423698	0.099883603	Under expressed
NM_001009254.1	-2.104232969	0.090169349	Under expressed
XM_027969196.1	-2.103628535	0.088637851	Under expressed
XR_003590932.1	-2.103335801	0.064733848	Under expressed
XM_012107255.3	-2.102224728	0.00749432	Under expressed
XM_027971453.1	-2.101771081	0.114292557	Under expressed
XM_027978705.1	-2.101290318	0.09134946	Under expressed
XM_027971441.1	-2.100477223	0.001136631	Under expressed
NM_001161886.1	-2.099499943	0.08218688	Under expressed

XM_004008805.4	-2.09653593	0.090253103	Under expressed
XM_027973243.1	-2.094851961	0.005255699	Under expressed
XM_004004405.4	-2.094654552	0.002069099	Under expressed
XM_027957240.1	-2.092909791	0.012525433	Under expressed
NM_001246211.1	-2.088787092	0.058471209	Under expressed
XM_027965926.1	-2.087029862	0.057072535	Under expressed
XM_004010590.4	-2.085775529	0.004627867	Under expressed
XM_015093280.2	-2.0854582	0.000350677	Under expressed
XM_004005110.4	-2.085274729	0.143047857	Under expressed
XM_027972489.1	-2.085119565	0.013794946	Under expressed
XM_004021055.4	-2.084823577	0.068067799	Under expressed
XM_012106120.3	-2.083797949	0.084546366	Under expressed
XM_027970521.1	-2.082214808	0.000280416	Under expressed
XM_027975137.1	-2.081748292	0.144632422	Under expressed
XM_004017823.4	-2.081514653	0.00186862	Under expressed
XM_004010658.3	-2.081459398	0.050091499	Under expressed
XM_012189202.3	-2.0793193	0.053799132	Under expressed
XM_027967993.1	-2.078913142	0.084608164	Under expressed
XM_012106516.2	-2.078567414	0.034812776	Under expressed
XR_003590878.1	-2.077932612	0.03659084	Under expressed
XM_004013845.4	-2.076478372	0.08686471	Under expressed
XM_004003000.4	-2.075412921	0.027325012	Under expressed
XR_003590690.1	-2.074137353	0.009954469	Under expressed
XR_001434185.2	-2.074053553	0.058173522	Under expressed
XM_027979842.1	-2.071587874	0.054004676	Under expressed
XM_027977757.1	-2.070430118	0.084035725	Under expressed
XM_027959181.1	-2.068356739	0.033667572	Under expressed
XM_004004303.4	-2.068114022	0.05421097	Under expressed
XM_027979676.1	-2.06807212	0.084518502	Under expressed
XM_012105225.3	-2.06545521	0.053033863	Under expressed
XM_015104004.2	-2.065185848	0.006950817	Under expressed
XM_004007771.4	-2.064810928	0.051900231	Under expressed
XM_012176847.2	-2.061962115	0.046227553	Under expressed
XM_027971060.1	-2.061404175	0.000533767	Under expressed
XM_004010508.4	-2.060105738	0.025638898	Under expressed
XM_027971779.1	-2.053806007	0.041635347	Under expressed
XR_003591117.1	-2.049257043	0.046917179	Under expressed
XM_027959053.1	-2.046302952	0.045641359	Under expressed
XM_027970431.1	-2.045365377	0.114276131	Under expressed
XM_012103738.2	-2.04480283	2.85E-05	Under expressed
XM_004022399.4	-2.043277074	0.004295673	Under expressed
XM_015100903.2	-2.043126942	0.069279782	Under expressed
XM_027966551.1	-2.041157891	0.075594242	Under expressed
NM_001142887.1	-2.040472886	0.11898354	Under expressed

XR_003588767.1	-2.039039233	0.057244981	Under expressed
XM_027956615.1	-2.038906457	0.008681624	Under expressed
XR_003591442.1	-2.038409327	0.061010636	Under expressed
XM_012178826.3	-2.03645071	0.000691041	Under expressed
XR_003590278.1	-2.036312314	0.00137551	Under expressed
XR_003589965.1	-2.034866891	0.025112993	Under expressed
XM_004011832.4	-2.031853658	0.057460464	Under expressed
XM_015104390.2	-2.031026008	0.001136631	Under expressed
XM_004004570.4	-2.030954847	2.85E-05	Under expressed
XM_012178515.2	-2.030797326	0.020781231	Under expressed
XM_015102064.2	-2.030757046	0.022914715	Under expressed
XM_004002204.4	-2.030001405	0.003647472	Under expressed
XM_015099353.2	-2.028908337	0.004225515	Under expressed
XM_012099452.2	-2.02888926	0.082227618	Under expressed
XR_003586086.1	-2.028484459	0.024037753	Under expressed
XM_012097548.3	-2.028168936	0.055871747	Under expressed
XM_004021751.3	-2.027460995	0.026396168	Under expressed
XM_004020483.3	-2.027080552	0.031871959	Under expressed
XM_004008531.4	-2.025741915	0.021828034	Under expressed
XM_004020948.4	-2.02496211	0.042807627	Under expressed
XM_027968911.1	-2.024574925	0.020798222	Under expressed
XM_012097525.3	-2.024405763	0.081286604	Under expressed
XR_003589890.1	-2.017598802	0.003978605	Under expressed
XM_012095290.3	-2.017444899	0.005852382	Under expressed
XM_004007845.4	-2.017114274	0.038453148	Under expressed
XM_027967641.1	-2.01706382	0.030579045	Under expressed
XM_015094497.2	-2.015932076	0.001892719	Under expressed
XM_027975823.1	-2.010675639	0.049734581	Under expressed
XR_001435858.2	-2.010178758	0.137278671	Under expressed
XM_027972993.1	-2.008972923	0.021565867	Under expressed
XM_004006692.4	-2.004798215	0.069728327	Under expressed
XM_027972451.1	-2.004741194	0.075594242	Under expressed
XM_004016320.4	-2.004629151	0.105599617	Under expressed
XM_027973858.1	-2.003914014	0.095612043	Under expressed
XM_027976262.1	-2.003750788	0.111168567	Under expressed
XM_015093555.2	-2.000220867	0.004225515	Under expressed
XM_027964788.1	-2.000153889	0.050282539	Under expressed
XM_004011263.4	-1.998194479	0.003701093	Under expressed
XM_015097459.2	-1.997778345	0.016932605	Under expressed
XM_012176385.3	-1.996878352	0.042527124	Under expressed
XM_027958726.1	-1.9962586	0.010789577	Under expressed
XM_004012102.3	-1.995816454	0.070406603	Under expressed
XM_004009919.4	-1.995262212	0.043200834	Under expressed
XM_027970107.1	-1.994947725	0.054004676	Under expressed

XM_027979741.1	-1.993759447	0.116020421	Under expressed
XM_015097744.2	-1.993640667	0.000515334	Under expressed
XM_012184629.3	-1.991933146	0.002546516	Under expressed
XM_004004353.4	-1.991637946	0.016999286	Under expressed
XM_027964049.1	-1.985413087	0.11401293	Under expressed
XM_027963265.1	-1.982195841	0.06263427	Under expressed
XR_001039255.3	-1.982092238	0.028923781	Under expressed
XM_027967884.1	-1.980892186	0.130367032	Under expressed
XM_027968589.1	-1.977926482	0.027908835	Under expressed
XM_015100374.2	-1.977232256	0.04289252	Under expressed
XM_004008850.3	-1.975772058	0.022211972	Under expressed
XM_027979630.1	-1.974405878	0.085754993	Under expressed
XM_004005901.4	-1.972417549	8.63E-05	Under expressed
XM_027967631.1	-1.969484324	0.102270871	Under expressed
NM_001130154.1	-1.969403587	0.080817572	Under expressed
XM_012097712.3	-1.96882909	0.025194359	Under expressed
XM_004007846.4	-1.966800083	0.005988437	Under expressed
XR_003588674.1	-1.966035865	0.007777481	Under expressed
XM_015095306.2	-1.9640472	0.000100237	Under expressed
XM_027962434.1	-1.960538325	0.126330161	Under expressed
XM_004011193.4	-1.959121117	0.008669331	Under expressed
XM_015103722.2	-1.95787763	0.005533585	Under expressed
XR_003586529.1	-1.95573989	0.028669153	Under expressed
XM_027973160.1	-1.955697094	0.036201361	Under expressed
XM_015094292.2	-1.954187501	0.034465591	Under expressed
XM_004002269.4	-1.952829019	0.141928052	Under expressed
XM_027976360.1	-1.952291149	0.084035725	Under expressed
XM_027961089.1	-1.952217104	0.015348257	Under expressed
XM_004002839.4	-1.951788943	0.017559004	Under expressed
XM_012105740.2	-1.950116684	0.031672061	Under expressed
XM_004012421.4	-1.948488154	0.116914815	Under expressed
XM_012183375.2	-1.947320706	8.33E-06	Under expressed
XM_004010489.4	-1.947021276	0.01966536	Under expressed
XM_004017028.4	-1.945523242	0.029296021	Under expressed
XM_027968871.1	-1.944854331	0.058398228	Under expressed
NM_001009394.1	-1.944503511	0.010514345	Under expressed
XM_027956247.1	-1.943753403	0.001099951	Under expressed
XM_027968213.1	-1.943430562	0.008154444	Under expressed
NM_001009742.1	-1.943014685	0.02352861	Under expressed
XM_004002165.4	-1.941629508	0.120346349	Under expressed
XM_004003007.3	-1.94062315	0.077415236	Under expressed
XM_012176761.3	-1.932522086	0.068970619	Under expressed
XM_004016298.4	-1.931669559	0.05098375	Under expressed
XR_003590497.1	-1.929919318	0.091844416	Under expressed

XM_004007817.4	-1.926948493	0.043073883	Under expressed
XM_015100531.2	-1.926507126	0.022689947	Under expressed
XM_012105588.2	-1.925812133	6.84E-07	Under expressed
XM_012097004.2	-1.925277943	0.037190462	Under expressed
XR_003586535.1	-1.924133392	0.013130177	Under expressed
XM_004017070.3	-1.922705583	0.047604785	Under expressed
XM_027971121.1	-1.922436026	0.003791834	Under expressed
NM_001127274.1	-1.92180693	0.026656617	Under expressed
XM_004003873.4	-1.921002286	0.001259681	Under expressed
XM_015093334.2	-1.920075551	0.118156747	Under expressed
XM_027977955.1	-1.915709862	0.028168979	Under expressed
XM_004017712.4	-1.915310988	0.002626774	Under expressed
XM_004012516.4	-1.914333304	0.102275821	Under expressed
XM_015096173.2	-1.914221709	0.046072859	Under expressed
XM_012110914.3	-1.913295302	0.087008378	Under expressed
XM_027979016.1	-1.912513681	0.065042304	Under expressed
XM_004020252.4	-1.910296936	0.075299708	Under expressed
XM_027972945.1	-1.907769778	0.006560197	Under expressed
XM_027970888.1	-1.906525523	0.077231286	Under expressed
XM_027977799.1	-1.905986266	5.88E-14	Under expressed
XM_012188639.3	-1.90572698	0.025890047	Under expressed
XM_004018269.3	-1.90497814	0.071250516	Under expressed
XM_015099670.2	-1.904322131	0.032140067	Under expressed
XM_027966867.1	-1.904041665	0.042704429	Under expressed
XR_003590605.1	-1.902496781	0.146132376	Under expressed
XM_004007807.4	-1.901104529	0.065042304	Under expressed
XM_004004354.4	-1.897987203	0.091187943	Under expressed
XR_003591068.1	-1.897281892	0.03417323	Under expressed
XM_004020155.4	-1.896756406	0.115831515	Under expressed
XM_027978929.1	-1.896583656	0.057575546	Under expressed
XR_003586698.1	-1.894136793	7.84E-06	Under expressed
XM_027972871.1	-1.893165937	0.128605827	Under expressed
XM_004010459.4	-1.891095533	0.064423171	Under expressed
XM_027966243.1	-1.890074815	0.007192831	Under expressed
XM_027962719.1	-1.889938863	0.090582644	Under expressed
XM_027975271.1	-1.887831867	0.076207539	Under expressed
XM_004013651.4	-1.885535513	0.032397353	Under expressed
XM_027973501.1	-1.885048813	0.02136222	Under expressed
XM_004001925.4	-1.882626596	0.14474187	Under expressed
XM_012097320.3	-1.881399955	0.114734629	Under expressed
XM_012187092.3	-1.880400012	0.037180511	Under expressed
NM_001035124.1	-1.879558405	0.148846638	Under expressed
XM_027979524.1	-1.879518288	0.057685909	Under expressed
XM_004004522.3	-1.879407077	0.025086922	Under expressed

XM_012190225.3	-1.878241788	1.33E-06	Under expressed
XM_027959198.1	-1.878187838	0.003057442	Under expressed
XM_015102456.2	-1.876257029	0.002536323	Under expressed
NM_001104929.1	-1.875362011	0.073189527	Under expressed
XR_003587291.1	-1.873512304	0.000979204	Under expressed
XM_027971825.1	-1.87344563	0.097078287	Under expressed
XM_027964538.1	-1.873066262	0.060480173	Under expressed
XM_027963094.1	-1.872826653	0.005897738	Under expressed
XM_027964704.1	-1.871613732	0.089254162	Under expressed
XM_027974154.1	-1.871401976	0.117748156	Under expressed
XM_012175410.3	-1.869626659	0.00652663	Under expressed
XM_012176887.2	-1.867291278	0.020180848	Under expressed
NM_001166191.1	-1.867241188	0.007216024	Under expressed
XM_027961745.1	-1.866075394	0.032574721	Under expressed
XM_027959563.1	-1.862405836	0.147752049	Under expressed
XM_004016392.4	-1.862238759	0.003150734	Under expressed
XM_015093087.2	-1.861706896	0.096013641	Under expressed
XM_004009450.4	-1.857931703	0.0500956	Under expressed
XM_027974632.1	-1.857256533	0.034011567	Under expressed
XM_027973548.1	-1.855682729	0.025501467	Under expressed
XM_004014976.4	-1.855662666	0.114196429	Under expressed
XM_027958202.1	-1.853459461	0.049800864	Under expressed
XM_004016983.4	-1.853405618	0.023161265	Under expressed
XR_003588876.1	-1.852816755	0.006141227	Under expressed
XM_004006025.4	-1.851689404	0.009939424	Under expressed
XM_012166271.3	-1.850946634	0.002236757	Under expressed
XM_004004520.3	-1.848936047	0.093062156	Under expressed
XM_004010646.4	-1.84785194	0.00736327	Under expressed
XM_004010662.4	-1.84702209	0.000509026	Under expressed
XM_012188287.3	-1.846789453	0.115713742	Under expressed
XM_027970915.1	-1.846711912	5.79E-05	Under expressed
XM_004022345.3	-1.84416346	0.035935522	Under expressed
XM_012181288.3	-1.841608675	0.049948168	Under expressed
XM_004009683.4	-1.840460827	0.116757225	Under expressed
XM_027973693.1	-1.840372048	0.056472868	Under expressed
XM_012123470.3	-1.839700392	0.013559821	Under expressed
XM_015093369.2	-1.838465758	0.008545722	Under expressed
XM_004002203.4	-1.837943065	0.044117137	Under expressed
XM_015094428.2	-1.836982092	5.34E-06	Under expressed
XM_004008102.3	-1.83661296	0.006558568	Under expressed
XM_027961765.1	-1.835548586	0.024990657	Under expressed
XM_004004364.4	-1.835249683	0.016004599	Under expressed
XM_027978921.1	-1.834726724	0.01953568	Under expressed
XM_012174832.3	-1.832914883	0.014883772	Under expressed

XM_004016168.4	-1.828010309	0.120597537	Under expressed
XM_027956264.1	-1.827617116	0.111083299	Under expressed
XM_004010481.4	-1.826338064	0.016767734	Under expressed
XM_004013756.4	-1.82427591	0.127289533	Under expressed
XM_004013018.4	-1.820983364	0.007139639	Under expressed
XM_004014916.4	-1.818179997	0.09252059	Under expressed
XM_012095379.3	-1.818016858	0.004224938	Under expressed
XM_004002229.4	-1.816842398	0.039306344	Under expressed
XM_004011129.3	-1.814860381	0.075520292	Under expressed
XR_001434709.2	-1.814380181	0.002350597	Under expressed
XM_012101700.3	-1.813424615	0.046931213	Under expressed
XM_004020195.4	-1.81328828	0.008616488	Under expressed
XM_027961881.1	-1.812103738	0.034075311	Under expressed
XM_004006796.4	-1.809456779	0.126743729	Under expressed
XM_004020200.4	-1.808728036	0.086048531	Under expressed
XM_004014312.4	-1.808123003	0.042463431	Under expressed
XR_001042183.3	-1.807733674	0.001485509	Under expressed
XM_027975151.1	-1.807714652	0.033731472	Under expressed
XM_004013732.4	-1.807478383	0.137884717	Under expressed
XR_003587321.1	-1.806831848	1.21E-08	Under expressed
XM_027962135.1	-1.805970101	0.0301931	Under expressed
XM_015094881.2	-1.805081659	0.119883856	Under expressed
XM_027970985.1	-1.803644046	0.01179945	Under expressed
XM_027964543.1	-1.803340817	0.002427433	Under expressed
XM_012097392.2	-1.802816609	1.89E-08	Under expressed
XM_027972135.1	-1.802810864	0.147101445	Under expressed
XM_027965897.1	-1.802278204	0.001802194	Under expressed
NM_001025110.1	-1.801314249	8.11E-05	Under expressed
XM_015098590.2	-1.801282631	0.000473384	Under expressed
XM_004010428.4	-1.799887316	0.048674569	Under expressed
XM_004004454.4	-1.79969687	0.011951962	Under expressed
XM_015101936.2	-1.799481058	0.000478565	Under expressed
XM_027979981.1	-1.799289691	0.028376806	Under expressed
XM_004018337.4	-1.798875838	0.094592043	Under expressed
XM_004012098.4	-1.79305722	0.030144416	Under expressed
XM_004012827.4	-1.792186284	0.01724588	Under expressed
XM_015104633.2	-1.788950995	0.030611625	Under expressed
XM_015100149.2	-1.788858934	0.102443706	Under expressed
XM_015103685.2	-1.788466111	0.130812146	Under expressed
XM_015096628.2	-1.78764619	0.138021514	Under expressed
XM_027956254.1	-1.787379599	0.007348011	Under expressed
XM_027979151.1	-1.78726536	0.017406573	Under expressed
XR_003587200.1	-1.786600198	0.008768154	Under expressed
XM_004006496.4	-1.786511643	0.018172911	Under expressed

XM_015096439.2	-1.784837706	0.046533231	Under expressed
XM_027970039.1	-1.783202933	0.026567364	Under expressed
XM_027970755.1	-1.783112731	0.024578058	Under expressed
XM_012182449.3	-1.781875321	4.06E-05	Under expressed
XM_015099702.2	-1.780972858	0.021320172	Under expressed
XM_027978431.1	-1.777643653	0.038845458	Under expressed
XM_004004106.4	-1.776434565	0.017165079	Under expressed
XM_027976278.1	-1.774968014	0.019558062	Under expressed
XM_015094459.2	-1.769710055	0.020033532	Under expressed
XM_027978669.1	-1.769224088	0.008452279	Under expressed
XM_004016887.4	-1.769036863	0.043089562	Under expressed
XM_004015460.4	-1.768824202	0.030997269	Under expressed
XR_003586942.1	-1.768205416	0.002123212	Under expressed
XM_012159435.3	-1.768203586	0.109608148	Under expressed
XM_004014564.4	-1.768055276	0.017091618	Under expressed
XR_003588738.1	-1.767986904	0.130933705	Under expressed
XM_004004736.4	-1.767729484	0.142857232	Under expressed
XM_004019457.3	-1.764108151	0.078383124	Under expressed
XM_004020476.4	-1.764050057	0.026898471	Under expressed
XM_004014278.4	-1.762911452	0.039631606	Under expressed
XM_004012146.4	-1.762883792	0.00652663	Under expressed
NM_001287465.1	-1.762771472	0.042329928	Under expressed
XM_015091971.2	-1.76089872	0.025252024	Under expressed
XM_012106891.3	-1.759819141	0.037456646	Under expressed
XM_015103679.2	-1.759412877	0.002048159	Under expressed
XM_004018876.4	-1.758738886	0.093839059	Under expressed
XM_004006684.4	-1.756842094	0.115140466	Under expressed
XM_004016996.4	-1.753063479	0.000494498	Under expressed
XM_027961326.1	-1.752480213	0.005046768	Under expressed
XM_027964562.1	-1.752352204	0.023199743	Under expressed
XM_004010216.4	-1.75220689	0.051021572	Under expressed
XM_004004368.4	-1.751822211	0.078188975	Under expressed
XM_027971886.1	-1.751424602	0.088730558	Under expressed
XM_004005728.3	-1.750320449	0.101379457	Under expressed
XM_004004259.4	-1.748180189	0.00372592	Under expressed
XM_012187720.3	-1.74772553	0.002440398	Under expressed
XM_012182826.3	-1.746705901	0.010179723	Under expressed
XM_027963792.1	-1.740913006	0.002820809	Under expressed
XM_004012838.4	-1.740821776	0.0001793	Under expressed
XM_027961708.1	-1.739976606	0.133159195	Under expressed
XM_015096447.2	-1.73988194	0.027480359	Under expressed
XM_027968174.1	-1.738559107	0.075246904	Under expressed
XM_027967732.1	-1.736341144	0.044142776	Under expressed
XR_003589868.1	-1.734167111	0.035934028	Under expressed

XM_004016121.4	-1.733620005	0.073591388	Under expressed
XM_004002835.4	-1.73339006	0.114734629	Under expressed
XM_004007878.3	-1.733356866	0.00027703	Under expressed
XM_004014623.4	-1.731457899	0.028342289	Under expressed
XM_015096041.2	-1.731215497	0.037329918	Under expressed
XM_004006399.4	-1.731112119	0.134084441	Under expressed
XM_004015118.4	-1.730734123	0.024739644	Under expressed
XM_015102332.2	-1.730366623	0.062853708	Under expressed
XM_004010550.4	-1.729189347	0.028727397	Under expressed
XM_015096230.2	-1.728782106	0.146388155	Under expressed
XM_004004465.4	-1.728775249	0.115767313	Under expressed
XM_004010233.3	-1.72724821	0.040192347	Under expressed
XR_003591185.1	-1.725547753	0.093173579	Under expressed
XM_004018846.4	-1.725191	0.015079196	Under expressed
XM_012188647.3	-1.725114366	0.13846551	Under expressed
XM_027979624.1	-1.723444196	0.116339591	Under expressed
XM_004010779.4	-1.723343885	0.021182952	Under expressed
XM_027973232.1	-1.723155662	0.000831219	Under expressed
XM_027963567.1	-1.722215274	0.099728554	Under expressed
XM_015092541.2	-1.720063322	0.098453336	Under expressed
XM_004016853.4	-1.716713135	0.001807768	Under expressed
XM_027971958.1	-1.715440396	0.115954368	Under expressed
XM_027975762.1	-1.715263434	0.012538451	Under expressed
XM_004016462.4	-1.714906077	0.012422835	Under expressed
XM_027971038.1	-1.714707629	0.014874463	Under expressed
XM_012175434.3	-1.714536213	0.144032633	Under expressed
XM_015104995.2	-1.714502479	0.048171702	Under expressed
XM_027964493.1	-1.713581322	0.000643223	Under expressed
XM_027972500.1	-1.713496564	0.062740582	Under expressed
XM_004012959.4	-1.713465504	0.00604876	Under expressed
XM_027970452.1	-1.713421038	0.149734613	Under expressed
XM_027960749.1	-1.713233093	0.060480173	Under expressed
XM_027972625.1	-1.708010202	0.048171702	Under expressed
XM_015104377.2	-1.707592568	0.018362321	Under expressed
XM_012127464.3	-1.707533133	0.055900731	Under expressed
XM_012186039.3	-1.705786872	0.045405495	Under expressed
XM_027976154.1	-1.704370922	0.06557683	Under expressed
XM_027964604.1	-1.704139583	0.043038278	Under expressed
XM_012110487.3	-1.703493055	0.002546553	Under expressed
XM_004005902.4	-1.701002066	0.113125338	Under expressed
XM_015093043.2	-1.700330689	0.008659495	Under expressed
XM_027965703.1	-1.699716473	0.049526479	Under expressed
XM_027963821.1	-1.699551031	0.013559821	Under expressed
XM_027975284.1	-1.69931368	0.120679402	Under expressed

XM_027972892.1	-1.699065045	0.046410217	Under expressed
XM_027964181.1	-1.698885319	0.001779566	Under expressed
XM_015094369.2	-1.697429112	0.084883641	Under expressed
XM_004010718.4	-1.697392618	0.142676804	Under expressed
XM_027965029.1	-1.696865017	0.137070471	Under expressed
XM_027958330.1	-1.695666365	0.121703652	Under expressed
XM_012106862.3	-1.695514093	0.026225941	Under expressed
XM_015094590.2	-1.694904733	0.057313324	Under expressed
XM_004006569.4	-1.693874839	0.031014591	Under expressed
XM_015102552.2	-1.693697683	0.022303457	Under expressed
XM_027977797.1	-1.693535693	0.083679115	Under expressed
XM_027979366.1	-1.692358308	0.060846925	Under expressed
XM_004011964.4	-1.69135136	0.047113604	Under expressed
XM_027974291.1	-1.687515765	0.036235275	Under expressed
XM_027968109.1	-1.68559795	0.047690331	Under expressed
XM_004008091.3	-1.682911016	0.079836256	Under expressed
XM_015101260.2	-1.682820218	0.008845687	Under expressed
XM_027970115.1	-1.682322332	0.109135099	Under expressed
XM_004003234.4	-1.68065614	0.019823259	Under expressed
XM_027965588.1	-1.680582912	0.072140886	Under expressed
XM_015091851.2	-1.680504582	0.02170058	Under expressed
XM_004016078.4	-1.680472952	0.060418906	Under expressed
XM_004003880.4	-1.679315947	0.143864565	Under expressed
XM_012132979.2	-1.678877347	0.008961388	Under expressed
XR_001024035.2	-1.678227094	0.001969923	Under expressed
XM_004004013.4	-1.677523705	0.008845687	Under expressed
XM_004023488.4	-1.675562838	0.073745649	Under expressed
XM_027976001.1	-1.674005858	0.127703235	Under expressed
XM_027960504.1	-1.672126819	0.00372592	Under expressed
XM_004012192.4	-1.671356828	0.011704526	Under expressed
XM_004021779.4	-1.671312641	0.021639394	Under expressed
XM_004004233.4	-1.670044351	0.12392445	Under expressed
XM_012182731.2	-1.665151688	0.005463185	Under expressed
XM_004013874.3	-1.663326819	0.016468414	Under expressed
XM_004010659.4	-1.663023904	0.073508015	Under expressed
XM_027975331.1	-1.662331845	0.041234351	Under expressed
XM_004018005.4	-1.661876625	0.007167606	Under expressed
XM_015098136.2	-1.661571664	0.128337423	Under expressed
XM_004021354.4	-1.661510705	0.00915629	Under expressed
XM_027976878.1	-1.661205494	0.095211005	Under expressed
XM_027973176.1	-1.661084899	0.084223096	Under expressed
XM_004011705.3	-1.660356526	0.082719313	Under expressed
XM_027960750.1	-1.659787931	0.057416218	Under expressed
XM_027974675.1	-1.658034629	0.010586881	Under expressed

XM_027972626.1	-1.657450417	0.145341556	Under expressed
XM_027957342.1	-1.656189085	0.005199432	Under expressed
XM_027979513.1	-1.654993855	0.054640495	Under expressed
XM_027972094.1	-1.653827675	0.017914835	Under expressed
XM_004008893.4	-1.65300556	0.041472179	Under expressed
XM_027957043.1	-1.652739022	0.003057442	Under expressed
NM_001009432.1	-1.65232214	0.094030309	Under expressed
XM_004003199.4	-1.651641984	0.033453964	Under expressed
XM_027966382.1	-1.65125614	0.053640158	Under expressed
XM_027956753.1	-1.650307608	0.081013101	Under expressed
XM_012098298.2	-1.649726901	0.044961253	Under expressed
XM_027967676.1	-1.649026468	0.040041218	Under expressed
XM_004011383.4	-1.648905047	0.079801577	Under expressed
XM_027966782.1	-1.647930352	5.14E-06	Under expressed
XM_004023649.3	-1.646135766	0.080833581	Under expressed
XM_004022178.4	-1.643811377	0.000254043	Under expressed
XM_004011156.3	-1.643743812	0.054989114	Under expressed
XM_004004691.4	-1.642799035	0.060021919	Under expressed
XM_027973699.1	-1.641755853	0.090386945	Under expressed
XM_004006797.4	-1.641286427	0.017741268	Under expressed
XM_015093680.2	-1.64117598	0.055685528	Under expressed
XM_027961116.1	-1.641167604	0.015377955	Under expressed
XR_003587472.1	-1.641018093	0.128485516	Under expressed
XM_027980145.1	-1.640873755	0.008617931	Under expressed
XM_004008689.4	-1.640817604	0.004976659	Under expressed
XM_027966778.1	-1.639855488	0.035935522	Under expressed
XM_004010676.4	-1.639780962	0.054657193	Under expressed
XM_004005121.4	-1.638926294	0.013509968	Under expressed
XM_015091600.2	-1.63871927	0.100946922	Under expressed
XM_027972354.1	-1.637259493	0.133584439	Under expressed
XM_004009030.4	-1.635761085	0.024172812	Under expressed
XM_004011208.3	-1.634073093	0.026004483	Under expressed
XM_027977190.1	-1.632033011	0.005940537	Under expressed
XM_004009859.4	-1.631923297	0.111091235	Under expressed
XM_004015332.4	-1.630502434	0.097178042	Under expressed
XM_004007714.3	-1.630111737	0.076846861	Under expressed
XM_004009111.4	-1.629601937	0.001699714	Under expressed
XM_012176906.2	-1.629042589	0.14598292	Under expressed
XM_004019613.4	-1.626265707	0.025346732	Under expressed
XM_027964258.1	-1.625729541	0.142567156	Under expressed
XM_027961291.1	-1.624494042	0.126992313	Under expressed
XM_015098720.2	-1.624449975	0.10561358	Under expressed
XM_004012679.4	-1.623619848	0.00295007	Under expressed
XM_004011722.4	-1.623084085	0.003285759	Under expressed

XM_012101307.2	-1.623071158	0.054989114	Under expressed
XM_012188134.3	-1.622331522	0.063135137	Under expressed
XM_027966696.1	-1.622011401	0.081423408	Under expressed
XM_004011277.4	-1.621656381	0.070637281	Under expressed
XM_004011408.4	-1.621313346	0.003362799	Under expressed
XM_004003309.4	-1.621299903	0.005416515	Under expressed
XM_027974368.1	-1.620475634	0.033309104	Under expressed
XM_027960482.1	-1.620443404	0.087691539	Under expressed
XM_027978863.1	-1.620182282	0.018894982	Under expressed
XM_004004373.4	-1.617989431	0.057852004	Under expressed
XM_027961866.1	-1.616871922	0.029126185	Under expressed
XM_004002262.4	-1.616794247	0.129683168	Under expressed
XM_027972544.1	-1.61575109	0.027856674	Under expressed
XM_027970935.1	-1.615447223	0.032525901	Under expressed
XM_012096987.3	-1.614795736	0.142975133	Under expressed
XM_015100425.2	-1.614151547	0.11185564	Under expressed
NM_001126367.2	-1.611039463	0.076823792	Under expressed
XM_027956234.1	-1.610861838	0.012635303	Under expressed
XM_004003134.4	-1.609946938	0.004272022	Under expressed
XM_004012987.4	-1.608616593	0.007266631	Under expressed
XR_001021684.3	-1.608088119	0.100946922	Under expressed
XM_027960746.1	-1.606478572	0.033810478	Under expressed
XM_004017067.3	-1.605530195	0.04167472	Under expressed
XM_004002076.4	-1.604147403	0.122004654	Under expressed
XM_015103489.2	-1.60392774	0.131403489	Under expressed
XM_027970506.1	-1.603553101	0.015383322	Under expressed
NM_001161865.1	-1.602253741	0.116339591	Under expressed
XM_027967849.1	-1.602085219	0.130933705	Under expressed
XM_027972423.1	-1.599994406	0.115627782	Under expressed
XM_027967211.1	-1.59994109	0.085845743	Under expressed
XM_027965013.1	-1.597920586	0.061913096	Under expressed
XM_004004475.4	-1.597550702	0.020033532	Under expressed
XM_015093478.2	-1.595714715	0.080979427	Under expressed
XM_015097289.2	-1.595233357	0.083974931	Under expressed
XM_027965048.1	-1.593858579	0.070494162	Under expressed
XM_027977495.1	-1.593436946	0.006812258	Under expressed
XM_004022232.4	-1.592775602	0.04177423	Under expressed
XM_027972632.1	-1.591097812	0.106335989	Under expressed
XM_027975327.1	-1.590783862	0.034005553	Under expressed
XM_004015015.3	-1.590027599	0.114196429	Under expressed
XM_004009739.4	-1.58819295	0.02924114	Under expressed
XM_012098138.3	-1.585328844	0.016534079	Under expressed
XM_004021529.3	-1.584977758	0.073189527	Under expressed
XM_012173651.2	-1.583519265	0.021548262	Under expressed

XM_027972281.1	-1.582944463	0.048171702	Under expressed
XR_001434000.2	-1.582736181	0.098453336	Under expressed
XM_027956296.1	-1.582302062	0.112303956	Under expressed
NM_001161889.1	-1.582053835	0.073591388	Under expressed
XM_004015981.4	-1.581441377	0.082416278	Under expressed
XM_004002393.4	-1.581217697	0.11401293	Under expressed
XM_004015998.3	-1.581167037	0.033424884	Under expressed
XM_027972390.1	-1.580663406	0.060235619	Under expressed
XM_027967675.1	-1.577222617	0.016463744	Under expressed
XR_003588777.1	-1.574872633	0.02545668	Under expressed
XM_004011404.4	-1.574684761	0.095415763	Under expressed
XM_027975112.1	-1.574034753	0.137339498	Under expressed
XM_004004446.4	-1.572123796	0.006184162	Under expressed
XM_012186934.3	-1.57038211	0.022689947	Under expressed
XM_015097524.2	-1.569050528	0.010789577	Under expressed
XM_004018425.4	-1.569031003	0.044142776	Under expressed
XM_012168489.3	-1.567249144	0.113919174	Under expressed
XM_012185134.3	-1.567089815	0.047634574	Under expressed
XM_012113177.2	-1.566818155	0.018634289	Under expressed
XM_012173925.3	-1.564997631	0.010915963	Under expressed
XM_004023410.4	-1.563673445	0.057460464	Under expressed
XM_004020762.4	-1.562679788	0.005875034	Under expressed
XM_027967251.1	-1.562500203	0.078496209	Under expressed
XM_027978923.1	-1.562267496	0.001285659	Under expressed
XM_012178284.3	-1.561804989	0.127369752	Under expressed
XM_012142353.3	-1.559819626	0.109608148	Under expressed
XM_004010600.4	-1.559754381	0.04085217	Under expressed
XM_027975285.1	-1.556819842	0.05037297	Under expressed
XM_004011327.3	-1.556655896	0.1039027	Under expressed
XM_004020033.3	-1.556429478	0.02686218	Under expressed
XM_004009046.4	-1.556000986	6.59E-05	Under expressed
XM_004014221.4	-1.55599078	0.07148779	Under expressed
NM_001145185.1	-1.555291631	0.076233534	Under expressed
XR_003586204.1	-1.555158444	0.043432759	Under expressed
XM_027972522.1	-1.554143679	0.052078933	Under expressed
XM_027966436.1	-1.553193387	0.114194937	Under expressed
XM_004012788.4	-1.552246743	0.085729313	Under expressed
XM_004017318.4	-1.552065726	0.00130016	Under expressed
XM_027980301.1	-1.551635644	0.036804468	Under expressed
XM_004019102.4	-1.548433688	0.018061258	Under expressed
XM_004016384.4	-1.547192447	0.121459753	Under expressed
XM_004015002.3	-1.546894835	0.137339498	Under expressed
XM_004014030.4	-1.546783715	0.040905381	Under expressed
XM_004013005.3	-1.546652041	0.022091556	Under expressed

XM_027962330.1	-1.54650327	0.082341258	Under expressed
XM_004017986.4	-1.546184281	0.117761176	Under expressed
XM_027972221.1	-1.54510787	0.145118602	Under expressed
XM_004006668.3	-1.54470824	0.1238171	Under expressed
XM_027977137.1	-1.544103968	0.006830787	Under expressed
XM_027956275.1	-1.543211635	0.053799132	Under expressed
XM_027962625.1	-1.542816111	0.119726866	Under expressed
XM_027970360.1	-1.541937888	0.000312229	Under expressed
XM_012187270.3	-1.540921788	0.018733979	Under expressed
XM_012099704.3	-1.540706882	0.13308255	Under expressed
XM_004017068.4	-1.54066872	0.044867291	Under expressed
XM_004020488.4	-1.540373615	0.114734629	Under expressed
XM_015104411.2	-1.537684105	0.109519562	Under expressed
XM_027968514.1	-1.535059814	0.026424755	Under expressed
XM_015103892.2	-1.534969427	0.02870297	Under expressed
XM_027966317.1	-1.53459109	0.040301781	Under expressed
XM_027972998.1	-1.533951575	0.12804382	Under expressed
XM_004006265.4	-1.533319584	0.0897164	Under expressed
XM_004017924.4	-1.532706292	2.32E-08	Under expressed
XM_027967202.1	-1.530395138	0.023300113	Under expressed
XM_004011183.4	-1.528887452	0.002342774	Under expressed
XM_027971381.1	-1.527583808	0.001444665	Under expressed
XR_001435222.2	-1.526819857	0.057575546	Under expressed
XM_015104450.2	-1.525848788	0.093839059	Under expressed
XM_015099631.2	-1.524657851	0.001560355	Under expressed
XM_027968510.1	-1.523226973	0.083645344	Under expressed
XM_015095342.2	-1.522414162	0.130321725	Under expressed
XM_027973241.1	-1.522187947	0.004630032	Under expressed
XM_004008657.4	-1.521928741	0.015262597	Under expressed
XM_027966272.1	-1.521431419	0.085187659	Under expressed
XM_004018315.3	-1.521008511	0.000292081	Under expressed
XM_004013599.4	-1.520910017	0.121098375	Under expressed
XR_003586274.1	-1.519331489	0.037423609	Under expressed
XM_004021733.4	-1.518398535	0.001259681	Under expressed
XR_003590768.1	-1.517679547	0.143047857	Under expressed
XM_015093178.2	-1.517585704	0.015406898	Under expressed
XM_004021042.4	-1.517132329	3.05E-05	Under expressed
XM_027956330.1	-1.51315328	0.000401367	Under expressed
XM_015103060.2	-1.511232589	0.038337281	Under expressed
XM_004013032.4	-1.50945751	0.016581038	Under expressed
XM_004017817.4	-1.506521081	0.14014596	Under expressed
XM_012162511.3	-1.506405451	0.00520491	Under expressed
XM_004015132.4	-1.503012043	0.006004311	Under expressed
XM_027963724.1	-1.501824394	0.090365753	Under expressed

XM_027977634.1	-1.501362416	0.003470239	Under expressed
XM_027979167.1	-1.501019841	0.021229749	Under expressed
XM_027966890.1	-1.500285053	0.075270666	Under expressed
XM_015098075.2	-1.498615145	0.065918033	Under expressed
XM_004010441.4	-1.497988612	0.133956634	Under expressed
XM_004013623.4	-1.497665005	0.009872716	Under expressed
XM_012120651.3	-1.496429728	0.004225515	Under expressed
XM_012105276.3	-1.495794244	0.139457359	Under expressed
NM_001163056.1	-1.493499764	0.010607269	Under expressed
XM_027972502.1	-1.490229006	0.124154399	Under expressed
XM_012133890.3	-1.489096351	0.142690967	Under expressed
XM_015099365.2	-1.486569684	0.094066078	Under expressed
XM_012190257.3	-1.486263202	0.000276847	Under expressed
XM_027971185.1	-1.486062841	0.109135099	Under expressed
XR_003586217.1	-1.485836195	0.097906682	Under expressed
XM_004002956.4	-1.483937992	0.029126185	Under expressed
XM_004022004.4	-1.483260451	0.009334221	Under expressed
XM_004008675.4	-1.482273856	0.012238846	Under expressed
XM_027973601.1	-1.480727074	0.101205531	Under expressed
XM_027966936.1	-1.480455787	0.008040319	Under expressed
XM_027971135.1	-1.479160307	0.013657581	Under expressed
XM_015095184.2	-1.478989124	0.003450801	Under expressed
XM_004002302.3	-1.478699488	0.021124401	Under expressed
XM_027959332.1	-1.478446775	0.037391752	Under expressed
XM_012137139.3	-1.478329266	0.000642333	Under expressed
XM_004002217.4	-1.477943907	0.111908316	Under expressed
XM_004011859.4	-1.477643494	0.015923826	Under expressed
XM_004016902.4	-1.477540099	0.120122994	Under expressed
XM_012175461.3	-1.476295601	0.069503187	Under expressed
XM_004017282.4	-1.475417687	0.07934326	Under expressed
XM_004021431.4	-1.474496263	0.088840036	Under expressed
XM_015103326.2	-1.473499697	0.014287905	Under expressed
XM_027978122.1	-1.470733008	0.01795119	Under expressed
XM_027956248.1	-1.469374434	0.11560351	Under expressed
XM_027958081.1	-1.468844832	0.113628781	Under expressed
XM_027978868.1	-1.468540446	0.005581919	Under expressed
XM_004013673.3	-1.468372497	0.073388282	Under expressed
XM_004011202.4	-1.467061417	0.01060164	Under expressed
XM_004011797.4	-1.466113762	0.000936587	Under expressed
XM_027961195.1	-1.466065945	0.006191794	Under expressed
XM_004017458.4	-1.465033095	0.035516273	Under expressed
XM_004017069.4	-1.463808883	0.07087811	Under expressed
XM_004011714.4	-1.463228123	0.11898354	Under expressed
XM_012116190.2	-1.461188808	0.064251997	Under expressed

XM_027960683.1	-1.460862536	0.011386162	Under expressed
XM_027978324.1	-1.459979863	0.141648127	Under expressed
XM_004011238.4	-1.459147699	0.063445055	Under expressed
XM_004011729.3	-1.459021769	0.05098375	Under expressed
XM_027978743.1	-1.455744449	0.010989268	Under expressed
XM_012107546.3	-1.455383567	0.03659084	Under expressed
XM_015092739.2	-1.455355812	0.099922906	Under expressed
XM_004012158.4	-1.453824384	0.091510024	Under expressed
XM_027959166.1	-1.453540184	0.061886192	Under expressed
XM_027973600.1	-1.453341599	0.002496539	Under expressed
XM_027974232.1	-1.452854288	0.022386677	Under expressed
XM_027966660.1	-1.452442545	2.39E-08	Under expressed
XM_004004225.3	-1.451172388	0.021421875	Under expressed
XM_027968745.1	-1.449258465	1.92E-07	Under expressed
XM_027967325.1	-1.449079577	0.078076941	Under expressed
XM_027979702.1	-1.448311567	0.006025018	Under expressed
XM_012097517.3	-1.446353321	0.092596225	Under expressed
XM_027972368.1	-1.446337888	0.128230622	Under expressed
XM_004006798.4	-1.441119994	0.002499074	Under expressed
XM_027966920.1	-1.439775791	0.000510182	Under expressed
XM_012180829.3	-1.437780742	0.007821905	Under expressed
XM_027961497.1	-1.437589181	0.132347662	Under expressed
XM_004021421.4	-1.43749268	0.057670162	Under expressed
XM_004004870.4	-1.437434419	0.026813214	Under expressed
XM_027975952.1	-1.436863026	0.139216841	Under expressed
XM_027959869.1	-1.43676156	0.01795119	Under expressed
XM_027958083.1	-1.435289111	0.147422528	Under expressed
XM_004007863.4	-1.430557511	0.086104836	Under expressed
XM_004006039.4	-1.427406737	0.054785296	Under expressed
XM_004009829.3	-1.426314733	0.125147887	Under expressed
XM_015091873.2	-1.42609962	0.129822452	Under expressed
XM_004005715.4	-1.425446798	0.018061599	Under expressed
XM_027961779.1	-1.425055708	0.024690172	Under expressed
XM_027976837.1	-1.424268858	0.043073883	Under expressed
XR_003589709.1	-1.423196845	0.050943756	Under expressed
XR_001023239.3	-1.423032345	0.000420255	Under expressed
XM_027977798.1	-1.420140273	0.104618029	Under expressed
XM_012183173.3	-1.42012706	0.061847952	Under expressed
XM_012181884.3	-1.419655539	0.008805434	Under expressed
XM_027975969.1	-1.419525957	0.061431822	Under expressed
XM_004013038.4	-1.41888796	0.098453336	Under expressed
XM_004008955.3	-1.417988386	0.00054657	Under expressed
XM_004017365.4	-1.41793407	0.06125059	Under expressed
XM_004017438.4	-1.417143494	0.149734613	Under expressed

XM_004016913.4	-1.416483745	0.141322302	Under expressed
XM_027976496.1	-1.4162736	0.057737215	Under expressed
XM_027971721.1	-1.415694898	0.002894228	Under expressed
NM_001009765.1	-1.414678264	0.053033863	Under expressed
XM_027958120.1	-1.413636501	0.11401293	Under expressed
XM_012174662.3	-1.410814728	0.085541994	Under expressed
XM_027968566.1	-1.410045474	0.058934401	Under expressed
XM_015095730.2	-1.407915723	0.149437976	Under expressed
XR_173470.3	-1.407622653	0.041819988	Under expressed
XM_012185875.2	-1.407190357	0.005199432	Under expressed
XM_027979690.1	-1.406899616	0.142639137	Under expressed
XR_001435100.2	-1.405632726	0.00846433	Under expressed
XM_004013003.4	-1.404297284	0.136149077	Under expressed
XM_027958257.1	-1.404010419	0.002474541	Under expressed
XM_015094394.2	-1.403829133	0.055147636	Under expressed
NM_001285801.1	-1.403409415	0.13313411	Under expressed
XM_027958182.1	-1.403147406	0.089666629	Under expressed
XM_004005841.3	-1.402271729	0.128596432	Under expressed
XM_004021747.4	-1.400988096	0.121107073	Under expressed
XM_015092018.2	-1.400698036	0.114601025	Under expressed
XM_027976730.1	-1.400551894	0.023350651	Under expressed
XM_012100862.3	-1.400031378	0.140644714	Under expressed
XM_012181791.3	-1.398180336	0.040249175	Under expressed
XM_027965346.1	-1.397807052	0.12197309	Under expressed
XM_027965526.1	-1.39668777	0.12754839	Under expressed
XM_015102005.2	-1.395372862	1.25E-05	Under expressed
XM_015097802.2	-1.394589459	0.050134426	Under expressed
XM_004009922.4	-1.39429617	0.004299885	Under expressed
XM_004011265.4	-1.391812463	0.077752099	Under expressed
XM_027964587.1	-1.390596544	0.003532941	Under expressed
XM_027977194.1	-1.389837362	0.009043296	Under expressed
XM_004005797.4	-1.386610367	2.10E-06	Under expressed
XM_004012482.4	-1.386190264	0.123706657	Under expressed
XM_027958589.1	-1.385712405	0.004594698	Under expressed
XM_027974871.1	-1.385593596	0.001236453	Under expressed
XM_004013902.4	-1.383272427	0.081599705	Under expressed
XR_001023055.3	-1.381986093	0.109135099	Under expressed
XR_003589146.1	-1.381068197	0.04867172	Under expressed
XM_015095380.2	-1.381012398	0.061303481	Under expressed
XM_004018197.4	-1.380422587	0.017229345	Under expressed
XM_004004371.3	-1.379649379	0.040905381	Under expressed
XM_027976703.1	-1.378595775	0.091428569	Under expressed
XM_027977301.1	-1.378140826	0.10065828	Under expressed
XM_012097890.2	-1.377837284	0.052931225	Under expressed

XM_004008108.4	-1.377049421	0.068050639	Under expressed
XM_027972450.1	-1.376985472	9.23E-05	Under expressed
XM_015104150.2	-1.376947963	0.013938911	Under expressed
NM_001306123.1	-1.376621119	0.005249634	Under expressed
XM_004003115.4	-1.375906703	0.013372331	Under expressed
XM_012097639.3	-1.375517524	0.142676804	Under expressed
XM_027967131.1	-1.373862697	0.094634355	Under expressed
XM_015094378.2	-1.373258978	0.01120223	Under expressed
XM_012179883.3	-1.372356373	0.006653519	Under expressed
XM_004006425.4	-1.372201606	0.001358998	Under expressed
XM_012097393.3	-1.371555151	0.055278649	Under expressed
XM_012096203.3	-1.371204116	0.08305718	Under expressed
XM_027967239.1	-1.369436402	0.006288141	Under expressed
XM_015097121.2	-1.368813326	0.020010326	Under expressed
XM_004007725.3	-1.367707779	0.069059029	Under expressed
XM_004018518.4	-1.367121482	0.098453336	Under expressed
XM_015096799.2	-1.366977929	0.146691955	Under expressed
XM_004010769.4	-1.366555606	0.003883782	Under expressed
XM_027972267.1	-1.362920798	0.118566323	Under expressed
XM_012098936.3	-1.362771157	0.020355676	Under expressed
XM_004011248.4	-1.361979127	0.094444099	Under expressed
XM_004020510.4	-1.361236934	0.109485006	Under expressed
XM_004001991.4	-1.360669972	0.012245425	Under expressed
XM_004002792.4	-1.360369145	0.083066409	Under expressed
XM_027961901.1	-1.360316847	0.053434304	Under expressed
XM_027957825.1	-1.358325684	0.135732062	Under expressed
XM_004009022.4	-1.358123439	0.048024432	Under expressed
XM_004008500.4	-1.357905136	0.00857931	Under expressed
XM_012139743.3	-1.357352923	0.00746071	Under expressed
XM_027972220.1	-1.35719204	0.09497726	Under expressed
XM_012162838.3	-1.356814484	0.101934011	Under expressed
XM_027970549.1	-1.356437889	0.027450496	Under expressed
XM_027965176.1	-1.355330078	0.062748005	Under expressed
XM_004009048.4	-1.355226685	0.015262597	Under expressed
XM_004004727.4	-1.354975424	0.035795462	Under expressed
XM_012102124.3	-1.354740408	0.063153816	Under expressed
XM_004005777.4	-1.354462707	0.116327325	Under expressed
XM_027961696.1	-1.354406722	0.131249752	Under expressed
XM_015099595.2	-1.354086926	0.061571085	Under expressed
XM_015102542.2	-1.353237797	0.040180482	Under expressed
XM_027964979.1	-1.352771479	0.055213011	Under expressed
XM_004014340.4	-1.352023014	0.058765294	Under expressed
XM_004006708.4	-1.350514916	0.019461581	Under expressed
XM_027977454.1	-1.350430326	0.016610802	Under expressed

XM_004009652.4	-1.349721697	0.055890225	Under expressed
XM_004002669.4	-1.34918925	0.029106149	Under expressed
XM_004012221.4	-1.348029749	0.134743533	Under expressed
XM_027961155.1	-1.344339067	0.081599705	Under expressed
XM_027977307.1	-1.34401015	0.070494162	Under expressed
XM_027977849.1	-1.342916552	0.077029893	Under expressed
XM_004005829.4	-1.342810108	0.025140065	Under expressed
XM_004012799.4	-1.341152122	0.008363405	Under expressed
NM_001129741.1	-1.34030608	0.078272281	Under expressed
XM_004006835.4	-1.340169595	0.084518502	Under expressed
XM_004012476.4	-1.339927812	0.074207643	Under expressed
XM_004017231.4	-1.33808787	0.010579015	Under expressed
XM_027978680.1	-1.337917785	1.19E-05	Under expressed
XM_004006488.4	-1.337266563	0.00438745	Under expressed
XR_003586280.1	-1.334874023	0.134188408	Under expressed
XM_004017405.4	-1.333010055	0.130933705	Under expressed
XM_004005116.3	-1.332938846	0.148489889	Under expressed
XM_027962603.1	-1.331442543	0.045256103	Under expressed
XM_004004043.3	-1.331059447	0.094310525	Under expressed
XM_027961577.1	-1.330624619	0.090253103	Under expressed
XM_015096632.2	-1.330573965	0.024714789	Under expressed
XM_015093177.2	-1.329864776	0.10197323	Under expressed
XM_004009006.4	-1.329733172	0.043085938	Under expressed
XM_004011660.4	-1.32965191	0.050342417	Under expressed
XM_004012406.4	-1.328819564	0.001525587	Under expressed
XM_027962450.1	-1.326746293	0.133956634	Under expressed
XM_027960932.1	-1.326337707	0.035594283	Under expressed
XM_012178564.3	-1.32536685	0.005533585	Under expressed
XM_004002826.4	-1.324235616	0.006204916	Under expressed
XM_027975343.1	-1.322788155	1.07E-05	Under expressed
XM_027972014.1	-1.322442307	0.001221522	Under expressed
XM_027976207.1	-1.321669697	0.012961531	Under expressed
XM_004003420.3	-1.313330994	0.054864137	Under expressed
XM_027961844.1	-1.312785608	0.025010063	Under expressed
XM_027975255.1	-1.312608147	0.127903557	Under expressed
XM_027959008.1	-1.312391268	0.055900731	Under expressed
XM_004006014.4	-1.311493691	0.017763518	Under expressed
XM_027964182.1	-1.310440017	0.026332895	Under expressed
XM_004006104.4	-1.310423057	0.0897164	Under expressed
XM_004008793.4	-1.307813355	0.133584439	Under expressed
XM_004006701.4	-1.307168312	0.002053308	Under expressed
XM_012098800.3	-1.307161624	0.122944123	Under expressed
XM_027974463.1	-1.3068728	0.048660169	Under expressed
XM_004006982.4	-1.306838535	0.117290662	Under expressed

XM_004006767.3	-1.306118245	0.12732052	Under expressed
XM_015098467.2	-1.30596999	0.001851564	Under expressed
XM_015099893.2	-1.305332957	0.093582539	Under expressed
XM_012186164.3	-1.305252298	2.87E-05	Under expressed
XM_004012734.3	-1.304942843	0.097574786	Under expressed
XM_012185299.3	-1.303653992	0.001205515	Under expressed
XM_004003083.4	-1.303483348	0.148077401	Under expressed
XM_004012425.4	-1.303423682	0.111567921	Under expressed
XM_012175114.3	-1.302157713	0.133008254	Under expressed
XM_004023151.4	-1.302002246	0.022349694	Under expressed
XM_012179809.3	-1.300732486	0.069076556	Under expressed
XM_004017531.4	-1.29997366	0.139532612	Under expressed
XM_004011844.3	-1.299862751	0.001559245	Under expressed
XM_027969060.1	-1.299294541	0.030579045	Under expressed
XM_027975119.1	-1.299254041	0.117579365	Under expressed
XM_004011168.4	-1.297571276	0.115180028	Under expressed
XM_004002085.4	-1.296890718	0.00527025	Under expressed
XM_015095971.2	-1.295438389	0.123894856	Under expressed
XM_004003027.4	-1.295027145	0.08091314	Under expressed
XM_027971326.1	-1.295006995	0.061431822	Under expressed
XM_027964117.1	-1.294270988	0.080820671	Under expressed
XM_027980025.1	-1.294082849	0.075660032	Under expressed
XM_012106241.3	-1.29361731	0.038793587	Under expressed
XM_027976294.1	-1.293164941	0.000335014	Under expressed
XM_027972926.1	-1.292983174	0.061886192	Under expressed
XM_015103787.2	-1.292199201	0.000780441	Under expressed
XM_027978917.1	-1.29119207	0.055777282	Under expressed
XM_027973696.1	-1.290204673	0.047872437	Under expressed
XM_015091597.2	-1.289445787	0.037413702	Under expressed
XM_027976983.1	-1.28849053	0.050209353	Under expressed
XM_027956731.1	-1.287850038	7.65E-07	Under expressed
XM_015096790.2	-1.286586792	5.89E-06	Under expressed
XM_004002381.4	-1.285652541	0.10711526	Under expressed
XM_004020157.4	-1.285062389	0.000199263	Under expressed
XM_004020428.4	-1.2839376	0.049857534	Under expressed
XM_004018847.3	-1.283708453	0.125601875	Under expressed
XR_173257.4	-1.282229646	0.081046239	Under expressed
XM_004002942.4	-1.280512619	8.35E-08	Under expressed
XM_027980246.1	-1.280342161	0.107687738	Under expressed
XM_027975186.1	-1.280308613	0.040091516	Under expressed
XM_004020255.3	-1.280134388	0.037325588	Under expressed
XR_001023051.2	-1.27999903	0.005728053	Under expressed
XM_027964046.1	-1.279827681	0.114830617	Under expressed
XM_012177637.2	-1.279683257	0.086422197	Under expressed

XM_004008227.4	-1.278721925	0.015738733	Under expressed
XM_004020199.4	-1.277928123	0.133471132	Under expressed
XM_004002063.3	-1.277849082	0.099010274	Under expressed
XM_004013176.4	-1.277185027	0.010662357	Under expressed
XM_015094386.2	-1.275667691	0.011159308	Under expressed
XM_004006512.4	-1.275599556	0.008792602	Under expressed
XM_027970992.1	-1.275578493	0.011322098	Under expressed
XM_004010517.4	-1.274150869	0.017793199	Under expressed
XM_015096436.2	-1.271643927	0.091811836	Under expressed
XR_003591055.1	-1.271066382	0.077638884	Under expressed
XM_012106423.3	-1.270258738	0.098471359	Under expressed
XM_004002962.4	-1.270088781	0.022303457	Under expressed
XM_012099396.3	-1.268626314	0.000575088	Under expressed
XM_004002359.4	-1.268263249	0.083747551	Under expressed
XM_004009795.4	-1.265618116	0.0271568	Under expressed
XM_027970255.1	-1.26522502	0.026868418	Under expressed
XM_027974270.1	-1.264151703	0.115180028	Under expressed
XM_027966886.1	-1.261612595	0.037297127	Under expressed
XM_004017222.3	-1.259721285	0.075075778	Under expressed
XM_015102015.2	-1.254979356	0.027637143	Under expressed
XM_027962360.1	-1.254885823	0.044901775	Under expressed
XM_027973879.1	-1.25261631	0.036086358	Under expressed
XM_027979603.1	-1.252447758	0.068576147	Under expressed
XM_027971892.1	-1.252085435	0.010237078	Under expressed
XR_003589400.1	-1.250579628	0.144032633	Under expressed
XM_004016582.4	-1.248118305	0.000409787	Under expressed
XM_027966437.1	-1.248032924	0.013657581	Under expressed
XM_015103563.2	-1.247805857	0.129315715	Under expressed
XM_027958963.1	-1.247777723	0.146066494	Under expressed
XM_015098684.2	-1.246775068	0.122098693	Under expressed
XM_004004770.4	-1.24634476	0.077117892	Under expressed
XM_004009714.4	-1.246006177	0.009636818	Under expressed
XM_004016917.4	-1.245581371	0.108179611	Under expressed
NM_001009761.1	-1.244482356	6.12E-06	Under expressed
XM_012102945.3	-1.243145555	0.076823792	Under expressed
XM_004016888.3	-1.243015691	4.00E-05	Under expressed
XM_012185167.3	-1.24293113	0.034780821	Under expressed
XR_003586699.1	-1.242383592	0.02431223	Under expressed
XM_004014257.4	-1.241013133	0.099487685	Under expressed
XM_004016034.4	-1.240397439	0.030661225	Under expressed
XM_004016952.4	-1.240247815	0.117748156	Under expressed
XM_004011067.4	-1.239555098	0.001055116	Under expressed
XM_004002540.4	-1.238607627	0.124072705	Under expressed
XR_003588622.1	-1.238422156	0.064891664	Under expressed

XM_004010748.4	-1.237996102	0.037073994	Under expressed
XM_012178899.3	-1.237114673	0.022102665	Under expressed
XM_004019444.4	-1.237023724	0.033198502	Under expressed
XR_003586287.1	-1.235923972	0.040249175	Under expressed
XM_012175577.2	-1.234448856	0.055980039	Under expressed
XM_027960198.1	-1.234314575	0.044299126	Under expressed
XM_004021749.4	-1.233995632	0.05329855	Under expressed
XM_004006009.3	-1.233975666	0.024378514	Under expressed
XM_004020787.4	-1.231940261	0.030212212	Under expressed
XM_004014553.4	-1.231556843	0.09363129	Under expressed
XM_004004357.3	-1.231367478	0.008154444	Under expressed
XM_004002414.3	-1.230175311	0.123713244	Under expressed
XM_012186433.3	-1.228806821	0.037222165	Under expressed
XM_027960634.1	-1.228520378	0.001221596	Under expressed
XM_027971654.1	-1.227892752	0.017715929	Under expressed
XM_012097773.3	-1.227053516	0.114194937	Under expressed
XM_012122011.3	-1.226112089	0.059926809	Under expressed
XM_027974337.1	-1.225999618	0.03049649	Under expressed
XM_004003013.4	-1.225742905	0.140691344	Under expressed
XM_027978733.1	-1.225476024	0.090040828	Under expressed
XM_004015478.4	-1.225270578	0.081046239	Under expressed
XM_027968227.1	-1.223694235	0.011979828	Under expressed
XM_027968908.1	-1.223212591	0.048195485	Under expressed
XM_012099990.3	-1.222166277	0.017516677	Under expressed
XM_012105183.3	-1.220586136	0.109135099	Under expressed
XM_004001948.3	-1.219650767	0.135482323	Under expressed
XM_012184609.3	-1.218919315	0.114734629	Under expressed
XM_004004697.4	-1.218804504	0.011605044	Under expressed
XM_027956643.1	-1.216714309	0.08471501	Under expressed
XM_027969057.1	-1.214849696	1.38E-07	Under expressed
XM_027971787.1	-1.213523883	0.012213552	Under expressed
XM_027962723.1	-1.210613521	0.116339591	Under expressed
XM_004011170.3	-1.206873263	0.149516883	Under expressed
XM_015095742.2	-1.206284124	0.016091368	Under expressed
XM_027971956.1	-1.205398934	0.114734629	Under expressed
XM_004015004.4	-1.204328293	0.004243744	Under expressed
XM_012186252.3	-1.204240122	0.087572253	Under expressed
XM_012099374.3	-1.20047067	0.077188914	Under expressed
XM_027977894.1	-1.200437035	0.071666557	Under expressed
XM_004005076.4	-1.196631822	0.075382461	Under expressed
XM_004010602.4	-1.196608844	0.005587828	Under expressed
XM_027957375.1	-1.196573907	0.13799039	Under expressed
XM_027962513.1	-1.195170792	0.00681404	Under expressed
XM_004010512.4	-1.19469207	0.024193999	Under expressed

XM_027967749.1	-1.190692634	0.032525901	Under expressed
NM_001009762.1	-1.1905331	0.088837651	Under expressed
XM_027964810.1	-1.190346671	0.063723527	Under expressed
XM_027956509.1	-1.186589229	0.116339591	Under expressed
XM_004005581.4	-1.186439144	0.020335664	Under expressed
XM_027956423.1	-1.182396743	0.103299359	Under expressed
XM_027966511.1	-1.181162189	0.128334757	Under expressed
XM_004006737.4	-1.178135441	0.033115292	Under expressed
NM_001348418.1	-1.177923727	0.006940337	Under expressed
XR_003591330.1	-1.176495429	0.103299359	Under expressed
XM_004019116.4	-1.176109378	0.097203793	Under expressed
XM_004020785.3	-1.175565408	0.012064496	Under expressed
XM_027977385.1	-1.173791128	0.000496173	Under expressed
XM_027967139.1	-1.173694053	0.027418944	Under expressed
XM_004003376.4	-1.171948935	0.044694607	Under expressed
XR_173202.4	-1.171003044	0.14474187	Under expressed
XM_027968858.1	-1.167967081	0.004243744	Under expressed
XR_003588218.1	-1.167492075	0.093913245	Under expressed
XM_027962606.1	-1.167461138	0.051378708	Under expressed
XM_004017911.4	-1.16703165	0.000331114	Under expressed
XM_004020062.4	-1.16675067	0.062740582	Under expressed
NM_001009234.1	-1.166478104	0.091353862	Under expressed
XM_004009920.3	-1.165443014	0.078734247	Under expressed
XM_012156222.3	-1.164673267	0.023894404	Under expressed
XM_004003300.4	-1.163896809	0.092859698	Under expressed
XM_027965566.1	-1.160975352	0.06162607	Under expressed
XM_004011662.3	-1.160672858	0.00266463	Under expressed
XM_012113497.3	-1.158890033	0.102647913	Under expressed
XM_004022279.4	-1.158362009	0.093862844	Under expressed
XM_004005671.3	-1.157802883	0.038433366	Under expressed
XM_004002431.4	-1.155802988	0.050774793	Under expressed
XM_004005014.4	-1.155159835	0.028801284	Under expressed
XM_015099542.2	-1.153136789	0.043089562	Under expressed
XM_004003221.4	-1.152767897	0.067316641	Under expressed
XM_004005698.4	-1.15195167	0.019705513	Under expressed
XM_012100619.3	-1.150722846	0.000209242	Under expressed
XM_015101910.2	-1.150333804	0.022722064	Under expressed
XM_012183714.3	-1.150156454	0.012910881	Under expressed
XM_004002714.3	-1.149501476	0.083679115	Under expressed
XM_015095297.2	-1.148330224	0.125571928	Under expressed
XM_027959693.1	-1.148119363	0.114276131	Under expressed
XM_004013369.4	-1.145507173	0.11382484	Under expressed
XM_015092295.2	-1.14544851	0.108417189	Under expressed
XM_004001995.4	-1.142591938	0.052236032	Under expressed

XM_027979164.1	-1.140773631	0.01795119	Under expressed
XM_012126676.3	-1.140449588	0.004848344	Under expressed
XM_004002260.4	-1.139987859	0.005882631	Under expressed
XM_027960952.1	-1.139510869	0.006164355	Under expressed
XR_003587162.1	-1.1393971	0.055900731	Under expressed
XM_004021456.4	-1.139067413	0.039974695	Under expressed
XM_027980116.1	-1.138952788	4.94E-05	Under expressed
XM_004008987.3	-1.138943288	0.144983001	Under expressed
XM_015099798.2	-1.138689678	0.139090988	Under expressed
XM_027956500.1	-1.138322811	0.012910881	Under expressed
XM_027956650.1	-1.138297939	9.18E-05	Under expressed
XM_027965717.1	-1.137956678	0.022102665	Under expressed
XM_012190289.3	-1.136208372	0.020511643	Under expressed
XM_004017910.3	-1.136105534	6.07E-09	Under expressed
XM_012148762.3	-1.135457597	0.110611857	Under expressed
XM_027968900.1	-1.135451112	0.033102655	Under expressed
XM_004002248.4	-1.135103013	0.025194359	Under expressed
XR_003587037.1	-1.134454523	0.0143192	Under expressed
XM_015092353.2	-1.132498633	1.25E-11	Under expressed
XM_004002382.3	-1.131599347	0.064507539	Under expressed
XM_027960457.1	-1.131586854	0.115642766	Under expressed
XR_001042197.2	-1.130765915	0.078081541	Under expressed
XM_004010368.4	-1.130457923	0.100241732	Under expressed
XM_004020433.4	-1.129500408	0.008768154	Under expressed
XM_027959114.1	-1.128398675	0.028236976	Under expressed
XM_004005910.4	-1.128148288	0.009713468	Under expressed
XM_004005912.4	-1.126863013	0.034465591	Under expressed
XM_004016124.4	-1.126503057	0.070664191	Under expressed
XM_015093251.2	-1.125958371	0.003013092	Under expressed
XR_003586278.1	-1.125613543	0.098301605	Under expressed
XM_027961544.1	-1.124872763	0.013093738	Under expressed
XM_015096234.2	-1.124753357	0.025890047	Under expressed
XM_015093602.2	-1.121411002	0.011082114	Under expressed
XM_004002270.3	-1.120282892	0.074207643	Under expressed
XM_027979637.1	-1.11621778	0.066753208	Under expressed
XM_027979801.1	-1.11595611	0.060408811	Under expressed
XM_012182734.3	-1.115459467	0.001797027	Under expressed
XM_027963624.1	-1.115198521	0.093128719	Under expressed
XM_012181995.2	-1.114322086	0.030956278	Under expressed
XM_004001795.4	-1.114215556	0.0055037	Under expressed
XM_027957304.1	-1.112763514	0.023300113	Under expressed
XM_027972953.1	-1.110385746	0.113125338	Under expressed
XM_004004598.3	-1.108563199	0.125641401	Under expressed
XM_004017947.4	-1.108381316	3.76E-06	Under expressed

NM_001161864.1	-1.107994747	0.076846861	Under expressed
NM_001146090.1	-1.107534751	0.097670933	Under expressed
XM_004017834.4	-1.107179801	0.049224045	Under expressed
XM_027967991.1	-1.104286888	0.135058947	Under expressed
XM_004012527.3	-1.103825118	0.010922841	Under expressed
XM_004004498.4	-1.103241119	0.000243028	Under expressed
XM_027973654.1	-1.102600994	0.084518502	Under expressed
XM_015098276.2	-1.100816152	0.003430047	Under expressed
NM_001009426.1	-1.099925013	0.142676804	Under expressed
XM_004011785.3	-1.099572836	0.084518502	Under expressed
XM_004002396.4	-1.099286361	0.139019656	Under expressed
XM_015099185.2	-1.099208841	0.123224974	Under expressed
XM_027971133.1	-1.097028927	0.074993762	Under expressed
XM_015096684.2	-1.096713273	0.003904494	Under expressed
XM_004006704.4	-1.096450226	0.121459753	Under expressed
XM_015095384.2	-1.095686344	0.006164355	Under expressed
XM_015099649.2	-1.095439161	0.004326465	Under expressed
XM_012116077.2	-1.095289499	0.092441509	Under expressed
XM_012105143.3	-1.095283275	0.031948203	Under expressed
XM_027970004.1	-1.094376659	0.084883641	Under expressed
XM_015102267.2	-1.092360665	0.011966257	Under expressed
XM_027971070.1	-1.092078516	0.0484073	Under expressed
XM_004002410.4	-1.092050193	0.002207834	Under expressed
XR_003591421.1	-1.091157052	0.083066409	Under expressed
XR_003591329.1	-1.088982294	0.099181007	Under expressed
NM_001009402.2	-1.08699029	0.000814822	Under expressed
XM_004002172.4	-1.086795105	0.022452826	Under expressed
XM_004019415.4	-1.086396413	0.020188871	Under expressed
XM_012094886.3	-1.085319488	0.030471718	Under expressed
XM_004006770.4	-1.084470815	0.139337763	Under expressed
XM_027956305.1	-1.083909342	0.03077649	Under expressed
XM_027980128.1	-1.083620985	0.002558817	Under expressed
XM_015096913.2	-1.083504451	0.007275229	Under expressed
XM_027966883.1	-1.082677886	0.051076808	Under expressed
XM_004019417.4	-1.081911089	0.072328652	Under expressed
XM_027968873.1	-1.081202834	0.069441481	Under expressed
XM_004003185.4	-1.078756488	0.053866104	Under expressed
XM_012100410.2	-1.078032656	0.128230622	Under expressed
XR_003589869.1	-1.077697677	0.008154444	Under expressed
XM_027974810.1	-1.077625195	0.006805076	Under expressed
XM_027973627.1	-1.075977677	0.04821245	Under expressed
XM_004013683.4	-1.075419413	0.013130177	Under expressed
XM_004002061.3	-1.075192791	0.059926809	Under expressed
XM_004019550.4	-1.075049516	0.073189527	Under expressed

XM_027962224.1	-1.074967423	0.113547427	Under expressed
XM_012173447.2	-1.073621368	0.103938512	Under expressed
XM_015099354.2	-1.073603942	0.115757473	Under expressed
XM_012104929.3	-1.072625024	0.119499116	Under expressed
XM_027978037.1	-1.071233053	0.133956634	Under expressed
XM_012097501.3	-1.071215748	0.148300609	Under expressed
XM_004011164.4	-1.069072915	0.014635662	Under expressed
XM_027970054.1	-1.069066626	0.035066574	Under expressed
XM_004010821.3	-1.067792274	0.033842741	Under expressed
XM_004012055.4	-1.066929588	0.113919174	Under expressed
XM_004005974.4	-1.06180992	0.066615017	Under expressed
XM_012189881.2	-1.058038704	0.059518391	Under expressed
XM_027978674.1	-1.057821774	0.138021514	Under expressed
XM_027962725.1	-1.057695573	0.147632137	Under expressed
XM_004010601.4	-1.056006443	0.035136979	Under expressed
XR_003590230.1	-1.055737997	0.055378271	Under expressed
XM_012184734.3	-1.052856852	0.012639426	Under expressed
XM_012099024.3	-1.050810956	5.45E-05	Under expressed
XM_015097083.2	-1.049296421	0.127642024	Under expressed
XM_004023448.4	-1.047925668	0.020033532	Under expressed
XM_012184994.2	-1.045032751	0.096889668	Under expressed
XM_004011624.4	-1.04434225	0.05053463	Under expressed
XM_027970252.1	-1.044232402	0.030324471	Under expressed
XM_004007950.3	-1.042810721	0.025342248	Under expressed
XM_012189330.3	-1.041653	0.078748788	Under expressed
XM_004021795.4	-1.040675577	0.128213235	Under expressed
XM_004016010.4	-1.039944716	0.116472266	Under expressed
XM_027956263.1	-1.038123312	0.107604656	Under expressed
XM_004012545.4	-1.036121152	0.107697796	Under expressed
XM_027974336.1	-1.03357379	0.089987196	Under expressed
XM_027973259.1	-1.032935977	0.050774793	Under expressed
XM_027956447.1	-1.029055617	0.050303212	Under expressed
XM_027974389.1	-1.026032191	0.087521082	Under expressed
XM_015103086.2	-1.024686801	0.104824887	Under expressed
XM_027970055.1	-1.023743393	0.05435183	Under expressed
XM_004013554.4	-1.022718077	0.036187875	Under expressed
XM_015100431.2	-1.021787978	0.064934657	Under expressed
XM_027962872.1	-1.019622124	0.145314929	Under expressed
XM_027966699.1	-1.019291333	0.036039011	Under expressed
XM_012094697.3	-1.018128467	0.057922548	Under expressed
XM_004012582.3	-1.017770826	0.007928898	Under expressed
XM_004019432.4	-1.01725792	0.006384888	Under expressed
XM_004005781.3	-1.016949974	0.086106863	Under expressed
XM_027960230.1	-1.01412253	0.066419921	Under expressed

XM_012180676.3	-1.013331982	0.116394995	Under expressed
XM_027976331.1	-1.010681025	0.00439289	Under expressed
XM_027971454.1	-1.008805113	0.126277833	Under expressed
XM_015098787.2	-1.00865079	0.050315067	Under expressed
XM_012101804.3	-1.00803371	0.101186784	Under expressed
XM_027972143.1	-1.007565524	0.139532612	Under expressed
XM_004009064.3	-1.006992937	0.118464172	Under expressed
XM_027978447.1	-1.006530034	0.106958846	Under expressed
XM_012100702.3	-1.00588144	0.08081515	Under expressed
XM_012106769.3	-1.005853739	0.051417105	Under expressed
XM_027978869.1	-1.005527979	0.090878837	Under expressed
XM_027969068.1	-1.004930675	0.07218715	Under expressed
XM_015102846.2	-1.004686024	0.139058712	Under expressed
XM_027969667.1	-1.004553911	0.027450496	Under expressed
XM_004002909.4	-1.000797992	0.114827141	Under expressed

Table S4. List of differentially expressed genes splitted per KEGG metabolic pathway.

Kegg-code	Pathway	Over-expressed	Under-expressed
**Carbohydrate metabolism**			
oas00520	Amino sugar and nucleotide sugar metabolism	HEXA;GMDS;NAGK;PMM1;NANS;NADH-cytochrome b5 reductase 3 isoform X3RENBP;MPI;NADH-cytochrome b5 reductase 1beta-hexosaminidase subunit beta-like isoform X1TSTA3;FUK;AMDHD2;GMPPA;GALK1;GALT	cytidine monophosphate-N-acetylneuraminic acid hydroxylase isoform X1;UAP1;GNE;PGM2;PGM3;GNPNAT1;UGP2;GFPT1;cytochrome b5 reductase 4;FPGT
oas00051	Fructose and mannose metabolism	GMDS;PMM1;ALDOC;TKFC;MPI;TPI1;KHK;TSTA3;FUK;SORD;GMPPA;ALDOA;PFKL	FPGT
oas00052	Galactose metabolism	G6PC3;GALK1;GALT;PFKL	B4GALT1;PGM2;UGP2
oas00010	Glycolysis and gluconeogenesis	ALDOC;TPI1;PARP6;aldehyde dehydrogenase family 3 member B1 isoform X1;ALDH3B1;G6PC3;AKR1A1;ALDOA;LDHA;PFKL	PGM2
oas00030	Pentose phosphate pathway	ALDOC;PGLS;GLYCTK;ALDOA;PFKL	deoxyribose-phosphate aldolase;RPIA;PGM2
oas00620	Pyruvate metabolism	FH;PARP6;LDHD;LDHA;MDH2	ACAT1;GRHPR
oas00640	Propanoate metabolism	BCKDHA;ECHS1;SUCLG1;LDHA	ACAT1;ALDH6A1;HIBCH
oas00020	TCA cycle	FH;SDHB;IDH3G;SUCLG1;MDH2	ACLY
**Energy metabolism**			
oas00190	Oxidative phosphorylation	NDUFA7;NDUFA3;LHPP;PPA2;NDUFA13;cytochrome c oxidase subunit 4 isoform 1, mitochondrial;ATP6V1B1;SDHB;cytochrome b-c1 complex subunit 1, mitochondrial;cytochrome c oxidase subunit 5B, mitochondrial;ATP5ME;NDUFB10;ATP6V1F;NDUFS8;TCIRG1;cytochrome c1, heme protein, mitochondrial;NDUFS7;ATP5F1D;NDUFB11;cytochrome b-c1 complex subunit 10;NDUFV1;NDUFB7;NDUFA10;ATP6V0A1;ATP5MC1	ATP6V1A;PPA1
oas00920	Sulfur metabolism	TST;MPST	IMPAD1
**Lipid metabolism**			
oas01040	Biosynthesis of unsaturated fatty acid	ACAA1	ELOVL5;ELOVL1;SCD
oas00062	Fatty acid elongation	ECHS1;MECR	ELOVL5;ELOVL6
oas00071	Fatty acid degradation	ACSL6;ACAA1;ECHS1;ECI1;ECI2;CPT1B	ACAT1;CPT2;ACSL5

oas00140	Steroid biosynthesis	CYP2D6;TOMT;HSD17B1;HSD17B8;SULT2B1	HSD17B7;HSD11B1
oas00561	Glycerolipid metabolism	TKFC;LPIN3;GLYCTK;AGPAT2;LIPC;GPAT4;AKR1A1;AGPAT1	DGKE;AGPAT1;LPL
oas00564	Glycerophospholipid metabolism	PLD4;LPIN3;PTDSS2;MBOAT7;TAZ;CHPT1;LPCAT1;PEMT;PGS1;PCYT2;AGPAT2;GPAT4;PLD3;CHKB;CDIPT;AGPAT1	SELENOI;CEPT1;ETNK1;GPCPD1;LPCAT2;PLA2G12A;DGKE;AGPAT1
oas00565	Ether lipid metabolism	PAFAH1B3;PLD4;CHPT1;LPCAT1;PAFAH1B2;PLD3	AGPS;SELENOI;CEPT1;LPCAT2;PLA2G12A;PLA2G7
oas00600	Sphingolipid metabolism	SMPD1;SPHK1;SMPD2;GBA;ARSA	SPHK2;SGPL1;UGCG;SGMS1
oas00590	Arachidonic metabolism	GPX1;PTGES2;ALOX12;carbonyl reductase [NADPH] 3;PRXL2B;arachidonate 15-lipoxygenase B	ALOX15;PLA2G12A;LTA4H;PTGS2
**Nucleotid metabolism**			
oas00230	Purine metabolism	AK1;IMPDH1;HDDC3;APRT;5-hydroxyisourate hydrolase-like isoform X2;NT5C3B;NME4;PARP6;AK8;uncharacterized protein LOC101117419 isoform X1;ITPA;GUK1;NUDT2;NUDT16	AMPD3;PDE4D;NT5C2;PGM2;AK3;ENPP4;PDE7A;ADK;RRM2B
oas00240	Pyrimidine metabolism	DHODH;NT5C3B;NME4;DUT;DTYMK;DCTPP1;NUDT2	CMPK1;DPYD;NT5C2;RRM2B
**Amino acid metabolism**			
oas00250	Alanine, aspartate and glutamate metabolism	ASL	L-amino-acid oxidase-like;DDO;GFPT1;GLS
oas00260	Glycine, serine and threonine metabolism	GLYCTK;SDSL;GAMT	GRHPR;serine racemase isoform X1;CTH
oas00270	Cysteine and methionine metabolism	DNMT3A;TST;MPST;SDSL;ENOPH1;LDHA;MDH2	L-amino-acid oxidase-like;MTAP;AHCYL1;AMD1;AHCYL2;GCLM;CTH;MAT2B
oas00280	Valine leucine and isoleucine metabolism	BCKDHA;ACAA1;ECHS1;HSD17B10	L-amino-acid oxidase-like;ACAT1;ALDH6A1;HIBCH;MCCC2;HMGCS1
oas00310	Lysine degradation	PLOD1;ECHS1;COLGALT1;DOT1L	ACAT1;SETDB1;histone-lysine N-methyltransferase KMT5B-like;NSD3;SETD2;CAMKMT;SUV39H2;ASH1L;EZH2;NSD1;PRDM2

oas00330	Arginine and proline metabolism	SAT2;proline dehydrogenase 1, mitochondrial isoform X1;CNDP2;GAMT;PYCR3;prolyl 4-hydroxylase subunit alpha-2 isoform X1	AMD1;SAT1;OAT;SMOX;prolyl 4-hydroxylase subunit alpha-2 isoform X1
oas00340	Histidine metabolism	CNDP2;aldehyde dehydrogenase family 3 member B1 isoform X1;ALDH3B1	CARNMT1
oas00350	Tyrosine metabolism	TOMT;FAH;aldehyde dehydrogenase family 3 member B1 isoform X1;ALDH3B1;FAHD1;MIF	L-amino-acid oxidase-like
oas00480	Glutathione metabolism	GPX1;glutathione S-transferase Mu 1;GPX4;GSTP1	GCLM;RRM2B
**Glycan metabolism**			
oas00510	N-glycan biosynthesis	ALG5;ALG12;DOLK;ALG3;DPM2	B4GALT1;ALG2;B4GALT3;dol-P-Glc:Glc(2)Man(9)GlcNAc(2)-PP-Dol alpha-1,2-glucosyltransferase;MAN1A2;ALG6;MGAT4A;putative bifunctional UDP-N-acetylglucosamine transferase and deubiquitinase ALG13 isoform X1;MAN2A1;MGAT3
oas00514	Other types O-glycan biosynthesis	RFNG;ST3GAL3;COLGALT1;POMT1	B4GALT1;B4GALT3;OGT;EOGT
oas00531	Glycosaminoglycans degradation	HEXA;NAGLU;IDUA;beta-hexosaminidase subunit beta-like isoform X1;SGSH	
oas00532	Glycosaminoglycans biosynthesis	B4GALT7;B3GAT3	XYLT1;CSGALNACT1
oas00563	Glycosylphosphatidylinositol biosynthesis	PIGQ;GPAA1;PIGL;PIGU;DPM2	PIGA;PGAP1
oas00601	Glycosphingolipids metabolism	ST3GAL6;ST3GAL3	ST3GAL4;B4GALT1;B4GALT3;N-acetyllactosaminide beta-1,6-N-acetylglucosaminyl-transferase isoform X2;ST3GAL6
**Vitamins and cofactors metabolism**			
oas00740	Riboflavin metabolism	ACP5;FLAD1;BLVRB	RFK; ACP1
oas00750	Vitamin B6 metabolism	PDXK	
oas00760	Nicotinate and nicotinamide metabolism	NT5C3B;NAPRT;NMNAT1;BST1	NMNAT3;NT5C2;NUDT12;NADK2;NMRK1;NAMPT
oas00770	Pantothenate and CoA biosynthesis		PPCS;PANK3;DPYD
oas00790	Folate biosynthesis	SPR	

oas00830	Retinol metabolism	dehydrogenase/reductase SDR family member 4;cytochrome P450 2S1 isoform X2	LRAT;RDH11
**Genetic information processing**			
oas03020	RNA polymerase	POLR3H;POLR2E;POLR2I	POLR1B
oas03022	Basal transcription factor	GTF2H4;GTF2F1;TAF10;ERCC2	TAF13;TAF7;CCNH;TAF8;TAF5;GTF2H3;general transcription factor IIH subunit 2 isoform X2;TAF9;CDK7;TAF4;TAF1
oas03050	Proteasome	PSMD4;PSME2;PSMB8;PSMC5;PSMD8;PSMB10;PSME1;PSMC3;PSMC1;PSMB3	PSME4;PSMD12;IFNG
oas03040	Spliceosome	SNRPA;PRPF31;PPIH;HNRNPA3;BUD31;PQBP1;SNRNP70;PUF60;LSM2;SF3B5;SRSF1;SF3A2;small nuclear ribonucleoprotein F-like	SRSF3;TRA2A;RBM25;CRNKL1;PLRG1;RBMX;AQR;DDX46;DHX15;NCBP1;U2SURP;HNRNPA3;DDX5;SRSF6;WBP11;TCERG1;SRSF4;HNRNPC;DHX8;CDC40;TRA2B;SNW1;PRPF40A;PRPF8;SLU7;SRSF7;SRSF5;SRSF10;HNRNPA1;SRSF1;LSM5;PRPF38B
oas03010	Ribosome	RPL13A;RPS6;RPS11;RPSA;RPL14;RPL19;RPS14;RPS9;60S ribosomal protein L6;60S ribosomal protein L27;RPLP2;MRPL36;MRPL23;RPS19;RPLP1;MRPL27;MRPL24;RPL36;40S ribosomal protein S2;MRPL28;RPL7A;60S ribosomal protein L10-like;RPL12;RPL8;RPS5;RPL18A;MRPL2;RPL29;MRPS12;MRPS15;MRPL11;MRPL21;MRPS18A;MRPL14;MRPS5;60S ribosomal protein L5;60S ribosomal protein L21-like;RPL28;RPL11;MRPL13;MRPS11;RPL18;60S ribosomal protein L23a;uncharacterized protein LOC105605134;RPS26;60S ribosomal protein L17;RPL13;UBA52;RPS2	RPL6;RPS27
oas00970	Aminoacyl-tRNA-biosynthesis	GATB;VARS;CARS2;FARS2;MARS	IARS;LARS;KARS
oas04130	SNARE interaction in vesicular transport	VAMP5;VAMP8;STX4;BET1L;STX8;USE1;STX5	VAMP7;GOSR1;SEC22B;BET1;STX16;SNAP23;STX7;vesicle-associated membrane protein 3

oas03013	RNA transport	PABPC1;EIF1AX;NXT1;PYM1;SNUPN;RPP25;THOC5;RPP21;EIF4EBP1;EIF3G;EIF3B;PABPC5;EIF3I;GEMIN8;EIF4A1;EIF4EBP3	EIF4A2;EIF3A;UPF3B;PNN;NUP210L;NMD3;NCBP1;NUP107;NUP54;NUPL2;TPR;XPOT;UBE2I;EIF4E;CYFIP2;elongation factor 1-alpha 1;XPO5;TGS1;RNPS1;NUP58;SENP2;UPF2;E3 SUMO-protein ligase RanBP2 isoform X1;NUP35;FMR1;EIF3E;NDC1;EIF4G2;NUP43
oas03018	RNA degradation	PABPC1;PABPC5;DCPS;EXOSC6;EXOSC4;LSM2;CNOT2;PFKL;NUDT16	BTG1;TOB2;XRN2;TOB1;XRN1;CNOT8;DIS3-like exonuclease 1;PARN;DHX36;EXOSC2;MTREX;CNOT6;CNOT7;LSM5;CNOT2
oas03015	mRNA surveillance pathway	PABPC1;NXT1;PYM1;PABPC5	UPF3B;PNN;MSI2;NCBP1;eukaryotic peptide chain release factor subunit 1;SMG7;PPP1CC;PCF11;PPP2R5E;WDR33;PPP2R1B;HBS1-like protein;PPP2R2A;SMG1;PPP2CB;FIP1L1;RNPS1;UPF2;PPP2R5A;CSTF2T;NUDT21;PAPOLA;CSTF3;PPP2CA
**Signal transduction**			
oas04010	MAPK signaling pathway	MAP3K12;CSF1;HRAS;TRAF2;MAP3K11;MAP4K4;CSF1R;MAPK8IP3;TRADD;PAK1;MAP2K2;HSPB1;VEGFB;MAPK12;MAPK11;MAPKAPK2;MAPK8IP2;ECSIT;FLNA;MAX;IRAK1;FLNB;JUND;NFKB2;IGF1	RASGRP3;NRAS;SOS2;DUSP4;RAP1B;TGFA;MAP3K7;PPP3CB;RASA2;ATF2;TAOK1;PRKCA;RPS6KA3;RAPGEF2;PPM1B;KRAS;MAP2K4;CRK;RPS6KA5;MAPK8;RASA1;AKT2;PAK2;CASP3;FLT1;PRKACA;VEGFA;MYC

oas04152	AMPK signaling pathway	CCND1;CRTC2;RPS6KB2;EIF4EBP1;G6PC3;CREB3L3;PFKL;CPT1B;IGF1;PPARG	FOXO3;platelet glycoprotein 4-like isoform X1;SIRT1;EEF2K;RPS6KB1;PPP2R5E;MAP3K7;PPP2R1B;HMGCR;PIK3CA;PPP2R2A;PRKAB2;CREB3L1;PPP2CB;CAB39L;PDPK1;PPP2R5A;AKT2;PIK3R1;PPP2CA;SCD;LEPR
oas04910	Insulin signaling pathway	RPS6;PRKAR1A;HRAS;FLOT1;EXOC7;FLOT2;PRKAR1B;RPS6KB2;EIF4EBP1;G6PC3;MAP2K2;CALM3;SH2B2;PYGB;BAD	GSK3B;NRAS;SOS2;PRKAR2B;PPP1CC;RPS6KB1;PIK3CA;PHKB;PHKA1;PTPRF;EIF4E;PRKAB2;SHC1;KRAS;E3 ubiquitin-protein ligase CBL-B;SOCS2;CRK;MAPK8;PDPK1;SOCS4;AKT2;PIK3R1;PRKACA
oas04060	Cytokine-cytokine receptor interaction	CNTFR;CSF1;GDF1;TNFSF9;TNFRSF18;CSF1R;CXCR2;LTBR;C-C motif chemokine 3;granulocyte-macrophage colony-stimulating factor receptor subunit alpha isoform X1;TNFRSF6B;TNFRSF11A;TNFRSF13B;IL17RE	BMPR2;LIFR;CXCL11;IL6ST;CCR3;ACVR1;LEPR;ACVR2A;GHR;IFNG;CCR9;TNFSF13B
oas04150	mTOR signaling pathway	RPS6;RHOA;TTI1;HRAS;LAMTOR4;ATP6V1B1;RPS6KB2;EIF4EBP1;FZD1;ATP6V1F;FMAP2K2;FZD9;SLC38A9;MLST8;LAMTOR2;IGF1	GSK3B;RRAGA;RHOA;ATP6V1A;NRAS;SOS2;RICTOR;RPS6KB1;RRAGB;SKP2;PIK3CA;PRKCA;RPS6KA3;folliculin-interacting protein 2 isoform X1;EIF4E;KRAS;SLC38A9;CAB39L;PDPK1;FZD5;AKT2;PIK3R1;WNT4
oas04020	Calcium signaling pathway	ORAI1;SPHK1;PTGER3;ITPR1;PLCD1;TPCN1;TPCN2;PLCB3;CALM3;GRIN1;MYLK	ADRB2;ATP2A2;F2R;STIM2;SPHK2;PPP3CB;CAMK1D;PRKCA;PHKB;PHKA1;GNAQ;inositol 1,4,5-trisphosphate receptor type 2;CYSLTR2;MCU;PRKACA

oas03320	PPAR signaling pathway	ACSL6;ACAA1;RXRB;SLC27A1;NR1H3;apolipoprotein A-I;SLC27A4;CPT1B;PPARG	platelet glycoprotein 4-like isoform X1;PDPK1;CPT2;ACSL5;SCD;LPL;PPARA
oas04310	Wnt signaling	CCND1;CSNK2B;RHOA;FZD1;RUVBL1;FZD9;PLCB3	CCND3;GSK3B;RHOA;PSEN1;APC;CUL1;MAP3K7;PPP3CB;CHD8;PRKCA;LGR4;SIAH1;MAPK8;SENP2;DKK1;FZD5;CSNK1A1;WNT4;CSNK2A2;PRKACA;SMAD4;BAMBI
oas04015	Rap1 signaling pathway	RHOA;CSF1;HRAS;RAP1GAP;LPAR2;RAPGEF3;CSF1R;MAP2K2;PARD6A;VEGFB;MAPK12;PLCB3;MAPK11;CALM3;RASSF5;FARP2;GRIN1;IGF1	RHOA;RASGRP3;NRAS;F2R;RAP1B;PIK3CA;PRKCA;RAPGEF2;GNAQ;KRAS;CRK;PARD6B;MAGI3;AKT2;CDH1;PIK3R1;SIPA1L1;FLT1;VEGFA;ITGB1; ITGAL
oas04115	p53 signaling pathway	CCND1;BID;TP73;COP1;BAX;IGF1	APAF1;CCND3;SESN3;GORAB;bcl-2-like protein 1;ATR;PPM1D;CCNG2;SIAH1;CHEK1;PERP;SESN1;DDB2;CCNG1;RRM2B;bcl-2-like protein 1;cytochrome c;CASP3
oas04151	PI3K-Akt signaling pathway	RPS6;CCND1;BCL2L11;CRTC2;GNB2;CSF1;HRAS;LPAR2;COL6A2;RPS6KB2;CSF1R;EIF4EBP1;G6PC3;CREB3L3;MAP2K2;MLST8;VEGFB;ITGB4;ITGA5;IGF1;BAD	CCND3;GSK3B;FOXO3;NRAS;SOS2;PIK3CG; GNB1;MTCP1;PHLPP2;F2R;bcl-2-like protein 1;TGFA;RPS6KB1;PPP2R5E;PPP2R1B;ATF2;MYB;PIK3CA;PRKCA;PPP2R2A;JAK1;EIF4E;CREB3L1;JAK2;KRAS;LPAR6;ITGA1;RBL2;PPP2CB;PDPK1;PPP2R5A;ITGA10;YWHAQ;PKN2;AKT2;PIK3R1;bcl-2-like protein 1;PPP2CA;FLT1;VEGFA;ITGB1;ITGAV;YWHAE;YWHAH;GHR;MYC;ITGA4;YWHAZ

oas04070	Phosphatidyl inositol signaling	MTMR14;ITPR1;PLCD1;PLCB3;CALM3;CDIPT	PIP4K2A;MTMR4;MTMR3;IMPAD1;PIK3CA;PRKCA;INPP4B;inositol 1,4,5-trisphosphate receptor type 2;PIP5K1A;MTM1;PIK3C2A;MTMR6;SACM1L;PIP4K2C;INPP5B;DGKE;PIK3R1;INPP5F
**Cellular processes**			
oas04141	Protein processing in endoplasmatic reticulum	CALR;TRAF2;DNAJC5;SSR4;BCAP31;F-box only protein 6;WFS1;PREB;OS9;PRKCSH;UFD1;RRBP1;SSR2;UBE2D4;BAX;BAK1;CAPN1	DERL1;UBQLN1;ERN1;SAR1B;endoplasmic reticulum lectin 1;TRAM1;CUL1;UBE2J2;EDEM3;MAN1A2;SAR1A;EIF2AK3;UBE2J1;UBE2G1;SEL1L;DNAJA2;CKAP4;EIF2AK1;MAPK8;LMAN1;SEC63;SEC24A;AMFR;SEC24C;UFD1;HERPUD1;NGLY1;CANX
oas04142	Lysosome	NPC2;PSAP;HEXA;NAGA;SMPD1;ACP5;CTSZ;NAGLU;CD68;M6PR;NAGPA;GM2A;IDUA;CTSH;GNPTG;ARSG;LITAF;ABCA2;beta-hexosaminidase subunit beta-like isoform X1;CLN3;TCIRG1;CTSA;CD63;NAPSA;CLTB;GBA;SGSH;MCOLN1;ARSA;AP1S1;ATP6V0A1;SLC11A1;CTSV	AP1G1;CLTA;CTSO;natural resistance-associated macrophage protein 2 isoform X1;MFSD8;SLC17A5;IGF2R
oas04530	Tight junction	CCND1;RHOA;PARD6A;CLDN15;MYL9	RHOA;ROCK1;WHAMM;MYH9;RDX;PPP2R1B;PPP2R2A;PRKCE;EZR;RAPGEF2;PRKAB2;PPP2CB;MAPK8;PARD6B;ARHGAP17;NEDD4L;PPP2CA;PRKACA;ITGB1;PCNA
oas04520	Adherens junction	CSNK2B;RHOA;FARP2	RHOA;LMO7;MAP3K7;PTPRF;SMAD2;CDH1;VCL;CSNK2A2;SMAD4

Figure S1. Heatmap of gene expression of FADS isoforms.

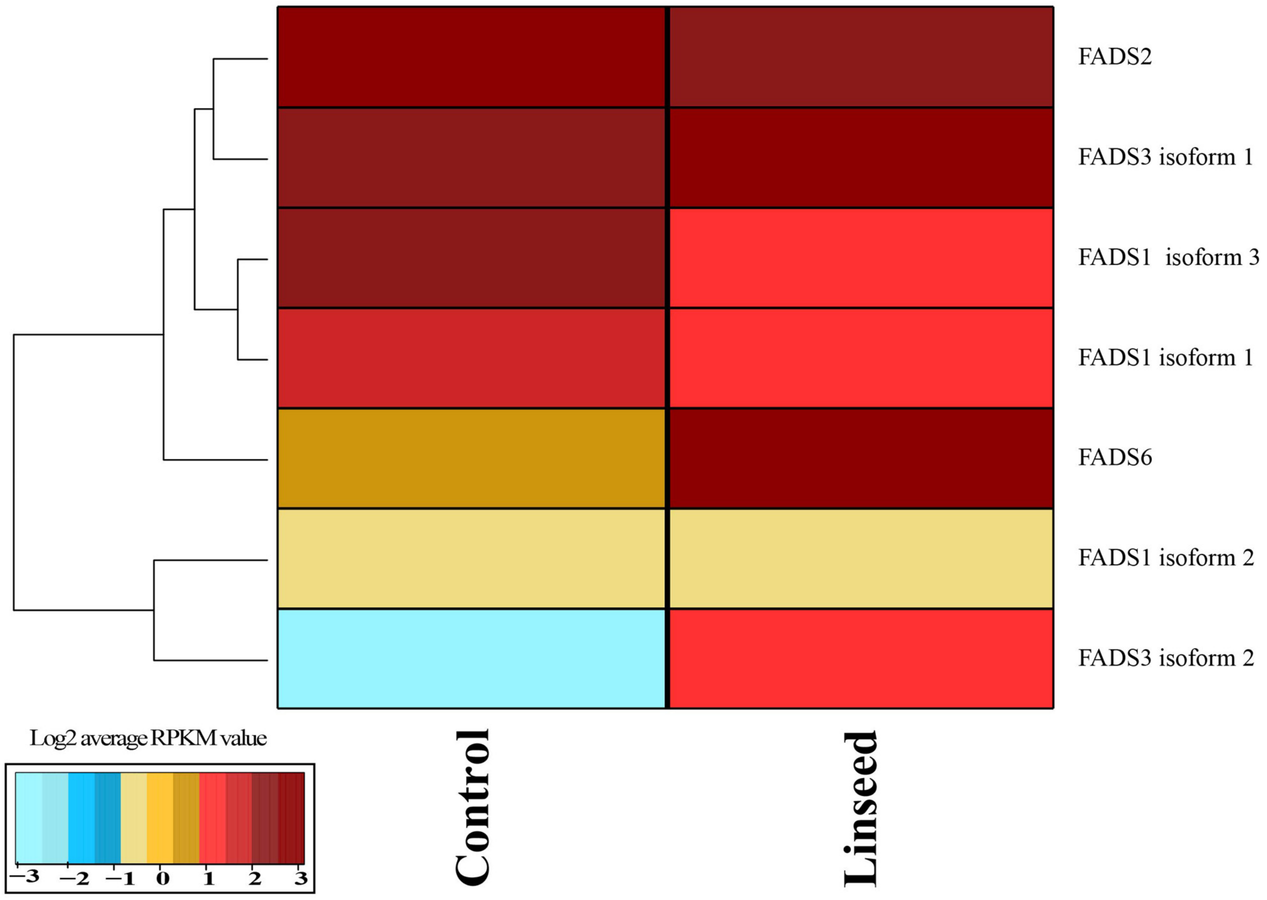

Supplement: Supplementary file 1 [file animals-11-02707-s001.zip › animals-1338051-supplementary.pdf]
